# 3DLigandSite: structure-based prediction of protein–ligand binding sites

**DOI:** 10.1093/nar/gkac250

**Published:** 2022-04-12

**Authors:** Jake E McGreig, Hannah Uri, Magdalena Antczak, Michael J E Sternberg, Martin Michaelis, Mark N Wass

**Affiliations:** School of Biosciences, Division of Natural Sciences, University of Kent, Canterbury, Kent CT2 7NJ, UK; School of Biosciences, Division of Natural Sciences, University of Kent, Canterbury, Kent CT2 7NJ, UK; School of Biosciences, Division of Natural Sciences, University of Kent, Canterbury, Kent CT2 7NJ, UK; Centre for Integrative Systems Biology and Bioinformatics, Department of Life Sciences, Imperial College London, London SW7 2AZ, UK; School of Biosciences, Division of Natural Sciences, University of Kent, Canterbury, Kent CT2 7NJ, UK; School of Biosciences, Division of Natural Sciences, University of Kent, Canterbury, Kent CT2 7NJ, UK

## Abstract

3DLigandSite is a web tool for the prediction of ligand-binding sites in proteins. Here, we report a significant update since the first release of 3DLigandSite in 2010. The overall methodology remains the same, with candidate binding sites in proteins inferred using known binding sites in related protein structures as templates. However, the initial structural modelling step now uses the newly available structures from the AlphaFold database or alternatively Phyre2 when AlphaFold structures are not available. Further, a sequence-based search using HHSearch has been introduced to identify template structures with bound ligands that are used to infer the ligand-binding residues in the query protein. Finally, we introduced a machine learning element as the final prediction step, which improves the accuracy of predictions and provides a confidence score for each residue predicted to be part of a binding site. Validation of 3DLigandSite on a set of 6416 binding sites obtained 92% recall at 75% precision for non-metal binding sites and 52% recall at 75% precision for metal binding sites. 3DLigandSite is available at https://www.wass-michaelislab.org/3dligandsite. Users submit either a protein sequence or structure. Results are displayed in multiple formats including an interactive Mol* molecular visualization of the protein and the predicted binding sites.

## Graphical Abstract

**Graphical Abstract:**
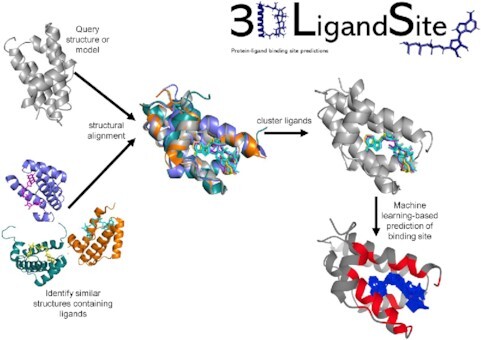
Overview of 3DLigandSite: a web server for prediciting small molecule binding sites in proteins.

## INTRODUCTION

Elucidation of protein function remains a difficult and important task, with many millions of proteins present in UniProt (1) and only a small fraction of them functionally annotated (2,3), making automated sequence annotation tools essential. Small molecules that bind to proteins are intimately related to protein function; they can be substrates or products of an enzyme reaction, cofactors (4) that play an essential role in catalysis or have important structural or regulatory roles (5).

Methods for predicting ligand-binding sites [reviewed in (6)] use a range of different approaches, including sequence conservation (7), structural approaches such as identifying pockets on the protein surface, the combined analysis of sequence and structural information (8), and machine and deep learning (9–16). 3DLigandSite and methods such as firestar (17), FINDSITE (18,19), COACH-D (20) and FunFOLD2 (21) utilize knowledge of existing binding sites in solved protein structures present in the Protein Data Bank (PDB) (22). 3DLigandSite, FINDSITE, FunFOLD2 and COACH-D combine the modelling of protein structure with the identification of homologous proteins in the PDB that have ligands bound to them. These binding sites are then used to infer binding sites in the query protein. By contrast, firestar uses FireDB (23), a database of ligand-binding residues extracted from protein structures in the PDB and also catalytic residues extracted from the Catalytic Site Atlas (24).

Here, we present the first major update to the 3DLigandSite web server. 3DLigandSite was first developed in 2010 (25) to automate an approach that was successfully used in the ligand-binding site experiment in the eighth round of the critical assessment of protein structure prediction (CASP) community experiment (26,27). Over the last 12 years, 3DLigandSite has become widely used, attracting an average of 125 000 submissions per year, for a diverse range of purposes, including genome annotation (28,29), antiviral screening (30), the analysis of single-nucleotide variants (SNVs) associated with disease (31–35), the development of fluorescent sensors (36) and most recently for analysis of SARS coronavirus-2 proteins (37–39). Over the last 3 years, 3DLigandSite binding site predictions have been incorporated into the Protein Data Bank in Europe [PDBe (22)] Knowledgebase (40,41), making binding site predictions for protein structures in the PDBe widely available.

The basic 3DLigandSite algorithm remains the same in the new version, but it now makes use of the latest sequence searching methods and the highly accurate protein 3D structural models available from the AlphaFold Protein Structure Database [AlphaFold DB (42,43)]. 3DLigandSite now also incorporates machine learning as the final step in the prediction process, which improves prediction accuracy and associates a confidence score with each individual residue predicted to be part of a binding site. This is combined with a new web server that offers improved functionality for users to investigate the predicted binding sites.

## THE 3DLigandSite METHOD

**Figure 1.**
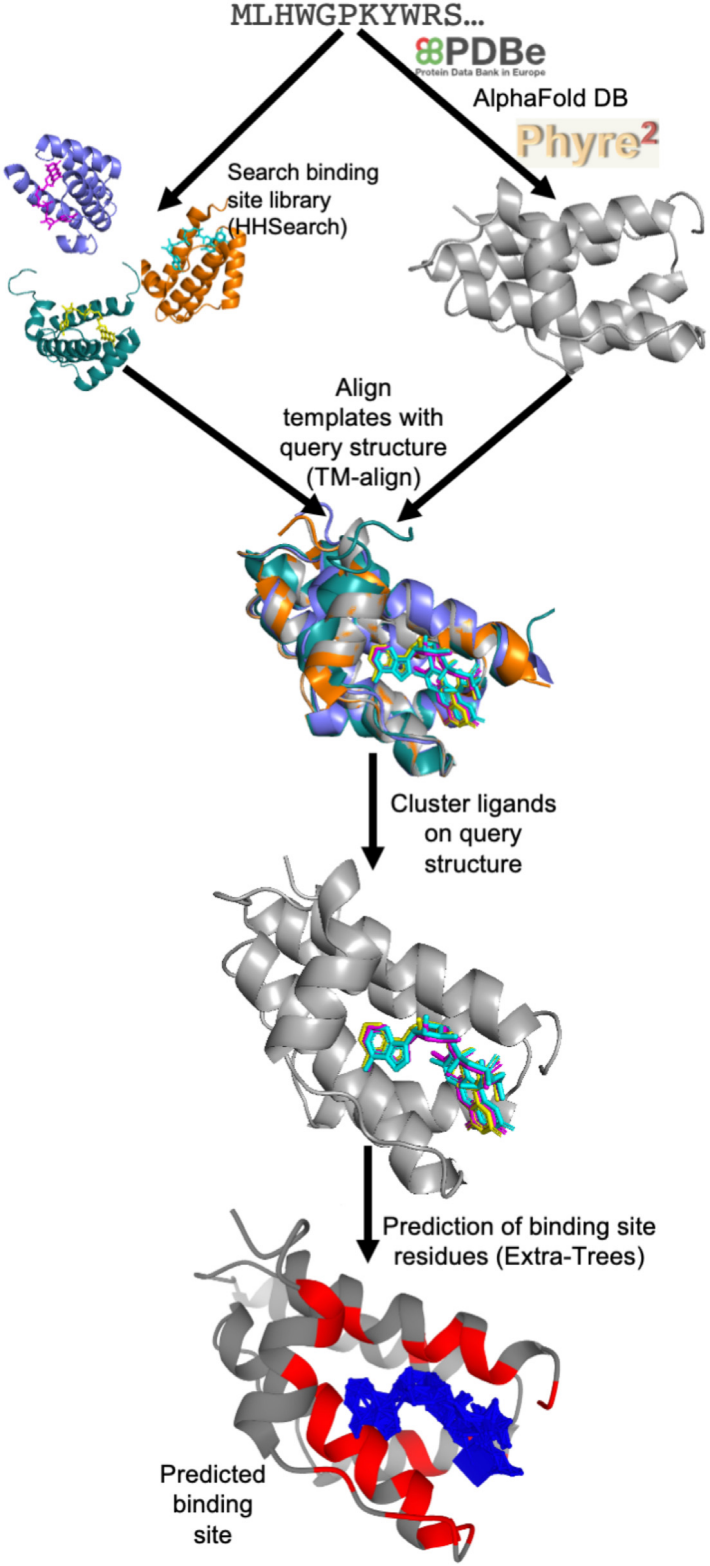
An overview of the 3DLigandSite method. Users submit either a protein sequence or structure. Where sequences are submitted, the PDBe and AlphaFold DB are searched for a matching structure; where one is not available, Phyre2 is used to model the 3D structure. HHSearch is used to search a sequence library of protein structures with ligands bound. Hits from this search are aligned with the structure of query protein, and the ligands from these structures are clustered. Each cluster of ligands represents a potential binding site in the query protein. A machine learning classifier is used to predict which of the residues around the cluster are likely to form part of a binding site.

A summary of the 3DLigandSite methodology is outlined in Figure 1. Users submit either a protein sequence in FASTA format or a protein structure in PDB format. Where a sequence is submitted, the PDB is first searched for an existing structure with identical sequence that can be used. Where a match is not found, AlphaFold DB (42) is searched for an existing structural model. Finally, where a model is not available, Phyre2 (44) is used to perform template-based modelling.

The next step focuses on the identification of ligand-bound structures that are homologous to the query protein. Originally, 3DLigandSite used MAMMOTH (45) to perform a structural search of the query structure against a structural library of proteins from the PDB, which was a time-consuming step, typically taking between 40 and 80 min. This has been replaced by a sequence-based search using HHSearch (46) to screen a sequence library of ligand-bound proteins from the PDB (detailed later), which only takes a few minutes to run. All sequence matches with an HHSearch probability score >75% are retained, and their protein structures are aligned to the query structure using TM-align (47). The user can reduce the HHSearch probability cut-off if they would like to use less confident matches to the query sequence.

Where matches to the library of ligand-bound proteins are not identified by the sequence-based search, a structural search is performed using TM-align (47) that retains alignments with a TM-score of 0.6 or greater. The structural search is also available as an advanced option that users can choose to perform at the time of submission.

The ligands present in the library structures are superimposed onto the query structure by aligning the library structures with the query structure. These ligands are then clustered. Originally, 3DLigandSite used single linkage clustering to cluster ligands, which could result in very large clusters. To avoid this, 3DLigandSite now generates clusters such that 50% of each ligand must overlap with at least one of the other ligands in the same cluster. This change also required that metal and non-metal ligands are separately clustered given that metal ligands are single ions, while non-metal ligands are larger molecules (e.g. ATP, NAD). Individual predictions of metal and non-metal binding sites are also made for these separate clusters.

The final step of the prediction process is to determine the residues in the protein that are predicted to form the binding site associated with each cluster of ligands. Each cluster may contain multiple different ligands or many instances of the same (or similar) ligands in different poses. 3DLigandSite originally predicted any residue within 0.8 Å of at least 25% of the ligands in a cluster to be part of the binding site. This has been replaced by the introduction of a logistic regression classifier (detailed below) to perform this final prediction step. This also associates a confidence score (range 0–1) with each residue in the predicted binding site.

### Generation of the library of ligand-bound protein structures

To generate the library of biologically relevant protein binding sites, protein structures were extracted from the PDB and filtered to retain only those containing ligands classed as cognate by FireDB (23). The library focuses on monomeric proteins. Where binding sites were located in the interface between two proteins, the multimer was split into monomeric structures and the ligands associated with both of the monomers. The protein structures were clustered, and the ligands from proteins in each cluster mapped onto a representative structure to reduce search time. The amino acid sequences of the retained structures were clustered using CD-HIT (48) using an 80% sequence identity threshold. The protein models in each cluster were then aligned to the cluster representative (obtained from CD-HIT) using TM-align (47), and the ligands were superimposed onto the representative structure and retained. An HHSearch (49) sequence database was built from the representative sequences for searching user-submitted protein sequences against.

### Calculating residue conservation

To calculate residue conservation, HHblits (50) was used to search the query sequence against the UniClust30 database (51). The multiple sequence alignment was then used to calculate the Jensen–Shannon divergence (52) conservation score.

### Machine learning-based prediction of binding site residues

The machine learning step was introduced to predict accurately which residues are most likely to be part of the binding site around a cluster of ligands. An equal number of binding and non-binding residues on the query protein were used for training and testing. For each of these residues, a set of features was extracted and converted to a 0–1 range (Supplementary Table S1). Several features were considered for best determining binding propensity. The features included distance measurements to the ligand cluster, residue conservation and amino acid properties such as charge, hydrophobicity and van der Waals volume (Supplementary Table S1). Solvent accessibility scores were obtained from ProAct2 (53). Distance-based features were calculated, including the minimum, maximum and average distances of each residue to ligands in the cluster, and the percentage of ligands in the cluster within 0.8 Å + van der Waals radii of the amino acid.

Univariate feature selection was used to identify ligand contacts. The three distance features and residue conservation were the most informative features for predicting ligand binding, as well as the negative charge residue feature for metal binding sites. A single distance metric was selected to avoid overtraining on a similar feature, resulting in the ligand contacts, minimum ligand distance, negatively charged and residue conservation as the selected features.

The scikit-learn Python package was used to train support vector machines (54), Extra-Trees, logistic regression and random forest classifiers. The data were then fitted with optimum parameters from 100 random iterations and three cross-validation steps using GridSearchCV within scikit-learn. A randomly generated 80:20 train–test split was used to fit the models.

The training and test sets comprise monomers with cognate ligands bound. These structures were identified by filtering the PDB, clustering their sequences using MMseqs2 (55) at a maximum sequence identity of 40%. This resulted in 5223 metal and 4995 non-metal binding sites. A subset of 1600 metal and 1573 non-metal binding sites was randomly selected for testing and training. The remaining binding sites were used as a validation set to evaluate performance on the trained classifiers (that had not been used in training) (Supplementary Figure S1). The PDB identifiers and chains of all sequences used are provided in Supplementary Table S2.

Binding residues were classed as all residues within van der Waals radii + 0.8 Å of the ligand present in the protein structure, with all other residues classed as non-binding. This resulted in 1976 and 6950 metal and non-metal binding residues, respectively, and an equal number of randomly selected non-binding residues were also randomly extracted (Supplementary Table S3), providing the positive and negative examples required for training the machine learning classifiers.

## EVALUATING 3DLigandSite PERFORMANCE

The performance of 3DLigandSite was assessed using the validation set (see the ‘The 3DLigandSite Method’ section), which contained 59 203 and 16 166 (Supplementary Table S3) non-metal and metal binding residues, respectively, that had not been used in the testing or training of the classifiers. Performance was assessed using multiple measures of precision, sensitivity (recall) and the receiver operator characteristic (ROC).

**Figure 2.**
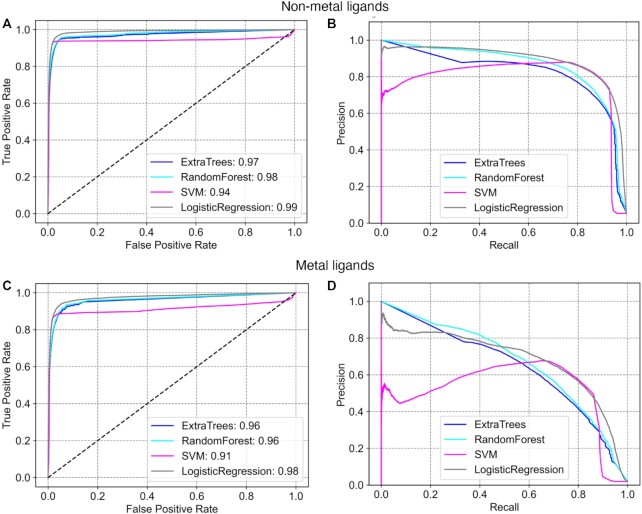
Benchmarking the 3DLigandSite machine learning classifier. ROC curves and precision–recall curves are shown for the prediction of binding sites of non-metal (**A** and **B**) and metal (**C** and **D**) ligands.

The logistic regression classifier performed best on the non-metal binding sites, with an area under the receiver operating characteristic curve (AUROC) of 0.99, though a similar performance was observed for Extra-Trees and random forest classifiers (Figure 2A and Supplementary Table S4). For metal ligands, the logistic regression classifier performed best with an AUROC of 0.99 (Figure 2C and Supplementary Table S4). As the data set has a skewed distribution with many more negative examples than positive examples (i.e. many non-binding residues compared to those that are binding residues in each protein; Supplementary Table S3), precision–recall metrics provide a better indication of performance (56,57). 3DLigandSite obtained 92% recall at 75% precision for non-metal binding sites (Figure 2B) and 52% recall at 75% precision for metal binding sites (Figure 2D). We compared the performance of the new version of 3DLigandSite with the original version (25). The original 3DLigandSite did not make separate predictions for metal and non-metal ligands, so we assessed performance on the combined metal and non-metal binding sites. On the validation set, the original 3DLigandSite obtained recall of 56% at 59% precision.

The performance of 3DLigandSite was also evaluated on the 70 targets used for assessment of binding site prediction in CASP8 (26), CASP9 (58) and CASP10 (59). Using the sequence-based homology search 3DLigandSite obtained recall of 85% at 65% precision, and a Matthews’ correlation coefficient [MCC (60)] of 0.73. Performance using the structural search option was comparable, with slightly lower recall of 80% at 67% precision and an MCC of 0.72. Structural search results at a range of TM-score thresholds for inclusion of template structures are shown in Supplementary Table S5. On this data set, the sequence-based search was not inferior to the structure-based search, although recent studies have suggested that structural searches are better at identifying related protein structures (61,62). Given, the extra time taken to perform the structural search (∼4 h per submission), the sequence search is recommended and is the default for the web server.

## THE 3DLigandSite WEB SERVER

The 3DLigandSite web server is available at https://www.wass-michaelislab.org/3dligandsite. The web server is free to all without a login requirement. Users can select to submit either a protein sequence (in FASTA format) or a protein structure (in PDB format). Where a sequence is submitted, the first step of the prediction process is to obtain a model of the protein structure. To do this, the PDB is first searched for a matching structure, followed by AlphaFold DB (42). Where a suitable model is not available in either database, Phyre2 (44) is used to generate a template-based model of the structure. The runtime for submissions that require Phyre2 is longer as modelling the protein structure is time-consuming, typically taking a few hours to complete. Where users submit a protein structure, the runtime is typically <5 min using default settings. Users who provide an email address receive an email upon submission and once their results are ready for viewing. The web server includes a help section that includes recordings that work users through both the submission process and interpretation of data in the Results pages.

### Results’ output

**Figure 3.**
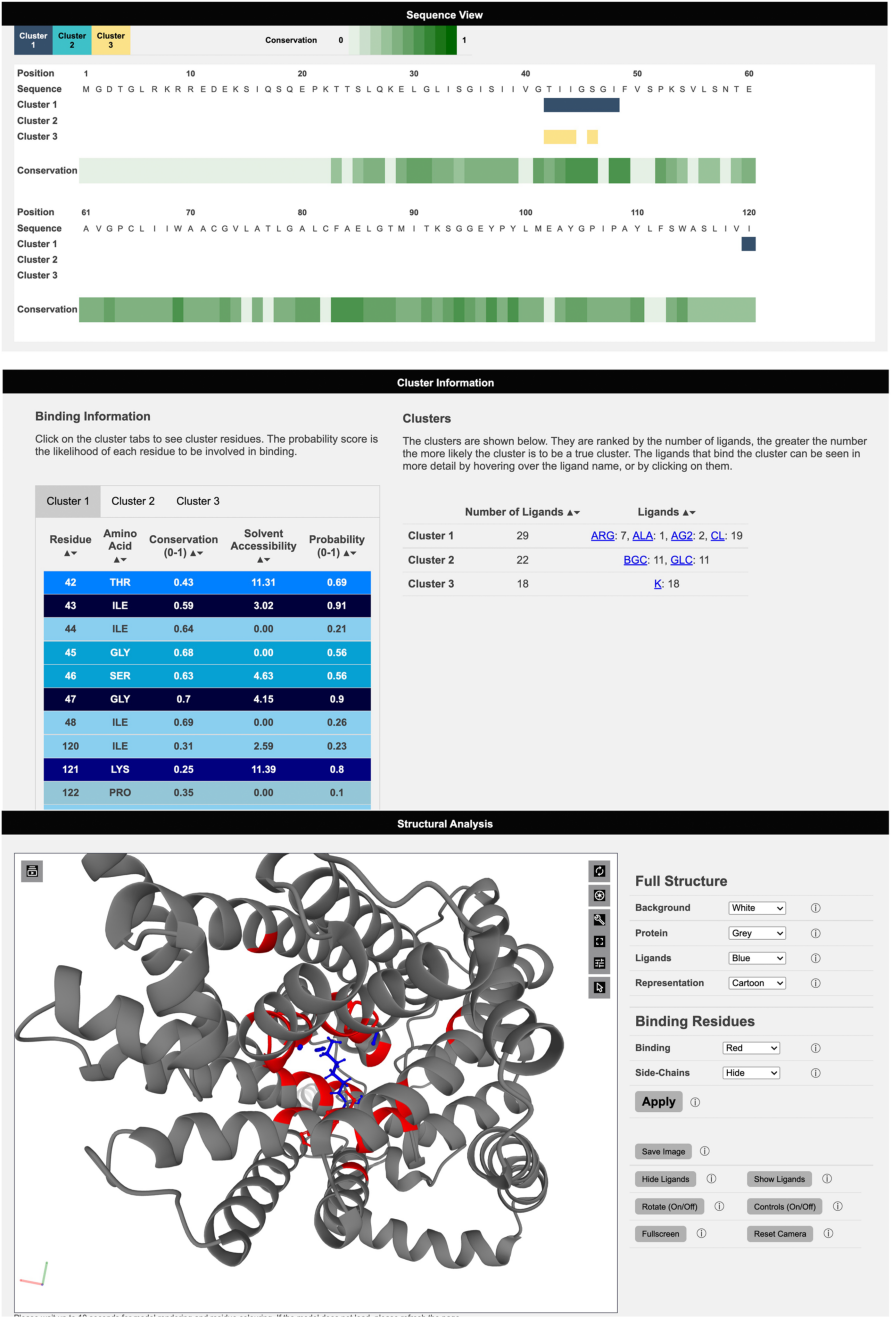
Viewing results on the 3DLigandSite web server. Results are presented in three main sections: a sequence view, which maps sequence conservation and the different clusters identified onto the protein sequence. Second, details of the clusters, including the number of ligands and type of ligand, are displayed as well as a table listing the residues predicted to form the binding site for each cluster. Finally, the structural analysis section includes a Mol* molecular viewer to visualize the protein, the predicted binding site and the clusters used to make the predictions. A separate control panel (on the right) enables users to easily modify the display.

3DLigandSite Results pages are split into three main sections. Results are initially presented as a sequence view (Figure 3), which shows the amino acid sequence of the submitted protein, residue conservation and a row for each cluster of ligands that has been identified as a potential binding site (Figure 3). This provides users with an easily interpretable view of the predicted binding sites.

The second section of results shows the cluster table, which includes details of the clusters identified, the number of ligands present in each cluster and the number of structures that these ligands originate from. The ligands are represented by the three-letter codes from the mmCIF dictionary and are linked to the small molecule details in the PDB (Figure 3). Clusters are sorted according to the number of ligands present in the cluster. There is greater confidence that a cluster represents a binding site when there is evidence for this from multiple protein structures. The second table in this section contains a tab for each ligand cluster and lists the residues predicted to be in the binding site along with the conservation score, solvent accessibility and the probability calculated by the logistic regression classifier.

The final section of the Results page contains a Mol* molecular viewer (www.molstar.org) (63) that by default displays the protein structure in a cartoon format along with the ligands in the top-ranked cluster, highlighting the predicted binding site residues in red (Figure 3). The Mol* viewer enables users to inspect the predicted binding sites within the protein structure and offers multiple features for exploring the structure. The 3DLigandSite control panel to the right of the viewer provides easy-to-use functions such as changing the colour or format of the display of the ligands and the protein structure. Further functionality is available via the Mol* built-in options shown on the top right of the viewer. The control panel also includes a button enabling users to generate publication-quality images of the current display in the viewer.

## USE CASES

As set out in the ‘Introduction’ section, 3DLigandSite predictions have been widely used for a range of different biological and biomedical purposes. For example, with widespread use of sequencing technologies, there is extensive interest in the analysis of non-synonymous SNVs (nsSNVs). The aim here is to identify those nsSNVs that may alter protein structure and function and be associated with a phenotype such as a disease. Thus, 3DLigandSite has been used to analyse such nsSNVs for a range of diseases, from liver disease (31) to cardiomyopathies (33).

One application has been to study nsSNVs present in individuals with cystinuria, which is caused by variants in two genes, SLC7A9 and SLC3A1, that encode a dimeric amino acid transporter (64). Cystinuria is caused by variants that affect the ability of this transporter to transport cystine into cells, which results in the formation of kidney stones. In a recent study (34,65), 3DLigandSite was used to model the structure and ligand-binding sites of the two encoded proteins and to analyse how the set of nsSNVs observed in a cohort of patients may affect transporter function and be linked with the severity of the disease that patients experienced. Figure 3 shows the protein b(0+)AT, which is encoded by SLC7A9, and the predicted amino acid binding sites in the protein.

## CONCLUDING REMARKS

The 3DLigandSite web server provides free access to an easy-to-use resource for modelling small molecule binding sites in proteins. This widely used resource has been extensively updated to offer improved functionality and to reduce the runtime of user submissions. Our benchmarking demonstrates that 3DLigandSite can obtain high recall with high precision, therefore accurately predicting binding sites in proteins that users are researching.

## DATA AVAILABILITY

All data are provided in the manuscript or supplementary material.

## Supplementary Material

gkac250_Supplemental_Files
