# Supplementary material for: 3DLigandSite: Structure-based prediction of protein-ligand binding sites
Source: Nucleic Acids Res. Author manuscript; Available in PMC 2022 Jul 6. (PMC9252821; doi:10.1093/nar/gkac250)
Supplement: Supplementary Table 2 [file EMS144313-supplement-Supplementary_Table_2.pdf]

Supplementary Table 2. List of all Protein Databank identifiers used with protein chain included
11as_A
11as_B
155c_A
1a0i_A
1a0t_R
1a2b_A
1a3n_A
1a3n_C
1a53_A
1a55_A
1a59_A
1a7d_A
1a7v_B
1a7w_A
1a8l_A
1a8l_A
1aa0_A
1aaw_A
1adj_A
1ado_A
1adw_A
1aee_A
1af6_C
1af7_A
1ag6_A
1aih_A
1ajo_B
1akd_A
1aku_A
1aky_A
1an1_E
1aod_A
1aox_A
1apz_D
1aqb_A
1aqy_B
1ar7_4
1asz_B
1au1_B
1au1_B
1aus_L
1aux_B
1avx_A
1aw3_A
1axk_B
1ay8_A
1aym_1
1ayo_B
1b02_A
1b0u_A
1b1y_A
1b21_C

1b2l_A
1b4k_A
1b4q_A
1b54_A
1b63_A
1b76_B
1b87_A
1b8g_B
1b8o_A
1b9h_A
1b9n_A
1b9n_A
1bcp_E
1bcs_A
1bd4_D
1bdo_A
1bem_A
1bf2_A
1bgv_A
1bi9_A
1bif_A
1bin_A
1blx_A
1bmt_B
1boo_A
1bou_D
1bou_D
1bow_A
1bp3_A
1bpe_A
1bpw_A
1bq6_A
1bq8_A
1brd_A
1brk_A
1brw_A
1bsv_A
1bsz_A
1bt4_A
1btg_B
1btn_A
1btw_A
1bvc_A
1bvy_F
1bw9_A
1bxm_A
1bzm_A
1c0a_A
1c0i_A
1c14_B
1c1m_A
1c1x_A
1c39_A
1c3o_B

1c3s_A
1c3x_B
1c47_A
1c51_K
1c52_A
1c7g_B
1c7i_A
1c7n_H
1c8n_C
1c9k_B
1c9q_A
1c9s_K
1c9s_Q
1cah_A
1cbf_A
1cbl_A
1cc5_A
1cch_A
1ccp_A
1cde_D
1ces_B
1cg5_A
1cg5_B
1chn_A
1ci0_A
1ci6_A
1cim_A
1civ_A
1cja_A
1cjt_C
1cjx_C
1ck1_A
1ckl_A
1ckl_B
1ckl_F
1ckm_A
1cko_A
1cl1_B
1cl4_A
1cle_B
1cls_D
1cnj_A
1cnz_B
1co4_A
1coj_A
1coz_A
1cpm_A
1cpt_A
1cr1_A
1cs3_A
1ctt_A
1cur_A
1cvj_A
1cvl_A

1cwu_A
1cx2_B
1cxy_A
1cyx_A
1cyy_A
1d03_A
1d06_A
1d09_B
1d0c_B
1d0l_A
1d0y_A
1d1g_B
1d2f_B
1d2j_A
1d2n_A
1d3y_B
1d5l_C
1d5n_B
1d7p_M
1d8d_A
1d8h_A
1d8u_A
1dad_A
1dah_A
1dap_B
1dar_A
1dbt_A
1dce_D
1dcp_C
1dcp_H
1dcq_A
1ddo_C
1ddo_H
1de0_A
1dek_B
1del_A
1dfg_B
1dfl_X
1dgd_A
1dgm_A
1dhj_B
1dhy_A
1dih_A
1dir_B
1dj3_B
1dj9_A
1dje_A
1dkp_A
1dl2_A
1dl5_A
1dl6_A
1dlj_A
1dlq_A
1dlr_A

1dlw_A
1dly_A
1dm1_A
1dnp_A
1doi_A
1dpi_A
1dq3_A
1dq8_A
1dqi_B
1dqi_C
1dqn_B
1dr2_A
1dtx_A
1dub_A
1dug_B
1dv2_A
1dv6_T
1dva_H
1dvg_B
1dw0_C
1dx5_L
1dx8_A
1dxl_C
1dxm_A
1dxq_D
1dxr_M
1dyq_A
1e05_I
1e08_D
1e0j_E
1e0o_C
1e0o_D
1e12_A
1e2a_C
1e34_B
1e3a_B
1e3c_P
1e3s_A
1e3w_B
1e42_A
1e55_A
1e5q_C
1e5s_A
1e5z_A
1e6e_A
1e6u_A
1e6x_M
1e73_M
1e7l_B
1e7m_A
1e87_A
1e8c_B
1e8e_A
1e8u_B

1e8w_A
1e9l_A
1e9x_A
1eab_A
1eam_A
1eb0_A
1eb6_A
1ebf_A
1ebh_B
1ebo_A
1ebo_F
1ec9_B
1ecj_C
1ecq_A
1ee6_A
1ee8_A
1ee9_A
1ef0_A
1egg_B
1egm_A
1eh6_A
1eh7_A
1eir_A
1ej0_A
1ej6_C
1ekf_B
1elr_A
1em9_A
1en4_A
1ena_A
1eoi_A
1ep3_A
1eqh_A
1eqr_A
1eqr_C
1esm_C
1esn_A
1esr_A
1et4_B
1eu3_A
1eua_B
1euc_B
1eue_A
1ev6_D
1ev9_D
1evz_A
1ewc_A
1ewh_B
1ewh_C
1ex0_B
1exf_A
1ext_A
1exz_A
1eye_A

1eyy_D
1ez0_A
1ezj_A
1ezl_A
1ezw_A
1ezw_A
1ezz_B
1f0x_B
1f17_A
1f1x_B
1f28_B
1f2n_C
1f37_B
1f56_B
1f7d_A
1f7p_A
1f7u_A
1f8v_A
1f97_A
1f99_A
1f9i_A
1f9x_A
1fao_A
1fb1_D
1fbm_B
1fbx_E
1fbx_K
1fc4_B
1fc5_A
1fcd_B
1fd8_A
1fdr_A
1fdv_A
1fe1_G
1fe1_R
1fea_A
1fec_A
1ff1_A
1ffu_F
1fgm_A
1fgx_B
1fgy_A
1fhv_A
1fi2_A
1fid_A
1fk4_A
1fkq_A
1flv_A
1fmc_A
1fn9_A
1fnt_a
1fnt_G
1fob_A
1fp4_D

1fqt_A
1fr9_A
1fsu_A
1ft9_A
1fth_B
1fui_F
1fw2_A
1fwk_A
1fx4_A
1fxa_B
1fxf_A
1fxw_A
1fz5_C
1fz6_C
1fzd_E
1fze_F
1g0v_A
1g2a_A
1g3m_A
1g3r_A
1g41_A
1g4p_A
1g55_A
1g60_A
1g63_B
1g63_F
1g6o_B
1g6t_A
1g71_B
1g73_B
1g8k_F
1g9s_A
1ga1_A
1gc5_A
1gc6_A
1gcg_A
1gcg_A
1gcv_B
1ggf_D
1gh0_O
1ghe_B
1ghq_B
1gi5_A
1git_A
1gjv_A
1gjw_A
1gkl_B
1gks_A
1gl4_A
1gl6_B
1glc_F
1glp_B
1gm8_B
1gmi_A

1gn1_F
1gos_A
1got_G
1goy_A
1gp2_A
1gp4_A
1gp7_A
1gpe_A
1gpj_A
1gqa_A
1gqc_A
1gqi_B
1gqk_A
1gqt_B
1gqy_B
1gqy_B
1grt_A
1grx_A
1gtn_N
1gtq_B
1gtt_A
1gui_A
1guo_B
1gut_D
1guy_A
1gv4_A
1gvf_B
1gvf_B
1gvk_B
1gxb_B
1gxo_A
1gxr_A
1gxu_A
1gy2_B
1gzf_B
1h04_P
1h0a_A
1h18_A
1h1c_D
1h1m_B
1h1p_A
1h1x_A
1h24_C
1h26_A
1h28_A
1h2m_A
1h2u_X
1h30_A
1h31_F
1h3e_A
1h3i_B
1h41_B
1h43_A
1h56_A

1h5n_C
1h5q_H
1h6d_A
1h6g_A
1h6g_A
1h6x_A
1h70_A
1h72_C
1h7f_B
1h7h_A
1h7r_A
1h7u_A
1h9a_A
1h9u_B
1hbo_F
1hby_A
1hcu_A
1hcu_B
1hcz_A
1hda_C
1hdb_C
1he1_A
1hf3_A
1hfb_C
1hg5_A
1hgx_A
1hi1_B
1hi5_A
1hlm_A
1hlq_A
1hlq_C
1hmd_A
1hmw_A
1hmy_A
1ho4_D
1hp0_B
1hpi_A
1hpl_A
1hr6_D
1hr8_F
1hr9_H
1hrs_A
1hsk_A
1hsl_A
1hth_A
1huv_A
1hvy_D
1hw7_A
1hxd_B
1hxi_A
1hxr_B
1hyg_A
1hyh_A
1hyh_C

1hzl_A
1i18_B
1i19_A
1i1h_A
1i1n_A
1i1q_A
1i22_A
1i22_B
1i27_A
1i2k_A
1i36_A
1i43_K
1i4h_A
1i4h_B
1i4y_F
1i4y_H
1i53_A
1i58_A
1i5e_B
1i5g_A
1i5l_C
1i7a_D
1i7l_A
1i7q_D
1i7w_C
1i83_B
1i8l_A
1i95_G
1i96_K
1i96_L
1i97_N
1i97_P
1i9b_D
1i9c_B
1i9g_A
1i9j_H
1i9t_A
1i9z_A
1ia0_A
1ia9_B
1ib1_H
1ibv_D
1iby_B
1ibz_B
1icl_A
1icm_A
1id0_A
1id2_C
1ie0_A
1ie9_A
1ifa_A
1ig0_B
1ig3_A
1iih_B

1iin_A
1iit_A
1ijf_B
1iji_A
1iki_A
1im5_A
1ini_A
1io3_A
1ioz_A
1ipe_A
1iq3_A
1iqp_B
1iqx_A
1iru_A
1iru_D
1iru_D
1iru_K
1iru_O
1iru_Q
1is2_A
1is7_O
1is8_P
1isj_A
1isu_A
1ith_A
1iu9_A
1iuv_A
1ivu_A
1ixe_C
1ixx_E
1iy8_B
1iyb_B
1iyn_A
1iyz_A
1ize_A
1izl_R
1j0a_C
1j0e_A
1j0h_A
1j11_A
1j1t_A
1j1w_C
1j1y_A
1j24_A
1j3b_A
1j3b_B
1j3g_A
1j3l_A
1j3w_D
1j4g_C
1j5p_A
1j5u_A
1j78_B
1j84_A

1j8e_A
1j9l_B
1jaf_A
1jax_A
1jaz_A
1jbo_A
1jcc_A
1jcc_B
1jcc_C
1jd4_A
1jdf_D
1jdi_C
1jdl_A
1jdt_A
1je1_C
1je5_B
1jer_A
1jf8_A
1jg4_A
1jgc_B
1jgc_C
1jgt_B
1jh8_A
1jhj_A
1jhn_A
1ji2_A
1ji4_E
1ji5_B
1jig_A
1jip_A
1jjc_B
1jju_C
1jk0_A
1jk9_B
1jke_B
1jki_A
1jkn_A
1jlk_A
1jlr_B
1jmj_A
1jms_A
1jn7_A
1jn9_B
1joe_B
1joq_A
1jp8_A
1jpm_A
1jq4_A
1jqg_A
1jr3_C
1jr7_A
1jra_A
1jrl_A
1js2_C

1js9_C
1jt2_A
1jtk_A
1juv_A
1jv3_A
1jv7_A
1jvn_A
1jwa_B
1jwb_D
1jwh_D
1jxm_A
1jy7_P
1jy7_X
1jyl_A
1jzn_C
1jzr_C
1k0b_C
1k0e_A
1k0l_A
1k0n_A
1k0v_A
1k1o_A
1k1x_A
1k2e_A
1k2v_N
1k2y_X
1k39_B
1k3h_A
1k4m_C
1k6x_A
1k6z_A
1k73_D
1k7y_A
1ka0_A
1ka2_A
1kbz_A
1kcg_C
1kdi_A
1kdk_A
1kdp_A
1kdt_A
1ker_A
1kfd_A
1kfq_A
1kfq_A
1kh3_D
1kht_C
1kil_A
1kjy_B
1kkj_A
1kkl_A
1kkm_B
1kkt_B
1kl7_A

1klf_D
1klf_H
1km8_A
1kms_A
1kn1_B
1knr_A
1knw_A
1ko8_A
1kob_A
1koq_B
1kp4_A
1kpi_A
1kqc_C
1kqk_A
1kqm_C
1kqo_D
1kqo_F
1kqs_X
1kru_B
1kru_C
1krv_C
1ksk_A
1kuv_A
1kv3_A
1kvj_A
1kvw_A
1kvx_A
1kwh_A
1kws_A
1kxg_F
1kxq_B
1kyq_C
1l1e_A
1l1o_C
1l2e_A
1l3i_B
1l3p_A
1l3p_A
1l5p_A
1l5y_B
1l6m_B
1l7m_B
1l8o_A
1l9b_C
1l9m_B
1laf_E
1lag_E
1laj_B
1lba_A
1lbu_A
1lc7_A
1le6_C
1lfy_B
1lfz_A

1lgh_H
1lgv_B
1lhs_A
1lir_A
1lk9_B
1lka_A
1llc_A
1lml_A
1lo9_A
1loa_F
1loj_C
1loj_D
1loj_G
1loj_M
1lpc_A
1lqa_B
1lqk_B
1lrh_A
1ls9_A
1lsh_A
1lss_C
1lta_E
1ltl_E
1ltv_A
1lu0_A
1luc_B
1lv3_A
1lvu_D
1lvy_A
1lwh_B
1lwu_J
1lxy_B
1m1b_A
1m1j_B
1m1k_4
1m1k_L
1m1n_C
1m1o_A
1m2g_A
1m2v_B
1m32_B
1m36_A
1m3u_H
1m4g_A
1m5b_C
1m5k_D
1m6e_X
1m6m_A
1m6s_D
1m6s_D
1m6y_A
1m7g_A
1m7p_B
1m7s_C

1m90_4
1m9q_B
1mai_A
1maw_F
1mb3_A
1mbb_A
1mbx_A
1mc0_A
1meh_A
1mep_B
1mg2_C
1mg2_L
1mg3_P
1mg5_A
1mih_B
1miv_B
1mj4_A
1mjl_B
1mjt_B
1mka_A
1mkm_B
1mky_A
1mlo_A
1mmf_E
1mms_A
1mmu_B
1mn7_B
1mn9_C
1mni_B
1mo8_A
1moh_A
1mok_D
1mpo_C
1mpx_B
1mq0_B
1mqv_A
1mr1_C
1mrs_A
1mto_A
1mtw_A
1mum_B
1mv8_B
1mww_C
1mwz_A
1mwz_A
1mxf_A
1my3_A
1mzj_B
1mzu_B
1n0j_B
1n0z_A
1n1i_B
1n1q_A
1n26_A

1n2g_A
1n2l_A
1n2l_A
1n2x_A
1n2z_A
1n3s_E
1n3s_G
1n4r_F
1n5d_A
1n5l_A
1n6c_A
1n77_A
1n77_B
1n7u_A
1n83_A
1n97_B
1n9m_C
1n9x_A
1nbh_C
1nbw_A
1ncr_D
1ncs_A
1nd4_A
1nd6_B
1nda_C
1ne7_A
1nee_A
1nek_D
1nf2_B
1nf6_F
1nf8_A
1ng0_C
1ngk_H
1nh7_A
1nhw_A
1nip_A
1nit_A
1njr_A
1nkq_A
1nkq_A
1nks_D
1nli_A
1nlm_B
1nlq_A
1nly_A
1nm5_B
1nmb_N
1no3_A
1noi_C
1nox_A
1npg_A
1nqz_A
1nr0_A
1nr9_D

1nrw_A
1nsa_A
1nsm_B
1nst_A
1nt4_B
1ntm_E
1nu2_A
1nue_D
1nuf_A
1nul_A
1nup_A
1nvb_B
1nvm_A
1nvm_B
1nw1_A
1nw3_A
1nw7_A
1nxc_A
1nxj_A
1ny3_A
1ny5_B
1nyk_B
1nyq_B
1nyt_B
1nzd_A
1nzj_A
1nzr_B
1nzx_G
1o0s_A
1o1l_D
1o1v_A
1o2a_A
1o2r_A
1o3h_A
1o3w_A
1o4s_B
1o4t_B
1o4z_B
1o51_A
1o5i_B
1o61_A
1o6s_B
1o6v_A
1o6z_D
1o7l_B
1o8l_A
1o96_A
1o9r_F
1o9r_F
1o9t_B
1oau_H
1oau_J
1oau_N
1obb_A

1ocm_B
1ocy_A
1od2_B
1od5_B
1odj_B
1odz_B
1oe7_B
1oez_Z
1of6_C
1ofc_X
1ofq_B
1ofq_D
1ofr_F
1og3_A
1og6_C
1ogd_D
1ogp_E
1oh4_A
1ohb_A
1ohf_C
1oi0_A
1oi6_B
1oid_A
1oid_A
1oij_B
1oj7_B
1ojt_A
1ojz_A
1ok4_E
1ok4_J
1okg_A
1okg_A
1omo_A
1omr_A
1on9_A
1opo_B
1oqh_A
1oqj_A
1oqq_A
1or4_A
1ord_A
1ore_A
1osn_A
1ot4_A
1ot8_A
1otw_A
1ov8_C
1oxv_D
1oyj_B
1p0n_B
1p0w_A
1p0y_B
1p0z_G
1p1c_B

1p1j_A
1p2i_A
1p39_A
1p3h_A
1p3w_A
1p44_B
1p45_B
1p49_A
1p4d_B
1p4r_B
1p4v_A
1p5j_A
1p6x_A
1p7c_B
1p8l_A
1p9g_A
1p9l_B
1pbj_A
1pfv_A
1pg2_A
1pg5_B
1pg8_A
1phe_A
1pi1_A
1pi6_A
1pie_A
1pij_A
1pjt_B
1pkd_C
1pkw_A
1pl7_C
1pmi_A
1pmj_X
1pmm_B
1pmm_F
1pn0_D
1pn4_D
1pnc_A
1poc_A
1poi_A
1pos_B
1pov_0
1ppt_A
1pq4_B
1pq8_A
1pqw_A
1pr0_C
1ps7_D
1psz_A
1ptv_A
1pwu_A
1pxe_A
1pyh_2
1pyh_J

1pyh_K
1pyh_S
1pyt_C
1pz0_A
1pz1_A
1pz2_B
1pzh_A
1pzs_A
1pzw_A
1q05_A
1q05_B
1q0g_G
1q0s_A
1q19_C
1q1k_A
1q1w_A
1q23_A
1q2l_A
1q33_A
1q3h_B
1q3q_B
1q3s_F
1q43_A
1q4q_D
1q5e_A
1q5y_C
1q68_A
1q6t_B
1q7h_A
1q81_L
1q82_L
1q86_1
1q8j_A
1q8m_A
1q8y_B
1q90_A
1q90_L
1q92_A
1q99_B
1qab_E
1qao_A
1qap_B
1qbq_B
1qci_A
1qgn_E
1qgn_G
1qgw_A
1qh4_D
1qhf_B
1qhh_A
1qhy_A
1qin_A
1qip_B
1qjq_A

1qjs_A
1qk3_B
1qkc_A
1qkj_A
1qkp_A
1ql0_A
1qlu_B
1qn2_C
1qnf_A
1qoz_A
1qre_A
1qs2_A
1qsm_D
1qvn_A
1qwr_A
1qwy_A
1qxs_D
1qy4_B
1qz9_A
1qzr_A
1qzv_H
1qzv_W
1qzw_B
1r1i_A
1r1n_B
1r1n_D
1r1n_F
1r23_B
1r2j_A
1r2m_A
1r44_A
1r4n_B
1r4w_D
1r4x_A
1r5t_A
1r5u_M
1r61_B
1r6o_B
1r6t_A
1r6v_A
1r8l_B
1r8m_E
1r8q_A
1r9l_A
1r9m_B
1r9p_A
1ra0_A
1rao_A
1ray_A
1rcx_H
1rcx_K
1rdf_E
1reo_A
1rf6_D

1rhc_A
1ri1_A
1rib_B
1rif_A
1rj6_B
1rjd_C
1rkq_B
1rkx_B
1rkx_C
1rlj_A
1rlm_B
1rms_A
1ro5_A
1rov_A
1rp0_A
1rp1_A
1rp4_A
1rqe_A
1rqn_A
1rrv_A
1rrv_B
1rte_B
1rtl_A
1rts_B
1rtx_A
1rus_A
1ruw_A
1rx0_B
1rxq_B
1ryi_B
1ryp_S
1ryq_A
1ryq_A
1rzu_B
1s05_A
1s0p_A
1s0p_A
1s0w_C
1s16_A
1s1q_A
1s20_A
1s2c_A
1s2d_B
1s2k_A
1s49_A
1s4c_B
1s4d_F
1s4m_B
1s56_B
1s5g_A
1s5j_A
1s5k_A
1s5l_j
1s68_A

1s6p_A
1s7n_B
1s85_A
1s8c_A
1s99_A
1sb3_A
1sbz_B
1sc6_B
1sdz_A
1sed_C
1seh_A
1sez_A
1sf3_A
1sf5_A
1sf8_G
1sfk_A
1sft_A
1sg4_B
1sgf_A
1sh3_B
1shk_B
1si1_A
1sjg_A
1sk7_A
1smm_A
1so0_C
1so6_A
1sp1_A
1sp8_D
1sp8_D
1sqb_D
1sqf_A
1sql_I
1sqv_E
1srk_A
1srr_C
1ss9_A
1ssq_D
1sst_A
1stp_A
1su0_B
1suo_A
1suv_F
1sve_A
1svi_A
1sx3_C
1sx6_A
1sxj_A
1sxj_C
1sxj_D
1sxj_D
1syo_A
1sz2_A
1sz2_B

1sz3_B
1szj_G
1szz_C
1t09_B
1t0b_A
1t0b_F
1t0b_G
1t0f_A
1t0i_B
1t0o_A
1t0t_W
1t1e_A
1t2f_B
1t2x_A
1t3k_A
1t3q_F
1t47_A
1t4c_B
1t4w_A
1t5d_X
1t5j_A
1t61_F
1t69_A
1t6c_A
1t6w_A
1t6x_A
1t6z_B
1t8h_A
1t90_D
1t96_A
1ta9_B
1tae_D
1taq_A
1td2_B
1tdi_B
1tdj_A
1te2_A
1ted_A
1tgs_Z
1th1_C
1thj_C
1thn_A
1tiy_B
1tj3_A
1tjl_I
1tkc_B
1tke_A
1tmo_A
1tno_B
1tny_L
1toa_B
1tox_A
1tqy_C
1trz_B

1tt4_A
1tu9_A
1tum_A
1tuu_A
1tuv_A
1tvc_A
1tvg_A
1tw2_B
1twi_B
1twy_A
1tx6_C
1txu_A
1ty2_A
1ty8_A
1ty9_B
1tz6_A
1tzf_A
1tzw_A
1u02_A
1u05_B
1u08_A
1u0b_B
1u0j_A
1u10_C
1u1i_A
1u2d_C
1u2w_B
1u2z_C
1u31_B
1u5k_A
1u5k_A
1u5u_B
1u5v_A
1u72_A
1u7h_A
1u7m_A
1u7p_A
1u80_B
1u85_A
1u96_A
1u9k_B
1u9z_A
1ube_A
1ubg_A
1uc3_F
1uc3_G
1uc5_B
1uc9_A
1udh_A
1udv_A
1ue0_A
1ue1_B
1ued_B
1uf3_B

1uf9_C
1ugi_G
1ugw_A
1uha_A
1uis_B
1ujb_A
1ul3_C
1ulb_A
1um0_D
1umy_A
1umy_D
1unf_X
1uo9_A
1uoc_A
1uod_A
1up8_A
1upr_A
1ups_A
1ur0_B
1ur2_A
1ur4_B
1urj_B
1urk_A
1ury_A
1us5_A
1usy_D
1usy_G
1ut0_B
1ut5_B
1utd_M
1utd_O
1uu6_A
1uup_D
1uus_A
1uvi_B
1uvl_C
1uvm_C
1uvq_A
1uw0_A
1uw3_A
1uw5_B
1uw9_K
1uwk_A
1uwy_A
1uy3_A
1uzu_A
1uzw_A
1v1q_B
1v25_B
1v25_B
1v2b_A
1v2b_B
1v2f_A
1v2x_A

1v33_A
1v3c_A
1v4w_B
1v5e_A
1v5r_A
1v5y_B
1v6d_A
1v6v_B
1v9a_B
1v9h_A
1v9q_A
1v9q_A
1v9u_5
1v9x_A
1vak_A
1vbb_4
1vbe_4
1vbg_A
1vbi_A
1vbl_A
1vcf_A
1vch_C
1vcv_A
1vcv_B
1ve3_B
1vef_B
1vem_A
1veq_D
1veq_H
1vev_B
1vey_B
1vf1_A
1vf2_B
1vfd_A
1vfo_B
1vfw_A
1vh8_A
1vhe_A
1vhl_A
1vhn_A
1vif_A
1vjr_A
1vl8_A
1vli_A
1vly_A
1vm6_B
1vp3_A
1vp4_A
1vp8_A
1vq0_B
1vq6_K
1vr0_A
1vrb_D
1vrt_A

1vsf_A
1vsr_A
1vyg_A
1vz0_D
1vz4_D
1vzm_A
1w07_B
1w0c_A
1w1d_A
1w1w_D
1w2l_A
1w2v_A
1w32_A
1w3b_A
1w3h_A
1w3o_A
1w3r_A
1w3t_D
1w49_B
1w58_1
1w5b_B
1w5c_F
1w5e_C
1w5s_B
1w5t_B
1w5t_B
1w6k_A
1w6m_B
1w81_A
1w85_B
1w9d_M
1wa3_D
1wap_D
1wap_R
1wbj_A
1wc8_A
1wc9_A
1wco_N
1wcz_A
1wfx_A
1wii_A
1wir_A
1wkv_B
1wky_A
1wli_B
1wlv_H
1wmb_A
1wms_A
1wnb_D
1wnd_A
1wno_B
1wnt_B
1woq_B
1wov_B

1wp4_D
1wpk_A
1wpw_B
1wq8_A
1wqa_C
1wro_C
1ws7_A
1wse_B
1wst_A
1wtf_B
1wuf_B
1wuo_B
1wuq_A
1wuw_B
1wve_D
1wwd_A
1wwj_A
1wwk_A
1wws_A
1wwz_A
1wy1_A
1wy7_A
1wy9_A
1wyv_B
1wyx_B
1wzc_B
1wzn_C
1x01_A
1x0g_A
1x0g_C
1x0p_C
1x0x_A
1x1a_A
1x1i_A
1x1v_B
1x31_D
1x35_C
1x35_C
1x3c_A
1x3x_A
1x46_A
1x54_A
1x6f_A
1x7h_A
1x7i_B
1x7p_A
1x8d_B
1x8j_B
1x8u_A
1x9i_B
1x9l_A
1xa1_B
1xai_A
1xc3_A

1xcr_B
1xdg_B
1xdi_A
1xe7_B
1xea_A
1xf1_A
1xf6_A
1xfx_E
1xg5_D
1xh0_A
1xhc_A
1xhl_B
1xi3_B
1xim_C
1xjh_A
1xji_A
1xjj_A
1xjs_A
1xk1_A
1xkv_B
1xkw_A
1xl6_A
1xl8_A
1xlm_B
1xlt_I
1xm5_B
1xmk_A
1xmx_A
1xnq_N
1xnr_D
1xof_A
1xog_A
1xpl_C
1xpu_A
1xpu_B
1xqa_A
1xr2_B
1xru_A
1xrx_A
1xs1_E
1xs2_D
1xs5_A
1xsc_A
1xss_A
1xtf_B
1xtg_A
1xtv_C
1xu2_B
1xv2_C
1xv5_A
1xvl_A
1xvu_A
1xvv_A
1xw4_X

1xw6_C
1xwf_B
1xx1_D
1xxa_C
1xxi_E
1xxr_D
1xxx_F
1xye_B
1xye_C
1xz4_C
1xz4_D
1xz5_B
1xzn_A
1y0c_C
1y0j_B
1y0z_B
1y10_C
1y22_B
1y2x_A
1y3i_A
1y3j_A
1y56_B
1y5e_C
1y5y_C
1y60_D
1y6h_A
1y6p_B
1y7b_C
1y7u_B
1y7v_B
1y81_A
1y8a_A
1y8o_B
1y9g_A
1y9q_A
1y9q_A
1yas_A
1yax_D
1yb0_B
1yb5_A
1ybu_D
1yci_A
1ydy_A
1yfd_B
1ygb_A
1ygp_A
1yit_Y
1yj9_2
1yjn_3
1ykp_A
1yle_A
1yn8_C
1yni_B
1ynq_B

1yo7_A
1yob_A
1yp3_C
1yq2_F
1yq3_D
1yqe_A
1yr3_C
1yra_B
1yre_B
1yrp_A
1ys1_X
1ys4_B
1ys6_B
1ytc_A
1yu2_A
1yun_A
1yvp_B
1yvu_A
1yw0_D
1yw0_D
1yw4_A
1ywd_A
1ywg_Q
1ywq_A
1yxm_B
1z05_A
1z0u_A
1z11_A
1z13_A
1z1s_A
1z25_A
1z2c_B
1z2i_D
1z3h_A
1z3i_X
1z3u_C
1z44_B
1z5g_D
1z5v_A
1z69_D
1z6n_A
1z6o_U
1z6s_B
1z6z_A
1z72_B
1z7e_D
1z8r_A
1za0_A
1za0_A
1za4_A
1zap_A
1zat_A
1zbq_F
1zcw_A

1zcw_A
1zd0_A
1ze1_C
1zei_C
1zem_A
1zfd_A
1zgj_A
1zh1_B
1zh8_A
1zhy_A
1zhz_A
1zjj_B
1zk3_A
1zk7_A
1zke_D
1zl9_B
1zlp_B
1zlq_B
1zlz_B
1zm7_B
1zmi_B
1zmx_E
1zmx_F
1znm_A
1zop_A
1zq1_A
1zq9_B
1zr9_A
1zrr_A
1zs6_B
1zsp_A
1zsw_A
1zsz_C
1zt2_A
1zt2_C
1zt5_A
1zuk_B
1zun_A
1zun_B
1zuq_A
1zuu_A
1zvc_A
1zx5_A
1zx8_C
1zx9_A
1zy2_A
1zy5_A
208l_A
2a06_B
2a0b_A
2a15_A
2a1k_A
2a1t_C
2a1u_A

2a1u_B
2a1y_A
2a2d_A
2a2r_B
2a30_C
2a33_B
2a3l_A
2a4o_A
2a4w_A
2a5f_B
2a5l_B
2a5z_C
2a6r_D
2a7r_A
2a81_A
2a8k_B
2a8y_J
2a98_A
2a9a_A
2a9d_B
2a9f_A
2a9g_D
2a9i_A
2aa6_A
2aac_B
2abj_A
2abk_A
2ac3_A
2acv_A
2adm_B
2aek_B
2afw_B
2afz_B
2ag5_A
2ag9_B
2ahw_A
2ahz_A
2ai7_A
2ai8_A
2aib_A
2aib_B
2aj7_B
2ajh_A
2ajp_A
2ajr_A
2ajr_B
2akf_A
2akl_A
2ako_A
2aky_A
2ale_A
2am4_A
2amc_B
2amt_E

2amu_A
2an3_B
2anx_A
2ao2_B
2ap1_A
2apr_A
2aqc_A
2aqc_A
2arc_B
2arh_A
2ars_A
2as8_A
2au3_A
2auv_A
2av3_B
2avd_A
2avu_F
2aw2_A
2awc_A
2ax4_A
2axk_A
2axt_x
2ay1_A
2ayd_A
2ayj_A
2az1_A
2azd_A
2b06_A
2b25_A
2b30_B
2b3b_C
2b3f_E
2b3j_C
2b3t_A
2b3y_A
2b3y_B
2b3z_B
2b4b_B
2b4l_A
2b4q_A
2b50_B
2b58_A
2b5v_A
2b67_B
2b6h_A
2b7n_B
2b82_A
2b8g_A
2b9d_A
2b9e_A
2b9l_A
2ba9_A
2bb0_B
2bb3_A

2bb6_B
2bb6_C
2bcc_D
2bcn_A
2bco_A
2bcp_B
2bd0_C
2bew_A
2bf0_X
2bff_A
2bgj_A
2bgm_A
2bh1_A
2bhi_A
2bih_A
2bis_A
2bjf_A
2bjr_A
2bk3_A
2bk5_A
2bka_A
2bkx_A
2bla_A
2blf_A
2blo_A
2bm9_F
2bo7_D
2bo7_H
2bo7_J
2bo8_J
2boa_A
2boa_B
2bon_A
2bpc_A
2bq1_F
2bqz_E
2br3_D
2br3_F
2brq_A
2brz_A
2bs4_A
2bs9_H
2bsy_A
2bt1_A
2btq_A
2buf_A
2bvh_D
2bwc_B
2bxr_A
2by9_X
2byc_A
2byl_C
2bz0_B
2bzc_A

2bzd_A
2bzn_G
2c0c_A
2c13_B
2c1l_B
2c2a_A
2c2g_A
2c36_A
2c39_T
2c3h_D
2c4m_B
2c4n_A
2c54_A
2c5c_C
2c5d_D
2c5s_A
2c60_A
2c6b_A
2c6z_A
2c71_A
2c8m_B
2c91_J
2c96_A
2c9h_A
2c9w_B
2c9x_B
2ca3_B
2ca4_A
2ca8_A
2cab_A
2cbu_B
2cc0_A
2ccv_A
2ccy_B
2cf7_D
2cfc_A
2cfo_A
2cg4_B
2cgz_A
2ci6_A
2cin_A
2cir_A
2cj3_B
2cj9_B
2cjz_A
2ckq_A
2ckq_B
2clp_D
2cnb_D
2cns_A
2cqz_A
2cqz_D
2cqz_E
2cr8_A

2crw_A
2cs2_A
2cs7_B
2ct5_A
2ctb_A
2ctu_A
2ctz_A
2cul_A
2cun_B
2cv0_C
2cvj_A
2cwd_B
2cwd_D
2cwf_B
2cww_B
2cx8_A
2cxp_A
2cxx_A
2cy2_A
2cy4_A
2cyc_B
2cye_A
2cz2_A
2cz8_A
2czr_A
2d0b_A
2d0c_A
2d0u_B
2d1c_A
2d1r_A
2d1y_C
2d2c_E
2d36_A
2d3d_A
2d3m_A
2d3m_B
2d3q_A
2d40_C
2d4c_A
2d4e_C
2d4v_B
2d4v_C
2d5a_A
2d5h_C
2d5z_C
2d6f_D
2d73_B
2d8r_A
2d9g_A
2d9l_A
2d9m_A
2dab_B
2das_A
2dbr_E

2dc1_B
2dc3_A
2dcl_A
2dcu_B
2dd4_C
2ddd_A
2ddx_A
2de3_B
2de5_E
2de7_E
2deb_A
2deg_B
2deg_B
2dei_A
2df3_A
2dge_C
2dgk_C
2dgk_D
2dgm_A
2dgm_F
2dh3_A
2dh4_A
2dhr_A
2di3_A
2djr_A
2djw_B
2djw_G
2dki_A
2dkn_A
2dlc_X
2dld_B
2dm6_B
2dob_A
2dpm_A
2dqb_A
2dr1_A
2dr3_D
2dre_C
2drr_A
2drs_A
2ds6_B
2dt5_B
2dt8_A
2dte_B
2dtt_B
2du2_C
2du8_G
2dub_B
2dur_A
2dvh_A
2dvm_A
2dvn_B
2dvz_A
2dw6_D

2dwc_B
2dxb_C
2dxf_A
2dxx_A
2dy0_A
2dy3_D
2e0n_B
2e1b_A
2e1m_A
2e1r_A
2e2e_A
2e2o_A
2e2z_A
2e4u_B
2e5a_A
2e5m_B
2e5v_B
2e5w_A
2e5y_B
2e61_A
2e67_A
2e67_C
2e67_F
2e6c_D
2e6i_A
2e6i_A
2e6v_E
2e72_A
2e7i_A
2e7p_C
2e7r_A
2e7z_A
2e89_A
2e8h_A
2e8v_A
2e8y_B
2e9h_A
2ea7_A
2eab_A
2eb6_A
2eb6_A
2ebr_A
2ebv_A
2ef0_A
2efj_A
2efn_A
2eft_A
2efu_A
2eg3_B
2eg4_A
2egw_A
2egy_C
2eh6_B
2eih_A

2eih_B
2eih_B
2ein_D
2ein_L
2ej9_A
2ejf_C
2ejq_B
2eju_A
2ekl_A
2elm_A
2eln_A
2elq_A
2elv_A
2ena_A
2eni_A
2eo4_A
2eo5_A
2eoi_A
2eou_A
2eoy_A
2epp_A
2eqe_A
2eqf_A
2eqg_A
2erv_A
2erv_A
2esh_A
2esr_B
2etv_A
2eu0_B
2euf_A
2euf_A
2eug_A
2ew2_A
2ewm_B
2ews_A
2exu_A
2exx_A
2ey4_E
2f02_A
2f06_A
2f17_B
2f18_A
2f1k_B
2f1o_H
2f22_A
2f2a_A
2f2e_B
2f44_A
2f48_A
2f49_A
2f4f_A
2f4i_C
2f59_D

2f5t_X
2f5x_B
2f5z_C
2f5z_J
2f6k_A
2f6m_A
2f6r_A
2f6x_B
2f7t_A
2f8b_B
2f8l_A
2f8q_B
2f96_B
2f9i_B
2f9q_D
2f9u_C
2fc6_A
2fco_B
2fcr_A
2fcu_A
2fcu_B
2fdj_A
2fdr_A
2fdx_A
2fe8_C
2fff_B
2ffq_A
2fgc_A
2fgc_A
2fgh_A
2fgu_A
2fi1_A
2fid_B
2fit_A
2fiw_A
2fjc_B
2fjm_B
2fk6_A
2fkb_A
2fkn_B
2fl5_D
2fmx_A
2fn8_A
2fni_A
2fo0_A
2for_A
2fpc_A
2fpg_B
2fqd_A
2fqx_A
2fr6_A
2fr6_D
2fre_B
2frf_A

2frh_B
2fs5_A
2ft0_B
2ftl_E
2ftm_B
2fug_Z
2fut_A
2fv2_A
2fvv_A
2fwe_A
2fyf_A
2fyr_A
2fyu_D
2fzj_A
2fzv_A
2g06_A
2g09_A
2g09_A
2g0d_A
2g0i_A
2g0i_B
2g0i_B
2g0w_B
2g18_E
2g1l_A
2g1u_A
2g25_A
2g37_B
2g38_B
2g3f_A
2g3y_A
2g42_B
2g43_A
2g54_A
2g54_A
2g5f_D
2g5g_X
2g5w_B
2g64_A
2g78_A
2g7f_A
2g80_A
2g8c_O
2g8s_A
2g8s_A
2g8y_A
2g9h_D
2ga4_A
2ga7_A
2gag_A
2gag_D
2gb0_B
2gb2_A
2gb5_A

2gc4_K
2gce_C
2gce_D
2gd2_C
2gd4_B
2gds_B
2gdv_A
2gdz_A
2ge3_A
2gej_A
2gf4_A
2gf6_A
2gfq_A
2ggd_A
2gge_D
2ggh_D
2ggh_D
2ggq_A
2ggs_A
2gh5_B
2gj3_A
2gjv_C
2gjv_E
2gk2_B
2gl5_A
2glu_B
2gm3_E
2gmp_B
2gms_A
2gn2_A
2gnf_A
2gnj_A
2gnv_A
2go7_C
2gp7_A
2gp7_A
2gpe_D
2gq8_A
2gqf_A
2gqk_A
2gqu_A
2gqx_B
2gru_A
2grx_A
2gs9_A
2gsl_F
2gsy_G
2gtl_N
2gtl_N
2gtl_O
2gu3_A
2gvc_E
2gwh_A
2gwl_A

2gyp_A
2h0b_C
2h0b_D
2h0r_E
2h18_A
2h1f_A
2h1l_Q
2h28_A
2h2a_B
2h2u_B
2h32_A
2h32_A
2h3h_A
2h43_F
2h47_C
2h4x_A
2h52_A
2h5m_A
2h5n_A
2h5y_B
2h6l_A
2h79_A
2h7c_D
2h7d_B
2h7s_A
2h92_C
2ha2_A
2ha8_A
2ha8_B
2hae_D
2hbs_H
2hcb_D
2hcr_B
2hek_B
2hes_X
2het_A
2hf1_A
2hfk_A
2hfo_A
2hfu_A
2hh7_A
2hhe_B
2hhe_C
2hi4_A
2hix_A
2hiy_C
2hjn_A
2hk9_B
2hkj_A
2hkv_A
2hkz_A
2hld_V
2hms_B
2hnk_B

2hnl_A
2ho4_B
2hp2_B
2hq9_A
2hqy_A
2hrn_A
2hs6_A
2hsd_D
2hsi_B
2hti_A
2hty_D
2hue_A
2huh_A
2hv6_A
2hv7_B
2hvy_A
2hw1_A
2hw5_A
2hw5_D
2hwu_A
2hwu_D
2hx0_A
2hx1_A
2hxd_A
2hxg_B
2hxs_A
2hyd_B
2hyi_C
2hyj_A
2hzp_A
2i02_A
2i0z_A
2i2a_A
2i2o_A
2i2o_B
2i2r_L
2i2x_C
2i33_A
2i34_A
2i3d_A
2i3g_A
2i3h_B
2i47_A
2i51_B
2i52_F
2i55_A
2i5j_B
2i5m_X
2i5o_A
2i5o_A
2i5r_B
2i5u_A
2i61_A
2i65_B

2i6q_A
2i71_B
2i76_A
2i79_B
2i7a_A
2i7f_A
2i7f_B
2i7h_B
2i7p_B
2i96_A
2i99_A
2i9p_B
2ia5_H
2iag_A
2ibd_B
2ibm_A
2ibp_B
2ici_A
2ick_A
2icp_A
2icy_B
2idj_B
2idx_C
2iec_D
2iej_B
2ies_B
2iew_A
2iey_B
2if7_D
2ifa_B
2ifa_C
2ifc_C
2ig3_A
2ig6_A
2igt_C
2ihu_A
2ihu_A
2ihu_B
2ii5_A
2ii5_D
2ii6_A
2iib_A
2iic_D
2ij2_A
2ijd_1
2ijd_1
2ijm_B
2ime_A
2iml_A
2iml_D
2imq_X
2imr_A
2imz_B
2ins_D

2io4_B
2iop_A
2ipo_B
2iqq_B
2iqy_A
2iry_A
2isl_B
2iu9_C
2iun_B
2iuq_B
2iut_B
2iuu_A
2iuu_B
2iv3_C
2iv7_A
2ive_A
2ivf_C
2ivj_A
2ivn_A
2iw0_A
2iw3_B
2iw9_D
2iwa_A
2iwb_A
2iww_A
2ix1_A
2ix6_E
2ix7_C
2ixa_A
2ixi_B
2ixl_C
2iy4_S
2iyv_A
2izg_B
2izj_A
2j0f_A
2j12_B
2j16_B
2j1n_C
2j28_N
2j3u_A
2j41_D
2j4d_B
2j4j_F
2j4k_D
2j4k_E
2j4q_A
2j4r_A
2j4x_A
2j5s_A
2j5t_C
2j5t_H
2j66_A
2j68_A

2j6a_A
2j6l_C
2j6l_D
2j7d_B
2j7h_A
2j7k_A
2j7m_A
2j7o_A
2j9e_B
2j9l_C
2j9n_A
2j9p_A
2j9u_B
2jae_A
2jag_A
2jap_C
2jb3_B
2jb4_A
2jb7_A
2jbs_D
2jcg_A
2jcg_A
2jd2_A
2jd6_L
2jd6_L
2jda_B
2jeb_A
2jeb_I
2jes_I
2jes_M
2jf3_A
2jfy_A
2jg1_A
2jg1_D
2jg7_E
2jgd_A
2jgr_A
2jgz_A
2jh3_A
2jhf_A
2ji5_A
2jis_A
2jiz_N
2jj1_I
2jjb_A
2jjp_A
2jjx_B
2jjz_A
2jjz_C
2jk1_A
2jkg_A
2jkv_B
2jkv_D
2jky_B

2jl1_A
2jlb_A
2jll_A
2jlv_A
2jm3_A
2joc_A
2jq5_A
2jr7_A
2jtg_A
2jxm_A
2jxy_A
2jz8_A
2k0g_A
2k0x_A
2k2d_A
2k4h_A
2k4x_A
2k5c_A
2k5t_A
2k6z_A
2k74_A
2k78_A
2k7r_A
2k8d_A
2kdp_A
2kdx_A
2kfu_A
2kgo_A
2kid_A
2kkd_A
2kkh_A
2kkr_A
2kkr_A
2kkt_A
2klh_B
2kmd_A
2kmx_A
2kn9_A
2kn9_A
2kny_A
2kpi_A
2kpn_A
2kq9_A
2kr5_A
2ksy_A
2kt4_B
2kv1_A
2kvg_A
2kvh_A
2kw4_A
2kzy_A
2l2e_A
2l47_A
2l4d_A

2l6l_A
2l6m_A
2l6x_A
2l7p_A
2l8e_A
2l8h_A
2l90_A
2l9z_A
2lau_A
2lcq_A
2lha_A
2liw_A
2ljf_A
2ljs_A
2lk5_A
2lki_A
2lko_A
2ll8_A
2lml_A
2lpy_A
2lqw_A
2lvh_A
2lxw_A
2m0d_A
2m0e_A
2m0p_A
2m3g_B
2m6s_A
2m7a_A
2m7b_A
2m7v_A
2m98_A
2m9w_A
2ma7_A
2mas_C
2mcm_A
2mgj_A
2mhb_A
2mhr_A
2mij_A
2mj9_A
2mje_A
2mkd_A
2mmh_A
2mrf_A
2ms0_C
2muc_A
2mur_A
2mwg_B
2mxv_A
2mxz_A
2myb_A
2n1r_A
2n24_A

2n25_A
2n5h_A
2n5k_A
2n7l_C
2n8y_A
2n9o_A
2na0_A
2na5_A
2nab_A
2nax_A
2nb9_A
2ncd_A
2nlq_D
2nly_A
2nn7_B
2nnl_F
2no0_B
2no6_A
2nog_A
2np9_A
2npk_A
2nqy_A
2nr4_A
2nru_B
2ns1_B
2nsf_A
2nss_A
2ntk_A
2ntl_B
2nun_A
2nuw_A
2nv2_F
2nv2_J
2nvo_A
2nvv_A
2nvv_C
2nwb_A
2nx8_A
2nxp_A
2nxp_H
2nyg_A
2nyu_A
2nzu_G
2o05_B
2o0d_A
2o0j_A
2o14_A
2o18_C
2o2c_B
2o2r_B
2o2s_A
2o2z_C
2o36_A
2o4c_B

2o4v_B
2o4z_A
2o56_H
2o6d_A
2o6h_B
2o74_F
2o7p_A
2o7s_A
2o7u_E
2o8q_A
2o97_B
2o9a_A
2o9a_C
2oaj_A
2oal_B
2oap_2
2ob0_C
2oba_A
2obb_A
2obv_A
2oby_E
2oc0_A
2ocz_A
2od0_A
2od5_A
2oda_A
2odp_A
2oee_B
2oel_B
2oel_B
2ofp_B
2og2_A
2og6_A
2og9_A
2oi0_A
2oi2_A
2oik_D
2oiw_A
2oj5_A
2okc_B
2okj_B
2okv_B
2okv_C
2olc_A
2olg_A
2oly_H
2om6_B
2on5_C
2onf_B
2onm_G
2onm_J
2ons_A
2oo0_A
2oog_B

2ooi_B
2ooy_E
2opc_A
2oq2_C
2ord_A
2ore_F
2orp_A
2oso_A
2otl_3
2ou5_A
2ou6_A
2oui_D
2ow6_A
2owa_A
2owl_B
2owq_B
2ox4_B
2ox4_E
2oxc_B
2oxs_A
2oy0_A
2oy9_B
2oy9_B
2oyn_A
2oys_B
2oyy_A
2oyy_C
2ozb_E
2oze_A
2ozg_A
2p06_B
2p0h_A
2p0m_A
2p0s_A
2p0u_B
2p1n_B
2p1n_E
2p2b_B
2p35_A
2p3n_B
2p3p_B
2p42_A
2p4p_A
2p5v_A
2p5y_A
2p6e_C
2p6s_A
2p6w_A
2p7p_F
2p7v_B
2p88_E
2p88_H
2p8u_A
2p97_A

2p9e_A
2p9u_A
2p9x_A
2pa8_D
2pad_A
2paj_A
2par_B
2pbf_A
2pbl_B
2pbz_C
2pc0_A
2pd3_D
2pd4_B
2pd7_B
2peb_A
2peb_B
2pfr_A
2pg0_A
2pg3_A
2pg5_A
2pg8_C
2ph1_A
2pig_B
2pix_A
2pj4_A
2pj5_B
2pjs_B
2pk3_A
2pke_A
2pkp_A
2plj_A
2plm_A
2pls_G
2plw_A
2pml_X
2pms_D
2pno_J
2po5_B
2po7_A
2pos_A
2pos_A
2pos_D
2pp1_A
2ppq_A
2ppt_A
2ppt_A
2pqj_A
2pqy_A
2pr1_A
2pr1_B
2pr5_A
2pr7_B
2prb_A
2prn_A

2prz_B
2prz_D
2pse_A
2ptc_E
2ptf_B
2pul_A
2puz_A
2pvq_A
2pvx_C
2pwh_B
2px5_B
2pxx_A
2py2_B
2py2_B
2pyb_B
2pyu_A
2pyy_B
2pz0_B
2pz8_A
2pzn_A
2q0f_B
2q14_G
2q1d_X
2q1f_A
2q1s_A
2q2a_A
2q3p_A
2q46_B
2q4y_A
2q5e_A
2q5e_F
2q5l_A
2q5o_A
2q5z_A
2q5z_B
2q6h_A
2q6n_F
2q6o_B
2q6v_A
2q78_F
2q7s_B
2q85_A
2q8q_A
2q9c_A
2qa2_A
2qb6_A
2qcl_A
2qcu_B
2qdf_A
2qdg_B
2qe6_A
2qe9_A
2qe9_A
2qeb_B

2qee_E
2qej_C
2qex_T
2qgi_A
2qgs_A
2qgy_A
2qh1_B
2qha_B
2qhf_A
2qio_A
2qji_P
2qjj_D
2qjk_E
2qjk_N
2qk4_B
2qkm_B
2qkw_B
2ql6_D
2qlt_A
2qlu_A
2qlw_A
2qlw_B
2qmu_C
2qnd_A
2qpm_A
2qpo_C
2qq6_B
2qqc_E
2qqd_E
2qqh_A
2qqp_G
2qry_A
2qry_B
2qry_C
2qsz_A
2qt0_A
2qt7_B
2qtn_A
2qtn_A
2qtr_C
2qtt_A
2qtv_A
2qu8_A
2qvh_A
2qvh_B
2qvn_A
2qw5_A
2qyi_B
2qyq_A
2qzc_A
2r0g_A
2r0m_A
2r11_B
2r13_A

2r16_A
2r16_A
2r25_B
2r2g_A
2r2v_C
2r3b_B
2r42_A
2r49_A
2r5r_A
2r66_A
2r6g_B
2r6h_A
2r79_A
2r7a_A
2r7d_A
2r7k_A
2r8t_A
2r8u_A
2r8v_A
2r8z_J
2r93_C
2r94_D
2r96_A
2r9r_A
2raf_A
2rar_A
2rax_E
2rc5_D
2rcl_A
2rct_A
2rd9_D
2rdx_D
2re9_A
2ref_B
2reg_A
2reh_A
2rfc_A
2rfc_B
2rfg_A
2rg4_A
2rg4_B
2rgo_A
2rh4_B
2rhs_C
2ri1_B
2ri4_B
2riq_A
2rir_E
2rj2_A
2rji_A
2rjo_A
2rk1_A
2rk8_A
2rkb_E

2rkv_A
2rl2_A
2rla_B
2rld_A
2rld_A
2rlt_A
2rmk_A
2rnb_A
2rpr_A
2rpz_A
2rsf_A
2rsq_A
2rsy_B
2rte_D
2rtl_A
2rtp_B
2ruu_A
2rv0_A
2rv1_A
2rv5_A
2sar_A
2taa_C
2tdm_A
2tdt_A
2udp_A
2uu7_D
2uul_I
2uul_O
2uuq_A
2uur_A
2uuu_A
2uvn_A
2uvq_A
2uwh_A
2uwj_F
2ux0_C
2uxh_A
2uy5_A
2uyk_C
2uyq_A
2uyr_X
2uz1_B
2uzb_C
2v0s_A
2v0y_A
2v15_H
2v24_A
2v27_B
2v30_A
2v38_A
2v3a_A
2v3g_A
2v3p_A
2v3t_B

2v54_A
2v55_C
2v55_C
2v5k_B
2v62_A
2v7i_A
2v82_A
2v8l_A
2v9k_A
2vao_A
2vat_B
2vbg_A
2vbq_A
2vbs_A
2vc5_C
2vdj_A
2vdv_E
2vee_G
2vf5_X
2vfc_A
2vfg_B
2vfj_D
2vfk_A
2vfr_A
2vfx_E
2vg1_B
2vg3_C
2vgr_C
2vgz_A
2vh3_A
2vha_A
2vhe_A
2vht_B
2vhw_F
2vhz_A
2vis_C
2vjj_A
2vmb_A
2vmh_A
2vmk_B
2vn2_A
2vn8_A
2vnr_A
2vou_A
2vow_A
2vpt_A
2vpw_G
2vq2_A
2vq5_B
2vqe_M
2vqf_R
2vqx_A
2vrd_A
2vrn_A

2vrs_C
2vs1_A
2vsi_B
2vsn_A
2vso_B
2vsq_A
2vsu_B
2vsu_D
2vt3_B
2vue_B
2vut_G
2vuu_B
2vuu_D
2vuu_L
2vuu_M
2vuv_A
2vv7_B
2vvl_H
2vvp_E
2vx5_A
2vx9_A
2vxj_D
2vxj_F
2vxo_B
2vxt_I
2vy0_A
2vy4_A
2vyc_F
2vze_A
2vzs_B
2vzz_C
2w0c_J
2w0c_L
2w0t_A
2w0u_A
2w1b_A
2w1u_B
2w1w_A
2w2h_A
2w2l_A
2w2v_D
2w31_B
2w37_A
2w3n_A
2w3x_C
2w3z_A
2w44_B
2w46_A
2w47_A
2w4l_E
2w4y_C
2w5b_A
2w5q_A
2w67_B

2w7d_A
2w8r_A
2w8x_B
2w98_B
2wae_A
2wag_A
2waq_N
2wat_D
2wb1_O
2wb4_A
2wbx_A
2wc1_A
2wc4_C
2wc6_A
2wca_A
2wcu_B
2wd4_A
2wd8_D
2wew_A
2wfk_A
2wfk_B
2wfk_D
2wg7_A
2wgc_A
2wgr_A
2wi9_B
2wia_A
2wim_A
2win_B
2win_G
2win_J
2wit_A
2wja_A
2wjj_B
2wju_A
2wk1_A
2wkk_C
2wkx_A
2wkx_A
2wle_C
2wll_B
2wlu_A
2wn2_B
2wn7_A
2wnb_A
2wnh_A
2wnl_C
2wno_A
2wns_B
2wny_A
2wo4_A
2wop_A
2wp4_B
2wpf_B

2wpj_S
2wpx_A
2wqd_A
2wqm_A
2wqy_P
2wr8_A
2wsb_B
2wsi_A
2wss_A
2wta_A
2wtn_A
2wtn_B
2wts_A
2wul_B
2wva_X
2wvz_B
2ww0_A
2ww4_A
2wwr_B
2wwz_C
2wyh_A
2wyo_A
2wzv_B
2x03_B
2x0e_B
2x0i_A
2x0l_A
2x0n_P
2x0r_A
2x0u_A
2x0w_B
2x1a_A
2x3b_A
2x3k_A
2x3l_B
2x3y_A
2x3y_H
2x41_A
2x5c_B
2x5d_D
2x5f_A
2x5z_B
2x63_A
2x68_A
2x6b_A
2x6n_A
2x6n_D
2x75_A
2x7w_A
2x7x_A
2x8c_B
2x8t_A
2x8u_B
2x9o_A

2x9v_A
2x9x_A
2xa9_B
2xb4_A
2xb5_A
2xb6_D
2xbf_A
2xbn_A
2xc0_A
2xc1_A
2xci_A
2xcu_C
2xdd_B
2xdh_A
2xdo_B
2xdq_A
2xec_B
2xf4_A
2xfn_A
2xfv_B
2xgt_B
2xhh_A
2xhk_B
2xhn_A
2xhn_A
2xio_A
2xj6_A
2xj9_B
2xjn_C
2xka_G
2xkk_C
2xko_B
2xkr_A
2xla_C
2xmr_A
2xon_L
2xpz_A
2xq1_J
2xq1_P
2xqr_A
2xqx_A
2xr7_A
2xrh_A
2xri_A
2xrp_F
2xs7_A
2xsc_B
2xsg_A
2xtj_A
2xts_A
2xtz_A
2xtz_B
2xum_A
2xv1_A

2xva_B
2xvm_A
2xvz_A
2xw7_A
2xwb_F
2xwg_B
2xwm_A
2xwm_B
2xxa_A
2xxl_A
2xy4_A
2xy8_A
2xyk_B
2xyr_A
2y0o_A
2y0s_P
2y1g_B
2y31_B
2y3q_E
2y48_A
2y4o_A
2y4q_A
2y4r_A
2y6q_B
2y6q_D
2y7e_B
2yaz_B
2yb5_F
2ybg_D
2yc2_B
2yc4_C
2yce_E
2yce_J
2yck_X
2yd1_A
2ydc_A
2yed_A
2yep_C
2yes_B
2yfr_A
2yfu_A
2yg9_A
2ygm_B
2ygp_A
2ygq_A
2yi9_E
2yim_A
2yiu_E
2yj3_A
2yj4_B
2yjl_A
2yk3_A
2yk6_A
2yku_A

2ymd_G
2ymv_A
2ymw_B
2ynm_C
2yol_A
2yox_A
2yp9_A
2ypt_E
2yqc_A
2yqh_A
2yqp_A
2yqz_B
2yr2_B
2yrk_A
2yrm_A
2yrr_A
2yrs_B
2ysa_A
2yta_A
2ytg_A
2yu2_A
2yu4_A
2yv9_A
2yvl_B
2yw2_B
2ywr_A
2yx6_C
2yxx_A
2yy7_B
2yy8_B
2yyl_A
2yyy_A
2yzb_C
2yzb_H
2yzm_B
2yzq_A
2z02_B
2z0a_D
2z1z_B
2z3g_A
2z3o_A
2z3p_B
2z41_A
2z5i_E
2z5x_A
2z67_D
2z6c_B
2z6f_A
2z6o_A
2z7b_A
2z7c_C
2z7e_B
2z8q_B
2zad_D

2zai_C
2zax_A
2zb4_A
2zbl_B
2zbo_E
2zbw_B
2zcu_A
2zcz_D
2zdp_A
2ze3_A
2ze6_A
2zed_B
2zee_B
2zel_A
2zew_B
2zfu_A
2zgi_A
2zhh_A
2zhz_A
2zi8_A
2zif_B
2zim_A
2ziy_A
2zj0_D
2zj1_B
2zj5_A
2zjp_Y
2zjs_Y
2zk2_A
2zkb_A
2zkl_A
2zkm_X
2zny_A
2znz_D
2zp8_B
2zpl_B
2zpu_A
2zq0_B
2zr2_B
2zrm_A
2zru_C
2zrz_D
2zsg_A
2zsi_A
2zsj_A
2zt9_H
2zti_A
2zu8_A
2zue_A
2zus_D
2zut_A
2zvc_A
2zvl_B
2zw5_B

2zwa_B
2zwv_A
2zxu_A
2zy3_A
2zy4_F
2zyh_A
2zzj_A
2zzj_A
2zzn_A
2zzs_1
2zzs_N
2zzs_X
2zzv_A
3a0u_A
3a11_B
3a15_D
3a1l_A
3a23_A
3a25_A
3a2y_A
3a32_A
3a35_B
3a3y_A
3a43_A
3a4w_A
3a5r_A
3a6q_B
3a6t_A
3a7a_C
3a7q_B
3a7r_A
3a89_A
3a8i_C
3a8r_B
3a8t_A
3a99_A
3a9e_B
3a9f_A
3a9g_A
3a9l_B
3a9v_A
3a9z_A
3aam_A
3ab1_B
3ab4_B
3ab9_A
3abi_A
3abl_S
3abx_A
3ac0_A
3aci_A
3acl_A
3adc_A
3add_A

3ae3_C
3aej_C
3af1_A
3afk_A
3afn_A
3afo_A
3ag1_F
3ag1_S
3agc_A
3agm_A
3agn_A
3agu_A
3ah2_B
3ahg_A
3ahm_A
3ahq_A
3ahr_A
3ai2_G
3ai3_G
3aia_A
3aia_B
3aib_E
3aic_G
3ajm_A
3ajx_D
3ak2_A
3ak4_B
3ak5_B
3ak9_F
3akh_A
3akm_D
3al5_B
3all_B
3alu_C
3am3_A
3amj_C
3amq_A
3amq_C
3anm_B
3ann_A
3anx_B
3ao1_B
3ao2_A
3aoe_B
3ap1_B
3ap2_A
3apt_A
3aq0_G
3aqj_C
3aql_A
3arl_A
3as5_A
3asi_A
3ask_C

3aso_F
3atp_A
3atr_A
3aty_A
3atz_A
3auu_A
3av0_B
3aw8_A
3awd_A
3awj_A
3awo_A
3awq_A
3awu_B
3awx_B
3ax1_A
3ax1_A
3axh_A
3axs_A
3azr_A
3azx_A
3b00_A
3b05_A
3b06_D
3b0a_E
3b0u_X
3b1b_B
3b1c_C
3b1d_A
3b1f_A
3b1n_B
3b1v_A
3b1w_A
3b2e_C
3b3d_C
3b42_B
3b47_A
3b4r_A
3b4u_A
3b4y_A
3b55_A
3b59_E
3b5z_D
3b6m_B
3b6n_A
3b6o_B
3b70_A
3b74_A
3b7p_C
3b7w_A
3b8i_F
3b8t_C
3b9o_A
3b9z_A
3bal_B

3bb6_D
3bb8_B
3bbd_B
3bbx_U
3bcc_D
3bcx_A
3bcx_A
3be6_A
3be7_A
3bem_B
3ber_A
3bf3_A
3bfj_H
3bfk_D
3bg9_A
3bga_A
3bgd_B
3bhf_A
3bhu_D
3bhu_D
3bi2_A
3bi4_A
3bix_B
3bj4_A
3bjd_C
3bjs_B
3bk5_A
3bkh_A
3ble_A
3blw_M
3bm1_B
3bm5_A
3bmq_A
3bn1_A
3bnk_B
3bnx_B
3boc_A
3bof_A
3bom_D
3boy_B
3bp8_B
3bpt_A
3bpw_A
3bpz_C
3bq6_A
3bqb_X
3bqb_Z
3bre_B
3brn_A
3brq_A
3btd_E
3bts_B
3btt_E
3bu1_A

3bud_A
3bui_A
3buj_A
3bus_A
3bus_B
3bvo_A
3bvq_B
3bw4_A
3bwl_A
3bwn_F
3bwv_B
3bx1_C
3bxg_B
3by0_C
3by6_C
3byn_A
3bza_D
3bzj_A
3bzn_A
3c01_E
3c03_A
3c0h_A
3c0o_A
3c1o_A
3c20_B
3c2c_A
3c2w_D
3c2y_A
3c37_A
3c3e_A
3c41_J
3c4a_A
3c4e_C
3c4n_B
3c5n_A
3c6a_A
3c6c_A
3c6h_A
3c6k_D
3c6l_A
3c6m_C
3c7g_A
3c7h_A
3c7j_A
3c7t_C
3c85_C
3c8f_A
3c8l_A
3c8v_C
3c8z_B
3c96_A
3c9f_B
3c9f_B
3c9h_A

3c9h_B
3caw_A
3cb0_A
3cb2_A
3cce_B
3ccl_B
3ccm_Y
3ccr_3
3ccu_T
3cd3_A
3cdw_A
3cdx_A
3cdx_B
3ce6_D
3ce9_D
3cez_A
3cf8_A
3cfo_A
3cg4_A
3cg7_A
3cgh_A
3cgm_A
3cgx_A
3chl_A
3ci0_I
3cia_A
3cia_B
3cj7_A
3cj8_A
3cjc_G
3cji_B
3cjt_E
3cjy_A
3ckg_A
3cko_A
3clt_D
3clv_A
3cm6_A
3cme_C
3cmm_C
3cmq_A
3cng_B
3cnj_A
3cnl_A
3cnx_C
3co4_A
3co8_A
3co8_B
3cp5_A
3cp8_C
3cp8_D
3cq3_C
3crl_B
3crl_B

3crv_A
3csl_A
3csm_B
3csq_D
3csv_A
3csw_C
3csx_A
3ct7_B
3ct7_B
3ct7_C
3ct8_A
3cts_A
3cu3_A
3cuk_A
3cuk_B
3cvg_A
3cvj_A
3cvz_A
3cw8_X
3cwb_W
3cwq_B
3cwy_A
3cx3_B
3cxm_A
3cxq_A
3cz0_A
3czl_A
3czv_A
3czx_C
3czx_D
3d07_A
3d0g_B
3d2d_A
3d2h_A
3d41_A
3d45_A
3d47_F
3d54_I
3d5j_A
3d6w_A
3d82_D
3d87_D
3d89_A
3d8n_A
3d8p_A
3d8v_A
3d9b_A
3d9d_A
3d9f_B
3d9g_D
3da1_A
3da3_A
3da8_A
3dan_A

3dap_A
3dba_A
3dbh_D
3dbl_H
3dbn_A
3dbv_O
3dc2_A
3dc2_B
3dc6_A
3dc7_B
3dcb_A
3dcm_X
3ddd_A
3ddj_A
3ddl_A
3ddv_B
3de1_X
3de2_X
3dee_A
3deo_A
3dff_A
3dfy_D
3dfy_H
3dfy_M
3dg7_D
3dgi_B
3dgt_A
3dgv_B
3dh0_B
3dh1_A
3dh4_C
3dh4_D
3dhr_H
3dhy_C
3di1_B
3di4_A
3di5_A
3dkx_A
3dky_A
3dlc_A
3dm5_A
3dme_B
3dmt_D
3dnd_A
3dnf_B
3dnp_A
3dou_A
3dp5_A
3dqx_B
3dr0_A
3dr1_A
3dr8_B
3drw_A
3dsb_A

3dt5_A
3dtn_A
3dtt_B
3du4_B
3du7_C
3duf_C
3dv0_C
3dv0_D
3dv9_A
3dwb_A
3dwg_A
3dx1_A
3dxj_B
3dxp_A
3dxs_X
3dxy_A
3dyd_A
3dye_A
3dyq_A
3dzc_A
3dze_A
3dzh_B
3dzm_A
3dzo_A
3dzv_A
3e03_A
3e12_A
3e1t_A
3e1y_A
3e1z_A
3e23_A
3e24_B
3e2a_A
3e2i_A
3e2j_C
3e2v_A
3e31_A
3e39_A
3e3g_A
3e3u_A
3e46_A
3e4c_B
3e4d_B
3e4q_A
3e4v_A
3e4w_A
3e5p_C
3e5z_B
3e6q_L
3e6u_A
3e6z_X
3e77_A
3e7f_B
3e7j_A

3e7l_A
3e7v_A
3e7w_A
3e81_B
3e8s_A
3e8x_A
3e9h_A
3e9i_C
3e9q_B
3ea4_A
3ea6_A
3eaf_A
3eau_A
3ebb_B
3ebe_B
3ebe_C
3ec1_B
3ec7_F
3eca_A
3eca_D
3ecr_A
3edo_A
3edy_A
3eef_B
3eey_A
3ef1_A
3efo_B
3efr_B
3eh1_A
3ei8_A
3eib_A
3eif_A
3eih_A
3eii_D
3eip_A
3ejn_A
3ejn_A
3ek3_A
3ekg_B
3el3_A
3elc_A
3elc_C
3ele_B
3ele_D
3ell_A
3eln_A
3els_A
3elu_A
3emm_A
3en8_A
3en9_A
3enz_A
3enz_C
3eo3_A

3eo6_B
3eo8_C
3eob_J
3eof_A
3ep3_A
3ep9_A
3epa_A
3epm_A
3epy_A
3eqe_A
3eqo_A
3eqq_A
3eqq_B
3erg_B
3err_A
3es8_A
3es8_E
3esw_A
3etc_B
3eu3_A
3eu9_A
3eue_A
3euh_A
3euk_H
3euk_H
3evk_B
3ew5_C
3ewd_A
3ewk_A
3exn_A
3exs_D
3ey7_A
3eyx_A
3ez9_A
3ezw_A
3f0d_D
3f0m_A
3f0y_C
3f1j_A
3f1k_A
3f1t_B
3f1y_A
3f2v_A
3f3r_A
3f3s_A
3f4f_A
3f4n_C
3f5f_A
3f5m_C
3f5o_H
3f69_B
3f6h_A
3f6s_A
3f6s_I

3f6u_H
3f6v_A
3f79_E
3f7o_B
3f7p_D
3f7p_D
3f8h_A
3f8k_A
3f92_A
3f9p_B
3f9p_D
3f9t_B
3fa3_B
3fa3_K
3fav_D
3fbu_A
3fbv_C
3fbv_L
3fca_B
3fcm_B
3fcr_A
3fcv_B
3fcy_A
3fd3_A
3fd3_A
3fe4_B
3fef_B
3fes_C
3fes_D
3feu_A
3ff1_B
3ffo_A
3ffr_A
3ffz_A
3fg9_A
3fgc_A
3fh0_A
3fha_A
3fha_A
3fhd_A
3fhl_D
3fig_A
3fii_B
3fij_F
3fj4_A
3fjg_D
3fju_B
3fju_B
3fkh_C
3fki_L
3fki_L
3fl8_A
3fm2_A
3fms_A

3fmx_X
3fna_B
3fnb_A
3fp2_A
3fp5_A
3fp8_E
3fpa_A
3fpj_B
3fpv_H
3fqg_A
3fqm_B
3fqt_A
3fr6_A
3fr9_A
3frx_D
3fs1_A
3fsb_A
3fsn_A
3ftf_A
3fux_C
3fv9_C
3fvd_B
3fvu_B
3fw0_A
3fwf_B
3fwr_B
3fws_B
3fwz_A
3fx6_A
3fxg_G
3fxu_A
3fyb_A
3fyb_A
3fym_A
3fyn_A
3fyu_E
3fz0_B
3fz3_B
3fzg_A
3fzi_A
3fzy_A
3g0h_A
3g0k_A
3g1f_A
3g1z_A
3g22_A
3g2p_A
3g2y_A
3g3r_A
3g5o_A
3g5q_A
3g5t_A
3g6e_B
3g6o_A

3g71_A
3g71_B
3g8b_B
3g8q_A
3g8r_A
3g8r_A
3g93_C
3gag_A
3gal_B
3gaz_A
3gb7_C
3gbh_C
3gbu_B
3gce_A
3gcm_A
3gd3_A
3gdh_A
3gdj_A
3gdp_B
3ge1_D
3ge4_C
3ge5_A
3gee_A
3geg_B
3gei_A
3gfa_B
3gfb_B
3gff_B
3gfr_D
3gfs_A
3gfv_B
3gfy_B
3gg7_A
3ggg_D
3gh7_A
3ghg_I
3ghg_I
3giu_B
3gj9_A
3gj9_A
3gjn_C
3gl9_B
3glq_B
3glv_B
3gma_B
3gmd_H
3gn5_A
3gn6_C
3gnd_B
3gnd_O
3gnd_P
3gni_B
3go7_B
3gob_A

3gob_C
3goe_A
3gon_A
3gor_A
3gor_C
3gos_A
3gq1_A
3gqg_A
3gr3_B
3gr9_C
3gra_A
3grf_A
3grr_A
3gru_A
3gs2_A
3gsh_B
3gta_B
3gtv_D
3gu3_A
3gvd_E
3gvd_I
3gw6_F
3gwc_A
3gwl_A
3gwz_C
3gxa_C
3gxa_F
3gxy_A
3gy3_A
3gyb_A
3gyd_B
3gyg_A
3gyl_B
3gyx_K
3gz2_A
3h08_B
3h0g_B
3h0g_J
3h0g_L
3h0g_N
3h0l_D
3h0r_G
3h1j_N
3h1j_S
3h1q_B
3h2b_B
3h2s_B
3h31_A
3h36_A
3h39_B
3h3e_A
3h3p_H
3h4l_B
3h4s_E

3h4v_E
3h5a_C
3h5c_B
3h6g_B
3h6o_B
3h6o_C
3h70_A
3h77_B
3h7c_X
3h7f_B
3h7f_B
3h7u_A
3h7v_A
3h85_A
3h87_A
3h87_B
3h8l_B
3h8s_A
3h8s_A
3h8t_B
3h97_A
3h9u_A
3hai_A
3hb0_C
3hbg_A
3hbl_A
3hbp_A
3hcj_B
3hdg_A
3hdo_A
3hdp_A
3hf7_A
3hfg_B
3hft_A
3hgi_A
3hgw_D
3hgx_B
3hgy_A
3hgz_A
3hhe_B
3hhi_A
3hhj_B
3hi6_A
3hic_A
3hj1_B
3hj9_A
3hja_A
3hjk_A
3hjt_A
3hkt_A
3hlo_A
3hlp_B
3hly_B
3hlz_B

3hm7_B
3hml_B
3hn1_A
3hn3_E
3hnh_A
3hnm_A
3ho5_A
3ho6_A
3hof_C
3hpa_A
3hpg_B
3hpg_E
3hpv_D
3hpz_B
3hq0_D
3hqd_A
3hqt_B
3hr4_G
3hr6_A
3hrd_G
3hsk_A
3hsv_A
3ht1_A
3hta_A
3htj_A
3htk_C
3htn_A
3hu2_C
3hug_J
3huj_H
3hus_F
3hwr_B
3hx6_A
3hy5_A
3hyp_B
3hyv_C
3hzh_A
3hzl_A
3i0p_A
3i12_A
3i16_B
3i2b_L
3i3l_A
3i3o_B
3i3o_B
3i3t_A
3i3w_A
3i45_A
3i4i_A
3i4k_F
3i4x_A
3i54_D
3i56_C
3i57_B

3i5c_A
3i5h_C
3i5u_B
3i5y_A
3i6d_B
3i6i_A
3i6t_A
3i6t_B
3i6v_A
3i77_A
3i7v_B
3i8b_A
3i8b_A
3i8x_B
3i92_B
3i92_C
3i98_C
3i98_E
3i9v_H
3ia7_A
3ia8_B
3iae_A
3iam_4
3iam_5
3iam_7
3iar_A
3iax_A
3ib5_A
3ib9_A
3ib9_B
3ibm_B
3ibr_A
3ibs_A
3ic3_B
3ic9_C
3ici_A
3ieh_A
3iei_A
3ieu_A
3if2_B
3ig2_C
3ig9_B
3ig9_C
3iga_C
3igs_A
3igy_B
3ihb_A
3ihg_C
3ihj_A
3ihp_B
3ihq_A
3iii_A
3iiv_B
3ij6_A

3ik2_A
3ikh_A
3ikv_A
3ilm_B
3im0_A
3im9_A
3imp_K
3imx_A
3in1_B
3in6_A
3in9_A
3ip4_B
3ip5_A
3ipk_A
3ipr_E
3iqn_A
3iqt_A
3iqz_E
3ir0_N
3ir0_W
3irb_A
3irh_C
3is7_A
3isl_B
3it5_A
3it6_B
3it7_A
3iu6_A
3iup_A
3iuy_A
3iv6_A
3ivs_A
3ivs_B
3ivu_B
3iw4_A
3iwa_A
3iwf_B
3iwt_A
3ixc_A
3iyl_G
3j4k_A
3j4s_A
3j7h_D
3j7y_0
3j7y_E
3j81_a
3j81_k
3j82_A
3j8x_B
3j95_C
3j9d_A
3ja8_3
3ja8_5
3ja8_6

3ja8_7
3jam_J
3jap_l
3jaq_Q
3jar_B
3jas_B
3jb7_B
3jb9_B
3jb9_c
3jb9_X
3jb9_Y
3jbb_E
3jd0_A
3jdw_A
3jq7_D
3jqm_E
3jqp_D
3jsa_A
3jsj_C
3jsk_C
3jt1_A
3jtd_B
3ju2_A
3juc_A
3jus_B
3juv_A
3jva_F
3jvf_B
3jvf_B
3jw4_C
3jwf_B
3jwg_A
3jwh_A
3jyg_C
3jyp_A
3jys_A
3jz6_B
3jzd_D
3k0t_A
3k0t_C
3k15_A
3k15_A
3k1f_M
3k1j_A
3k1m_B
3k1n_B
3k2b_C
3k2b_F
3k2i_A
3k38_H
3k38_K
3k39_I
3k3a_D
3k42_B

3k4b_A
3k4i_C
3k4m_F
3k60_A
3k6h_A
3k6j_A
3k6x_B
3k7r_B
3k7y_A
3k8b_B
3k9u_B
3k9v_A
3k9v_B
3k9x_B
3ka7_A
3kak_A
3kb2_A
3kb6_A
3kbo_D
3kbr_A
3kc2_A
3kcg_H
3kd3_A
3kd6_A
3kdp_C
3kdy_B
3kew_A
3kez_B
3kf3_B
3kgd_B
3kgr_B
3kgw_B
3kgx_A
3kgy_B
3kgz_B
3khb_B
3khi_A
3ki8_A
3kjg_A
3kjh_A
3kkz_A
3klb_A
3klj_A
3klu_A
3kmh_A
3kmt_B
3kmv_E
3kn3_B
3knz_A
3knz_C
3ko1_H
3ko5_C
3kob_B
3koq_A

3kpf_A
3kpt_B
3kpx_A
3kq0_A
3kq4_E
3kqg_A
3kqj_A
3ks6_A
3kst_A
3kt1_A
3kta_A
3ktb_D
3ktn_A
3ktw_B
3ku0_B
3ku1_G
3kui_A
3kvt_A
3kvu_B
3kvy_B
3kw0_C
3kws_A
3kx5_B
3kxa_A
3kyc_B
3kzb_A
3kzh_A
3kzn_A
3kzp_A
3l0a_A
3l0c_B
3l0f_A
3l12_A
3l1e_A
3l21_B
3l23_A
3l2n_A
3l31_A
3l3t_D
3l4g_C
3l4n_A
3l6h_A
3l6r_A
3l74_G
3l7t_B
3l7x_A
3l7y_A
3l8a_A
3l8e_A
3l8k_A
3l9b_A
3l9f_B
3l9l_B
3la2_B

3lag_B
3lb3_A
3lb6_D
3lbc_B
3lbf_C
3lc6_B
3lcc_A
3lcn_B
3lcu_A
3ld0_a
3ld0_j
3ld2_B
3ld8_A
3ldu_A
3ldv_A
3lec_A
3lee_D
3lf2_C
3lf5_B
3lfi_B
3lft_A
3lgg_A
3lgh_C
3lgj_A
3lgx_D
3lhh_A
3lho_A
3lhr_A
3lhs_A
3lie_A
3lix_A
3lju_X
3lkb_B
3lkf_A
3lki_B
3lkk_B
3lku_A
3ll3_A
3ll4_B
3ll9_B
3llx_A
3llx_A
3lm4_A
3lm4_D
3lm4_D
3lm5_A
3lm8_C
3lmg_A
3lnb_A
3lnc_A
3lnn_A
3lnv_A
3loc_C
3loo_B

3loq_A
3lor_B
3lot_B
3lov_A
3lp9_A
3lpp_B
3lq0_A
3lqf_D
3lqv_B
3lrt_B
3lsj_A
3lst_A
3lsz_A
3lt1_A
3ltv_A
3ltv_F
3luj_B
3luu_A
3lvw_A
3lw7_B
3lxy_A
3ly1_D
3lyg_A
3lyr_A
3lzd_A
3lze_A
3lzk_C
3lzo_B
3lzz_B
3m0f_A
3m0i_A
3m12_B
3m1c_A
3m1m_A
3m1n_B
3m1y_A
3m2p_B
3m2r_B
3m2t_B
3m36_A
3m4g_I
3m4r_A
3m4w_F
3m5k_A
3m6m_D
3m6m_E
3m6v_A
3m7n_A
3m83_F
3m84_A
3mam_A
3mb5_A
3mbh_A
3mdd_B

3mdl_B
3mds_A
3mdw_D
3mdz_A
3me3_D
3meb_A
3men_A
3mff_D
3mfq_A
3mfu_A
3mfu_A
3mg2_A
3mg5_D
3mg6_H
3mg6_K
3mga_B
3mgc_A
3mgd_A
3mgx_B
3mhh_C
3mhh_E
3mhm_A
3mi9_A
3mj5_A
3mjh_B
3mjv_A
3mk7_L
3mmz_D
3mo4_A
3mp2_A
3mp3_E
3mq2_A
3mq7_E
3mqh_D
3mqt_D
3mqt_F
3mqt_V
3msz_B
3mte_B
3mv4_M
3mvc_A
3mwc_A
3mwe_A
3mwf_A
3mwp_B
3mwp_C
3mx3_A
3myu_B
3mz8_A
3mz9_A
3n0a_A
3n0w_A
3n0y_A
3n0y_A

3n1c_A
3n1f_C
3n1o_B
3n1s_J
3n26_A
3n29_A
3n2i_B
3n2n_F
3n3a_D
3n3k_A
3n4k_A
3n5n_Y
3n5o_A
3n60_B
3n6h_B
3n6h_D
3n6l_A
3n6q_H
3n7u_C
3n8u_B
3n8z_A
3n91_A
3n9b_B
3n9i_B
3n9k_A
3n9r_P
3na1_A
3na5_B
3na8_A
3nat_A
3nba_C
3nba_D
3nbs_A
3nbx_X
3nc6_B
3nd1_B
3ndc_A
3nel_A
3nfd_E
3nfm_A
3ng7_X
3ngf_B
3ngl_A
3ngt_F
3nh8_A
3nh9_A
3nhq_H
3nix_A
3njh_D
3njr_A
3nk7_B
3nks_A
3nl1_A
3nl5_A

3nl5_A
3nll_A
3nm7_A
3nmi_A
3nmj_D
3nne_C
3nng_A
3nng_B
3nnl_B
3nns_B
3no1_C
3no4_A
3no4_B
3no6_A
3nok_A
3nop_C
3nou_C
3noy_B
3nqn_A
3nr1_B
3nrb_D
3nre_D
3nrj_F
3nrn_A
3nrt_A
3ns1_C
3ntn_A
3nug_B
3nut_C
3nuv_A
3nvn_B
3nwb_A
3nwe_A
3nwo_A
3nwz_C
3nxl_B
3nxl_C
3nxs_A
3nxx_A
3nye_A
3nyi_B
3nyo_B
3nzp_A
3nzt_A
3nzz_A
3o01_B
3o0h_B
3o14_B
3o14_B
3o15_A
3o2e_A
3o3c_A
3o3m_D
3o3n_C

3o3p_B
3o55_A
3o5b_A
3o6d_A
3o8c_A
3o90_B
3oa2_B
3oa4_A
3oa8_B
3oa8_D
3oa8_E
3oaj_B
3oba_D
3obk_A
3obk_B
3oc4_A
3oca_B
3ocd_C
3ocy_A
3ocz_A
3oee_D
3oeh_B
3oeh_C
3oet_B
3oev_K
3of2_A
3of4_A
3ofk_A
3ofn_A
3ofn_M
3ogr_A
3oh2_A
3oh4_A
3ohi_A
3ohs_X
3oi7_A
3oi7_B
3oib_A
3oid_B
3oif_A
3oij_B
3ois_A
3oix_C
3oiy_A
3oj6_B
3ojl_A
3ojo_A
3oka_A
3okk_A
3okp_A
3oks_C
3okx_A
3ol5_A
3olb_I

3om7_D
3omb_A
3ome_D
3omy_B
3onn_A
3onr_A
3oo6_A
3oo6_B
3opk_C
3opy_D
3oq3_A
3orf_C
3orh_A
3orj_A
3ork_A
3os4_A
3os4_A
3oss_D
3oth_A
3oti_A
3otk_A
3ou7_A
3oum_A
3out_C
3ovs_B
3ovw_A
3ow2_K
3owa_C
3owc_A
3owv_B
3ox6_E
3oxh_A
3oxo_F
3oy7_B
3oyr_A
3oz2_A
3ozb_A
3ozm_A
3p0z_A
3p13_A
3p1m_H
3p24_A
3p27_B
3p2a_A
3p2h_A
3p2n_A
3p3c_A
3p3o_A
3p47_A
3p5n_B
3p70_H
3p7f_D
3p7i_A
3p7n_A

3p8v_A
3p9c_A
3p9l_D
3p9l_E
3p9y_B
3paj_B
3paq_A
3pbm_B
3pc4_A
3pc8_B
3pcb_M
3pcb_N
3pcc_M
3pce_R
3pch_M
3pch_O
3pch_R
3pcj_O
3pck_P
3pdu_E
3pe0_B
3ped_A
3pef_D
3peg_A
3pf3_A
3pfd_D
3pfp_A
3pfr_D
3pfs_A
3pft_A
3pft_B
3pgv_A
3ph7_B
3phi_B
3pif_C
3pij_B
3pio_C
3pip_U
3piv_B
3pje_B
3pk0_C
3pk7_A
3pkw_A
3pl1_A
3plq_A
3pls_A
3pm0_A
3pm6_A
3pm9_F
3pmm_A
3pn7_F
3pnd_C
3pns_E
3poa_A

3poa_A
3poh_A
3poy_A
3pp9_A
3ppv_A
3ppz_B
3pqc_B
3pqi_A
3pr3_A
3pr8_C
3psh_A
3ptb_A
3ptz_C
3pvh_A
3pvt_A
3pvz_A
3pw1_A
3pw3_A
3pwx_A
3pxp_A
3pxv_B
3pxx_A
3pym_A
3pyz_A
3pzc_A
3pzi_A
3q01_A
3q03_B
3q13_A
3q1o_A
3q2k_C
3q2k_N
3q2m_A
3q2o_B
3q2r_A
3q31_A
3q33_A
3q34_A
3q34_B
3q3g_G
3q3h_A
3q4o_A
3q6d_B
3q6i_A
3q6k_B
3q7h_A
3q7j_A
3q80_A
3q87_A
3q87_B
3q8i_A
3q8j_A
3q8x_D
3q9c_K

3q9e_G
3q9f_B
3q9o_A
3qaw_A
3qax_B
3qay_C
3qb5_K
3qbd_A
3qbm_A
3qbw_B
3qc6_X
3qcp_A
3qd2_B
3qdh_A
3qdk_A
3qdl_A
3qdq_A
3qed_A
3qee_B
3qf7_A
3qfe_A
3qff_A
3qff_B
3qfy_A
3qi9_A
3qib_B
3qix_A
3qj0_A
3qj4_A
3qjg_E
3qke_H
3qkg_A
3qku_A
3qku_B
3qlb_A
3qm2_B
3qm3_E
3qml_C
3qn0_E
3qn9_A
3qnm_A
3qns_A
3qqp_C
3qr0_A
3qsi_D
3qsj_A
3qsy_A
3qsz_B
3qt1_I
3qu8_C
3qui_A
3quo_A
3qv1_A
3qv1_F

3qv2_A
3qvh_A
3qvm_B
3qw2_C
3qw8_A
3qwb_C
3qwb_D
3qwu_A
3qx9_B
3qxe_C
3qxs_A
3qxy_A
3qyf_A
3qyy_B
3qz1_A
3qz6_A
3qzm_A
3qzp_B
3r03_A
3r09_A
3r0n_A
3r0q_G
3r10_A
3r11_B
3r18_A
3r1a_B
3r1i_A
3r1j_B
3r2q_A
3r3r_A
3r3t_A
3r3y_A
3r3z_A
3r44_A
3r4c_A
3r4z_B
3r5c_A
3r5c_B
3r5y_D
3r6f_A
3r6o_A
3r6q_H
3r6s_B
3r6w_A
3r79_B
3r8x_A
3r98_A
3r9c_A
3r9g_A
3r9i_D
3r9j_A
3r9r_A
3rc7_A
3rcm_A

3rcy_F
3rcy_F
3rdk_A
3rdq_A
3re8_A
3red_H
3rez_C
3rfe_A
3rfu_D
3rfx_B
3rg0_A
3rg2_C
3rgb_B
3rgg_A
3rgu_C
3rgw_L
3rgz_A
3rh7_A
3rh7_C
3rha_A
3rhd_C
3rhh_D
3rht_A
3rht_C
3ric_A
3rii_A
3rir_A
3rk1_B
3rkr_A
3rku_D
3rlb_B
3rms_C
3rnm_C
3ro6_C
3ro6_C
3ro8_C
3ro8_F
3ro9_B
3roe_D
3rop_B
3rp8_A
3rpd_A
3rpe_A
3rq1_B
3rq5_A
3rqd_A
3rqi_A
3rqq_A
3rqw_A
3rqw_H
3rr5_A
3rra_B
3rrm_A
3rrv_B

3rrw_A
3rs8_A
3rsb_B
3rsc_B
3rsn_A
3rss_A
3rt0_A
3rt1_C
3rtk_A
3rto_D
3rtx_A
3rtx_B
3rv0_A
3rv0_B
3rwb_D
3rxa_A
3rxl_A
3rxm_A
3rxz_C
3ry2_B
3ryo_I
3ryp_A
3ryz_A
3s1e_A
3s1j_A
3s1v_A
3s21_A
3s2r_B
3s2y_A
3s2z_A
3s3g_A
3s55_C
3s5f_B
3s6b_A
3s6h_A
3s6v_A
3s70_A
3s7o_A
3s7q_A
3s7z_B
3s86_B
3s8a_A
3s9n_A
3sai_B
3san_B
3sao_B
3sba_B
3sbx_F
3sc6_D
3scq_A
3scv_A
3scz_A
3sd9_A
3sdj_D

3sdj_E
3ser_A
3sew_A
3sfv_A
3sfx_B
3sfz_A
3sgi_A
3sh5_A
3shq_A
3shr_A
3shs_A
3shs_A
3sik_A
3sip_F
3six_A
3siy_B
3sja_I
3sjn_A
3sjn_B
3skd_A
3sl9_B
3smp_B
3smv_A
3so1_H
3som_L
3son_A
3soy_A
3sp4_B
3sqg_D
3sra_B
3ss5_C
3ss7_X
3st1_A
3stp_A
3sub_A
3sw1_B
3swf_B
3sws_C
3sxm_A
3sxn_A
3sxn_D
3sxp_D
3sy8_B
3sy8_B
3sz3_A
3sz4_A
3t0u_B
3t1r_C
3t33_A
3t35_D
3t37_A
3t3o_A
3t3w_E
3t3w_F

3t3z_D
3t44_A
3t4k_A
3t4r_A
3t54_A
3t58_B
3t5p_L
3t66_A
3t6f_A
3t6q_A
3t6s_B
3t8i_A
3t8i_C
3t8q_B
3t9k_A
3t9q_B
3tat_C
3taw_A
3taz_A
3tb6_A
3tb8_A
3tbd_A
3tbm_B
3tbo_A
3tc1_B
3tc3_A
3tc3_B
3tcy_A
3td3_F
3td7_A
3td9_A
3tdp_A
3tdt_A
3te9_A
3teb_B
3ten_B
3tep_A
3tfa_B
3tfd_A
3tfg_A
3tfj_A
3tfq_D
3tia_B
3tia_D
3tik_C
3tj7_C
3tj9_A
3tji_D
3tk7_A
3tlj_A
3tmg_A
3tnj_A
3tnp_F
3tnz_A

3tou_A
3tox_G
3tox_I
3toz_G
3tp4_B
3tp9_A
3tpv_B
3tpw_A
3tq5_A
3tq8_A
3tqh_A
3tqu_A
3tri_A
3tsa_B
3tsn_C
3tts_B
3tts_D
3tts_E
3ttv_D
3tu6_A
3tui_C
3tur_A
3tuv_A
3tva_A
3tvi_E
3tvk_A
3tw4_A
3twa_A
3twb_C
3ty5_B
3ty7_B
3tz6_A
3tzf_A
3tzo_B
3u02_A
3u0d_A
3u0o_B
3u1b_A
3u2t_A
3u2z_D
3u3d_A
3u4a_B
3u4c_A
3u4j_A
3u4m_A
3u50_C
3u56_A
3u88_B
3u88_C
3u8v_A
3u95_B
3u9s_L
3ua3_A
3uan_A

3ubl_A
3ubm_B
3ubo_A
3uc1_A
3uce_C
3ucz_P
3ue6_F
3ue9_C
3ued_C
3uee_A
3ueh_A
3uei_B
3uf6_B
3ugt_C
3ugx_B
3uh1_A
3uh4_A
3uhs_A
3ujh_B
3ujp_B
3ujz_A
3ulq_A
3um9_A
3umv_A
3unr_A
3uns_A
3unv_A
3unw_A
3uoi_S
3up1_A
3upl_A
3uq6_A
3urm_A
3urn_A
3urp_A
3usk_C
3usx_B
3usz_A
3ut0_A
3utf_A
3uue_A
3uum_A
3uve_D
3uw1_A
3uwk_A
3uww_A
3ux3_B
3uxi_A
3uxj_A
3uxy_C
3uy9_D
3uy9_L
3uyk_A
3uzx_A

3v00_C
3v0c_A
3v0l_A
3v0p_A
3v17_B
3v17_D
3v1c_A
3v1h_A
3v34_B
3v3h_B
3v3n_D
3v65_C
3v65_C
3v6i_B
3v6o_B
3v8d_A
3v9l_A
3v9l_B
3v9o_A
3va8_A
3vay_B
3vb0_D
3vc2_G
3vdn_B
3ved_B
3ven_A
3vgk_B
3vgk_D
3vh7_E
3vh7_E
3vij_A
3vkc_A
3vkc_B
3vkd_A
3vll_A
3vlu_A
3vm5_A
3vmq_A
3vn4_A
3vni_C
3vnq_A
3vnz_A
3vof_A
3vol_A
3vov_B
3vp5_A
3vpb_F
3vpc_B
3vq2_C
3vqj_A
3vqr_A
3vr1_D
3vrg_A
3vse_A

3vse_B
3vsh_D
3vsq_A
3vtf_A
3vtk_A
3vto_Q
3vur_A
3vv1_A
3vvf_B
3vvk_F
3vw9_A
3vwd_A
3vx3_A
3vyg_L
3vyl_G
3vym_A
3vyr_B
3vyw_D
3vzw_X
3w00_B
3w0f_A
3w0l_B
3w0o_A
3w15_A
3w1b_A
3w1o_A
3w1q_A
3w1q_B
3w1u_A
3w1x_A
3w20_A
3w2l_B
3w2w_B
3w2z_A
3w5j_A
3w5m_A
3w5n_A
3w5u_E
3w68_B
3w6s_A
3w6s_A
3w6z_A
3w79_B
3w82_A
3w87_A
3w8f_A
3w8w_A
3w9f_A
3w9k_A
3wad_A
3wag_B
3wak_A
3waq_A
3war_A

3wbb_C
3wcp_A
3wcp_D
3wcu_D
3wcu_E
3wd6_A
3wdu_A
3wdv_A
3wec_A
3wew_A
3wf3_A
3wfw_A
3wgk_B
3wgu_B
3wgv_E
3wh7_A
3whc_A
3whd_C
3wip_B
3wip_F
3wky_A
3wl3_C
3wnw_F
3wok_B
3wqa_B
3wqg_B
3wqm_A
3wqz_A
3wr2_C
3wr4_A
3wr5_A
3wrb_B
3wre_A
3wro_A
3wry_A
3wry_C
3wsf_E
3wsi_A
3wsi_B
3wst_J
3wsw_C
3wv6_A
3wv9_C
3wve_B
3wvn_A
3ww1_A
3ww2_A
3ww3_B
3wwh_A
3wwj_C
3wwm_A
3wwo_B
3wwq_L
3wwt_B

3wxb_A
3wxx_G
3wy0_B
3wy1_A
3wy2_B
3wyc_B
3wzn_A
3x0n_A
3x0y_D
3x0y_F
3x15_D
3x15_J
3x21_H
3x27_D
3x2f_A
3x2x_B
3x43_A
3x43_B
3zbn_A
3zbq_A
3zby_B
3zc0_B
3zc0_C
3zc0_H
3zc0_K
3zc1_C
3zc7_A
3zcn_A
3zcx_A
3ze3_D
3zec_A
3zet_B
3zfj_A
3zfk_A
3zg1_B
3zg3_A
3zgl_A
3zgz_D
3zgz_D
3zi8_A
3zi8_A
3zid_A
3zj0_A
3zja_A
3zjh_B
3zjl_A
3zkb_H
3zl8_A
3zlm_A
3zm8_A
3zme_B
3zmk_B
3zmr_A
3zmu_A

3znv_A
3zoe_B
3zof_B
3zog_A
3zow_A
3zow_F
3zow_G
3zox_B
3zp4_B
3zp7_B
3zq5_A
3zqs_B
3zqx_A
3zr0_A
3zr4_B
3zrp_A
3zrr_B
3zs1_D
3zs7_A
3zsw_B
3zth_A
3zu1_B
3zu2_A
3zuk_A
3zv5_A
3zvi_B
3zvl_A
3zvl_A
3zwa_A
3zwa_B
3zwm_C
3zwm_D
3zwo_B
3zx4_B
3zxo_B
3zxu_A
3zxu_D
3zyf_B
3zyo_A
3zyy_Y
3zzn_D
4a0g_A
4a0z_A
4a1o_A
4a1o_B
4a2a_B
4a2b_A
4a3q_B
4a42_A
4a45_A
4a46_C
4a4a_A
4a4e_A
4a4f_A

4a4k_A
4a4k_G
4a61_A
4a62_B
4a69_B
4a69_C
4a6j_F
4a6o_A
4a6s_D
4a6t_B
4a8h_B
4a99_A
4ab7_H
4aba_A
4abo_D
4aci_B
4adc_A
4adj_C
4aea_A
4aeo_A
4af1_A
4afx_A
4afy_B
4ag3_A
4ag5_C
4agl_A
4aia_E
4aip_A
4aiw_A
4ajx_L
4ak7_B
4alb_A
4all_B
4am7_B
4am8_B
4amv_A
4ang_B
4ao5_E
4ao5_F
4ap5_B
4apm_A
4aq0_A
4aq6_D
4aqn_B
4aqq_A
4arc_A
4are_A
4arq_B
4ary_B
4arz_B
4as1_B
4as2_C
4as7_A
4ask_A

4aty_A
4au7_B
4aub_B
4av0_A
4avi_A
4avu_B
4awd_A
4axd_A
4axl_A
4axn_B
4axn_B
4axo_B
4axv_A
4ayb_C
4ayb_C
4ayg_A
4ayl_A
4azu_D
4b02_C
4b0n_A
4b17_A
4b1l_A
4b1u_M
4b1u_M
4b3j_A
4b3s_B
4b3s_E
4b43_A
4b44_A
4b46_A
4b4o_A
4b5q_B
4b5w_B
4b60_B
4b6z_B
4b6z_B
4b8n_D
4b96_A
4b98_C
4b98_D
4ba0_A
4ba9_C
4baa_C
4bb2_B
4bb5_D
4bb9_A
4bbo_B
4bbr_M
4bc2_A
4bca_A
4bcx_A
4ber_A
4bf4_I
4bf4_K

4bf5_A
4bfa_A
4bfi_A
4bhq_A
4bif_A
4bif_B
4bif_B
4bin_A
4bio_A
4biu_F
4biv_B
4biw_A
4biy_C
4biz_C
4bj8_O
4bja_A
4bju_A
4bjz_A
4bkm_C
4bkt_I
4bl6_C
4blb_A
4blv_A
4bmb_A
4bmp_B
4bmq_A
4bmv_C
4bn8_A
4bo0_B
4bol_A
4bos_B
4bpm_A
4bpz_B
4bq0_B
4bql_A
4bqn_A
4bqo_A
4br5_A
4br6_A
4br7_A
4bre_A
4brh_A
4brs_A
4bs1_A
4bt6_A
4btj_B
4buv_A
4bv1_D
4bvb_A
4bwr_A
4bww_C
4bxc_B
4by6_A
4bz8_B

4bz9_A
4bzi_E
4bzi_G
4c0c_A
4c0j_A
4c0n_A
4c1l_B
4c1n_I
4c1n_K
4c1r_A
4c1r_B
4c1y_A
4c20_B
4c2a_A
4c2l_A
4c2m_B
4c2v_B
4c2w_A
4c2w_B
4c3b_D
4c3b_F
4c3b_H
4c3b_M
4c3e_O
4c3i_L
4c3x_D
4c3x_H
4c3y_F
4c5k_A
4c5k_D
4c5l_B
4c79_A
4c7a_A
4c8i_B
4cae_A
4caj_D
4cat_B
4cc1_B
4cc7_K
4cc9_B
4ccv_A
4ccz_A
4cd6_A
4cdp_A
4ce4_5
4cff_C
4cfz_A
4cgk_B
4cht_A
4ci7_A
4cin_A
4cj8_H
4cjx_A
4ck7_B

4ckc_D
4clk_A
4clo_D
4cm1_A
4cmi_A
4cmi_B
4cmn_A
4coq_B
4cov_A
4cox_D
4coy_A
4cpa_B
4cpa_I
4cpo_A
4cq5_D
4cqb_A
4cr8_D
4crn_P
4crv_B
4cs7_E
4cs8_B
4csm_B
4ct4_D
4cta_A
4cth_A
4cu9_B
4cub_A
4cub_B
4cug_A
4cv1_B
4cvh_A
4cvo_A
4cvq_A
4cvu_A
4cw0_A
4cxs_A
4cy8_A
4cyb_J
4cyk_A
4cym_A
4cys_B
4czm_A
4czy_B
4d05_A
4d0l_D
4d0z_E
4d1y_B
4d25_A
4d2i_A
4d3d_A
4d3l_B
4d3u_A
4d49_E
4d4g_A

4d4o_A
4d65_B
4d6u_G
4d6x_B
4d79_C
4d7e_A
4d7u_A
4d82_A
4d92_D
4d9q_B
4db3_A
4dcm_A
4ddr_A
4deu_B
4df0_B
4df1_A
4df3_B
4dfd_A
4dg8_A
4dik_A
4dit_A
4djd_B
4dkd_A
4dky_B
4dmg_A
4dn1_A
4dn2_A
4dn5_A
4dnj_A
4dnm_A
4dnn_B
4dnr_A
4dnz_C
4doz_A
4dpg_J
4dpl_A
4dq6_B
4dqk_A
4dr4_O
4dr6_H
4dr7_J
4dr8_A
4dt9_B
4dty_A
4dun_A
4dur_A
4duy_F
4duz_J
4dv0_C
4dv1_I
4dv2_F
4dve_B
4dwd_B
4dwj_B

4dwo_A
4dxw_B
4dy7_F
4dz6_D
4dzi_A
4dzr_A
4e0c_A
4e0f_A
4e0i_A
4e0u_B
4e13_A
4e1b_A
4e2g_C
4e2q_C
4e3w_B
4e45_D
4e45_J
4e4s_F
4e51_A
4e55_B
4e5m_B
4e5p_A
4e5v_A
4e5y_A
4e6k_G
4e7e_A
4e7e_D
4e7u_D
4e7v_B
4e7w_A
4e80_C
4e84_A
4e8c_A
4ean_B
4eay_C
4eb6_D
4ebu_A
4ec0_B
4ec5_B
4ece_E
4edy_B
4ee3_C
4eei_B
4een_A
4ef8_B
4efz_A
4eg1_B
4eg9_A
4eg9_A
4egp_A
4egs_A
4ehc_A
4ehu_B
4ei5_A

4ei9_A
4eia_A
4eis_B
4eiw_E
4ej0_F
4ej6_A
4ejx_D
4ek1_A
4elb_B
4elb_B
4elb_H
4elj_A
4elr_A
4emj_A
4emy_A
4enl_A
4enp_B
4eo1_A
4eo3_A
4eo7_A
4eoi_C
4ep4_B
4eq5_A
4eq9_A
4eqx_B
4erm_E
4erp_D
4erp_E
4esn_A
4eso_A
4eu5_A
4euk_A
4euo_A
4ev0_D
4ev2_A
4evi_A
4evw_B
4ew7_A
4ewg_A
4ewl_A
4ex6_A
4exa_B
4exa_F
4exl_C
4exo_A
4exx_B
4ey0_D
4eyk_A
4f07_C
4f07_G
4f07_J
4f0p_B
4f0q_A
4f1c_B

4f1m_A
4f1z_A
4f2l_A
4f2n_L
4f30_A
4f53_B
4f5w_A
4f5x_F
4f5x_L
4f5y_A
4f5y_B
4f66_B
4f7w_A
4f86_A
4f86_C
4f86_D
4f8b_C
4f8e_A
4f8e_B
4f8p_B
4f96_A
4fa1_E
4fai_A
4fai_B
4fak_A
4fba_A
4fbb_B
4fbk_A
4fd2_D
4fdg_A
4fdi_A
4fdi_B
4fe7_A
4ffd_A
4ffd_A
4ffk_A
4fg9_B
4fha_A
4fhc_A
4fhg_A
4fht_B
4fi3_C
4fid_A
4fig_A
4fir_A
4fir_D
4fix_B
4fk1_B
4fl2_A
4flm_B
4fm4_K
4fma_B
4fmo_A
4fms_A

4fmw_A
4fn4_B
4fnd_A
4fo9_A
4fp9_C
4fpp_B
4fqg_B
4fr2_A
4ft4_B
4ft8_B
4fu0_A
4fva_C
4fva_C
4fyp_A
4fzb_C
4fzb_E
4fzm_A
4g01_A
4g0y_A
4g10_A
4g19_A
4g1e_B
4g2s_B
4g2t_A
4g33_A
4g3m_A
4g41_B
4g4y_F
4g5h_A
4g5r_D
4g5s_A
4g67_A
4g6u_A
4g6z_A
4g75_A
4g8a_D
4g8j_B
4g97_A
4g9g_A
4g9k_A
4g9n_B
4ga6_A
4gbc_B
4gbz_A
4gc9_A
4gcy_A
4gcz_B
4gd3_A
4gd3_B
4gd9_B
4gdi_A
4gdp_B
4gdz_A
4ge1_A

4gej_I
4gel_B
4gem_B
4gey_A
4gf0_A
4gf5_G
4gf5_M
4gf5_P
4gfj_A
4gg2_B
4ggj_A
4ggk_A
4ggz_D
4gh1_A
4ghg_A
4ghl_A
4ghm_A
4gi2_B
4gi5_B
4gi7_C
4gib_A
4gil_B
4gir_B
4gis_A
4gis_B
4gjz_A
4gk9_A
4gkb_C
4gko_L
4gl7_A
4gll_A
4gme_C
4gmg_A
4gn0_C
4gna_B
4gnc_A
4gox_A
4gpc_A
4gpu_A
4gqk_A
4gqy_A
4gr1_A
4grf_A
4grh_A
4gsf_B
4gsl_A
4gua_B
4guh_B
4gun_C
4gun_J
4gun_P
4gux_B
4gvo_A
4gvx_B

4gw9_A
4gw9_C
4gw9_D
4gwb_A
4gwm_A
4gxw_A
4gy5_C
4gza_H
4gzt_B
4gzt_C
4h03_A
4h0f_A
4h0f_B
4h0j_A
4h0j_D
4h0l_G
4h0m_C
4h18_D
4h19_P
4h1v_A
4h23_A
4h2d_A
4h2l_A
4h2u_D
4h2w_C
4h3s_A
4h3z_B
4h4e_B
4h5f_A
4h6d_B
4h6q_A
4h7l_B
4h83_F
4h8n_A
4h97_B
4h9k_A
4ha3_A
4ha9_A
4hac_A
4hai_A
4han_A
4haw_C
4hb9_A
4hbg_A
4hcf_B
4hd4_B
4hey_A
4hg0_A
4hg5_A
4hg6_A
4hgn_B
4hgn_C
4hgq_D
4hgx_B

4hh1_A
4hh4_C
4hi0_E
4hi1_A
4hia_A
4hj6_B
4hji_A
4hjw_C
4hk5_A
4hki_A
4hlm_B
4hlu_B
4hlu_C
4hnb_A
4hnh_A
4hnn_F
4hnz_E
4hnz_L
4hog_A
4hot_A
4how_A
4hpb_A
4hpg_A
4hql_A
4hqm_B
4hqo_A
4hr3_A
4hrq_B
4hrt_F
4hs4_H
4hs9_A
4hss_A
4hsw_B
4hte_A
4htf_A
4huz_A
4hv4_A
4hvj_B
4hwv_A
4hx2_B
4hx3_I
4hx3_I
4hx5_B
4hy4_B
4hyj_A
4hz2_B
4i02_C
4i14_A
4i25_D
4i2r_A
4i3g_B
4i3m_A
4i3x_A
4i42_F

4i45_A
4i4i_A
4i58_D
4i5e_A
4i5q_A
4i6v_A
4i7f_A
4i8v_B
4i94_A
4i9r_A
4i9x_B
4iao_B
4iau_A
4ib2_A
4ibo_D
4id4_A
4idc_A
4idg_B
4ie6_A
4iee_A
4ieg_C
4iei_A
4iej_A
4iej_A
4ifh_A
4ifo_B
4ifr_B
4ifu_A
4igm_E
4ih3_B
4ihb_E
4ihh_I
4ii1_B
4ii1_B
4iil_A
4ijr_A
4iju_B
4ijx_A
4il3_B
4ild_B
4ild_B
4ilo_A
4ilv_B
4im7_A
4imp_A
4imr_A
4ine_A
4inf_B
4inf_C
4inj_A
4io7_B
4iol_A
4ip2_C
4ip2_C

4ipj_A
4ips_A
4iqg_D
4iqy_A
4ir7_A
4ir7_A
4is3_C
4isk_B
4isk_B
4itb_A
4itk_A
4itm_A
4itx_B
4itx_B
4iu3_A
4ium_A
4iuu_B
4iuw_A
4iv6_B
4iv9_B
4ivv_A
4iw3_A
4iw9_B
4ix0_A
4ix5_B
4ix6_B
4ixj_B
4ixo_B
4ixq_j
4ixr_E
4ixr_m
4iyb_A
4iz6_A
4iz9_A
4izk_B
4j0i_B
4j14_A
4j1q_A
4j1w_A
4j1x_C
4j20_A
4j2k_A
4j34_B
4j3f_D
4j48_C
4j5w_D
4j6g_C
4j6o_A
4j6r_G
4j6x_A
4j6x_D
4j8l_A
4j90_A
4j96_B

4jaf_B
4jag_A
4jap_C
4jb0_A
4jbb_A
4jbh_A
4jd1_B
4jdp_B
4je5_A
4je7_A
4jed_A
4jem_A
4jet_I
4jf7_B
4jfa_B
4jfn_A
4jgi_B
4jgo_B
4jgt_A
4jgu_A
4jgu_B
4jhd_E
4jhh_A
4ji0_E
4ji3_H
4ji4_C
4jiu_A
4jiw_I
4jiw_L
4jix_A
4jj2_C
4jkr_K
4jmg_A
4jna_A
4jnx_D
4jo5_A
4jou_A
4jpc_A
4jph_A
4jqi_L
4jqo_B
4jrf_A
4js2_A
4jsd_A
4jtq_A
4jvt_A
4jwf_B
4jwj_B
4jwt_A
4jx4_C
4jx9_A
4jy3_A
4jyb_A
4jyk_A

4jys_A
4jyu_H
4jyu_H
4jyv_H
4jyy_A
4jyz_A
4jzg_A
4jzt_A
4k03_B
4k08_A
4k0b_A
4k0g_A
4k0j_A
4k17_C
4k1i_B
4k1k_A
4k24_U
4k2h_D
4k2p_B
4k30_A
4k5r_A
4k5r_B
4k5u_B
4k6b_A
4k6b_B
4k6f_B
4k6f_D
4k6n_A
4k6u_B
4k7i_A
4ka7_A
4kbz_A
4kcf_A
4kdr_A
4kdx_B
4kdy_A
4kem_A
4ket_D
4kf1_B
4kfr_B
4kfv_A
4kg0_A
4kgi_C
4kgn_C
4kgq_C
4kh6_B
4kh7_B
4kh9_A
4kho_A
4ki0_A
4kjy_A
4kjz_C
4kkd_B
4kkj_A

4kkv_A
4kky_X
4kla_A
4km6_A
4kmg_A
4kmr_B
4kmv_B
4kna_A
4kng_B
4knh_B
4knl_B
4knl_C
4knw_B
4ko6_C
4koa_A
4kob_B
4kob_C
4koo_A
4kp1_A
4kp4_A
4kp8_D
4kpn_G
4kpp_A
4kpp_A
4kpu_B
4kq6_C
4kq6_I
4kqa_B
4kqq_A
4kr5_B
4krg_B
4kry_A
4ksa_A
4ktp_A
4ktr_A
4ktz_B
4ku0_D
4ku2_B
4kub_A
4kv0_A
4kvb_M
4kvo_G
4kvp_B
4kwa_A
4kwc_A
4kwe_C
4kwh_B
4kwl_A
4kws_F
4kx4_A
4kx6_D
4kxb_A
4kxf_K
4kxy_B

4ky8_E
4l0e_A
4l0p_B
4l0p_B
4l1k_A
4l2a_A
4l39_A
4l3t_A
4l44_A
4l54_A
4l6a_A
4l6d_B
4l6d_C
4l6t_A
4l79_A
4l80_B
4l82_B
4l8h_B
4l8n_A
4l8v_A
4l97_A
4l9p_B
4laf_A
4lag_X
4lc5_A
4lc8_A
4lc8_B
4lec_A
4leh_A
4lej_A
4lem_F
4lep_A
4lev_A
4lf2_A
4lf2_B
4lf5_N
4lf8_G
4lf9_E
4lfb_L
4lg1_A
4lgd_B
4lh8_A
4lh8_A
4lhy_A
4li4_A
4lim_A
4lin_G
4lit_A
4lji_A
4lk2_A
4lkt_D
4lmx_C
4lna_A
4lo1_A

4lo9_B
4los_A
4lp7_D
4lpa_A
4lpc_B
4lqf_A
4lqx_B
4lrh_D
4lrl_C
4lsb_B
4lsm_A
4lta_A
4ltp_B
4lv5_A
4lv5_B
4lwp_B
4lxc_D
4lxo_B
4ly4_D
4lyk_D
4lzb_F
4lzd_A
4lzz_I
4lzz_U
4m01_B
4m0j_B
4m0j_B
4m0n_A
4m1e_F
4m1p_A
4m1t_C
4m20_A
4m2g_B
4m2j_A
4m4j_D
4m51_A
4m52_C
4m56_B
4m58_A
4m5p_A
4m6q_A
4m6r_C
4m6t_A
4m7e_A
4m7u_A
4m7y_B
4m83_A
4m8l_D
4ma3_A
4mae_A
4mai_A
4mar_A
4mb2_B
4mb7_A

4mbx_A
4mby_I
4mc5_C
4mc7_B
4md9_I
4mdp_A
4mdt_B
4mdz_B
4me3_A
4mf9_A
4mgk_E
4mgq_A
4mhq_A
4mhu_A
4mhv_A
4mih_D
4mih_E
4miv_A
4mj7_A
4mjl_A
4mk3_A
4ml9_A
4mlc_A
4mle_A
4mlr_F
4mmo_A
4mn5_A
4mn6_B
4mnd_A
4mnr_A
4mo1_A
4mo5_D
4mo7_A
4mok_C
4moq_B
4mor_A
4mp8_A
4mpg_B
4mpy_H
4mpz_A
4mqk_G
4mqy_A
4mra_A
4mrb_B
4msu_A
4msx_A
4msx_A
4mtl_B
4mtu_A
4muq_A
4mv2_B
4mv4_A
4mxe_A
4my0_F

4myh_B
4myo_A
4mys_B
4myx_C
4myx_E
4mz0_A
4mza_B
4mzf_B
4mzg_B
4n09_C
4n0b_A
4n0b_B
4n0g_C
4n0i_A
4n0l_A
4n1i_A
4n27_B
4n35_A
4n3v_B
4n48_B
4n4b_A
4n4g_A
4n4r_C
4n4t_A
4n54_B
4n54_D
4n58_B
4n5h_X
4n7b_A
4n7n_E
4n7p_F
4n7s_B
4n82_B
4n8f_A
4n8j_D
4n90_A
4n9f_S
4n9i_B
4nbb_D
4nbb_E
4nbd_E
4nbg_E
4nc7_B
4nc8_B
4ndz_F
4nea_A
4nec_B
4nef_A
4nei_A
4neu_A
4nf1_G
4nfu_A
4ng3_C
4nga_H

4nge_A
4nh4_A
4nhb_A
4nhb_B
4nhe_C
4nhf_F
4nhw_D
4nhy_A
4nhy_B
4nhz_H
4nhz_P
4ni8_F
4nia_F
4nj4_A
4nje_A
4njm_A
4nk2_A
4nkb_B
4nkt_A
4nkv_C
4nl5_A
4nmd_B
4nmk_E
4nmy_A
4nn3_A
4nna_A
4nnw_G
4nnw_K
4no4_F
4no6_H
4no8_K
4nob_A
4np7_A
4np9_A
4npp_A
4nsq_B
4nsq_C
4nt5_A
4ntc_A
4ntk_A
4ntn_A
4ntn_B
4nu0_B
4nu6_B
4nu7_C
4nun_A
4nun_A
4nv0_B
4nv2_A
4nvr_A
4nvr_B
4nwo_A
4nxb_A
4nxv_A

4nxv_C
4nyq_A
4nz2_B
4nz6_B
4nzd_C
4nzf_D
4nzg_B
4o01_D
4o0l_B
4o1e_A
4o1m_A
4o1m_E
4o2i_A
4o2w_B
4o30_A
4o33_A
4o4o_A
4o4p_A
4o4s_A
4o5m_A
4o5q_A
4o65_A
4o6o_D
4o6p_B
4o6z_A
4o7i_A
4o8c_B
4oa5_E
4obb_A
4obi_A
4obu_F
4obw_C
4oci_A
4odo_C
4oec_A
4oev_A
4ofc_C
4ofg_A
4ofo_D
4ofq_A
4ofz_A
4ogk_E
4ogl_C
4ogl_E
4ohf_A
4ohf_B
4ohn_A
4oht_A
4ohy_A
4oic_A
4oiw_B
4oiw_E
4ojj_C
4ojm_X

4ojo_A
4oju_C
4ojy_A
4ojy_A
4ojy_B
4ojz_A
4okd_A
4oko_A
4oku_B
4ol9_A
4olk_B
4om8_B
4on1_A
4onr_A
4onv_B
4onx_A
4oo7_B
4ooj_C
4oow_A
4ooz_A
4op0_A
4oq9_E
4oq9_K
4oro_A
4oro_B
4osd_G
4oul_E
4ov1_A
4ov9_A
4ovd_A
4ove_A
4ovr_B
4ovt_B
4ovy_A
4owd_A
4owk_E
4owz_B
4ox3_A
4oxx_A
4oy2_A
4oy7_A
4oy7_A
4oyz_A
4oz1_A
4oz6_A
4ozq_B
4ozv_A
4p0d_A
4p10_A
4p1g_A
4p36_A
4p3l_A
4p42_A
4p4s_B

4p4t_A
4p57_B
4p5b_B
4p5e_B
4p5f_A
4p5w_A
4p5w_A
4p6v_B
4p6v_D
4p6v_E
4p77_A
4p7x_A
4p86_A
4p8q_A
4p8y_B
4p9e_A
4pa5_A
4pac_A
4paf_A
4pag_A
4pao_A
4paw_A
4pbg_A
4pbu_j
4pbu_O
4pc0_B
4pc2_B
4pc6_B
4pdd_A
4pde_A
4pdn_A
4pdy_A
4pee_C
4pem_A
4pew_B
4pfu_B
4pfx_A
4pg3_C
4pg7_A
4pgl_C
4pgp_D
4phc_A
4phg_A
4phh_C
4pi0_E
4pie_A
4pii_A
4pj2_B
4pk5_A
4pks_A
4pkv_A
4pl0_B
4pl9_A
4plv_D

4ply_C
4pmw_A
4pmx_A
4pne_B
4pnl_D
4pof_F
4poq_H
4por_A
4pot_F
4pp6_A
4pq6_A
4pqg_A
4pqh_A
4pqw_A
4pr3_A
4pr3_B
4ps2_A
4psw_A
4psw_B
4ptn_A
4ptz_D
4pv6_F
4pv6_G
4pv6_I
4pw3_C
4pw9_B
4pwa_D
4pwb_A
4pwg_A
4pwt_C
4pwy_A
4px3_B
4px5_B
4pxa_A
4pxg_A
4pxo_A
4pxv_D
4pxz_A
4pyg_A
4pyh_A
4pyn_A
4pyr_A
4pys_A
4pys_B
4pz1_A
4pz6_B
4pzd_F
4pze_H
4pzh_A
4pzl_A
4pzs_A
4q0k_A
4q0q_A
4q19_B

4q1v_A
4q2b_C
4q2g_B
4q2h_A
4q2t_A
4q33_F
4q4a_A
4q4k_A
4q4k_B
4q51_B
4q52_A
4q57_B
4q5h_A
4q93_A
4q9n_E
4qai_A
4qan_B
4qat_B
4qbl_E
4qbo_A
4qdk_A
4qdp_A
4qe6_A
4qed_B
4qei_A
4qek_A
4qfh_B
4qfs_C
4qfu_C
4qfu_G
4qgp_A
4qgp_A
4qgr_B
4qhi_B
4qht_B
4qhy_A
4qhy_A
4qhz_B
4qid_A
4qim_A
4qiq_A
4qiz_B
4qj0_B
4qjb_A
4qjb_B
4qkf_B
4qkf_B
4qkr_A
4qlq_G
4qlq_K
4qlx_A
4qly_A
4qn1_A
4qn2_E

4qn3_A
4qn6_A
4qna_A
4qnl_A
4qod_B
4qoz_C
4qp2_A
4qpd_A
4qpj_B
4qpj_D
4qqf_A
4qra_A
4qrd_A
4qrh_B
4qrk_A
4qrn_A
4qro_E
4qry_A
4qsv_A
4qtq_A
4qts_C
4qtu_B
4qu3_A
4qu6_A
4quv_A
4qux_K
4quy_G
4qv5_G
4qv5_H
4qv8_I
4qv8_Y
4qve_B
4qvl_I
4qvl_J
4qvn_J
4qvt_D
4qvu_A
4qvv_G
4qw0_J
4qw6_N
4qw7_K
4qw7_Z
4qwu_G
4qz0_G
4qzw_G
4qzz_G
4r0a_A
4r0o_B
4r0o_D
4r0t_B
4r11_F
4r12_A
4r12_A
4r18_Z

4r1d_A
4r1m_C
4r1p_F
4r20_A
4r29_B
4r2b_B
4r2m_A
4r38_B
4r39_A
4r4u_A
4r51_A
4r51_B
4r57_A
4r57_F
4r6g_A
4r70_A
4r78_A
4r7o_C
4r7t_A
4r7w_E
4r81_B
4r83_D
4r84_A
4r88_D
4r8d_A
4r94_A
4rad_H
4rb4_E
4rck_A
4rda_A
4rdh_A
4rdi_B
4rdr_A
4rdw_A
4rdz_B
4rek_A
4rep_A
4ret_G
4rfq_A
4rfu_B
4rfu_B
4rg1_A
4rg8_A
4rgb_A
4rgw_A
4rhh_A
4rhm_A
4rhz_A
4rhz_B
4ri1_B
4ri2_B
4ri6_A
4ri7_B
4rih_B

4rii_B
4riy_A
4rjy_D
4rk0_D
4rk4_A
4rk6_A
4rkc_B
4rkk_A
4rkq_A
4rks_A
4rks_B
4rl3_A
4rl6_A
4rla_B
4rld_C
4rld_C
4rle_A
4rlu_A
4rmo_A
4rn3_A
4rn7_A
4rnu_C
4rnx_B
4rny_A
4rot_A
4rqf_B
4rqi_D
4rqn_A
4rqo_A
4rr2_C
4rrf_C
4rrs_A
4rs2_B
4rsl_A
4rso_A
4rt5_A
4rtz_A
4ru1_A
4ru1_B
4ru4_F
4rud_B
4ruq_B
4ruw_A
4ruz_A
4rvu_A
4rvy_O
4rwg_B
4rwk_A
4rxt_A
4rxu_A
4rxz_B
4ry9_B
4rya_A
4ryt_A

4rz3_B
4rzx_A
4s17_B
4s1m_A
4s1m_A
4s23_A
4s29_A
4s2a_A
4s2u_A
4s37_H
4sdh_A
4sgb_E
4tk5_B
4tkf_D
4tko_B
4tkq_A
4tkr_B
4tlb_E
4tlc_E
4tlf_D
4tma_B
4tmv_B
4tn1_A
4tnd_A
4tnh_x
4tnj_k
4tnj_V
4tnk_E
4tnk_L
4tnu_A
4to9_J
4to9_K
4tod_H
4tof_A
4toi_A
4tor_A
4tpg_A
4tqg_A
4tr1_A
4tri_A
4ts2_X
4ts5_B
4tsd_B
4tss_A
4tv6_A
4twb_E
4twb_F
4twk_A
4twk_A
4tx3_A
4tx9_A
4txd_A
4tyd_K
4tyv_B

4u01_E
4u0w_A
4u1q_D
4u3e_A
4u3e_A
4u49_B
4u49_B
4u4t_A
4u4t_A
4u4x_A
4u5p_A
4u63_A
4u6i_C
4u6x_B
4u75_A
4u7k_D
4u7w_B
4u82_A
4u8h_A
4u8h_D
4u8u_I
4u8u_M
4u8u_o
4u8u_Z
4u9w_B
4ua3_A
4uah_C
4uat_A
4uau_A
4uav_A
4ub8_V
4ub9_B
4ub9_C
4ub9_G
4ubt_A
4uc1_A
4uc8_B
4ucy_A
4udj_B
4udj_C
4udk_E
4udr_A
4udr_B
4ug4_F
4uhd_A
4uhg_A
4uhq_B
4uii_A
4uir_A
4umb_B
4umv_A
4un0_C
4uni_A
4unm_B

4unm_B
4uob_A
4uof_C
4uoj_B
4uop_B
4uoq_A
4uov_C
4uox_C
4up9_A
4upl_A
4uq6_B
4uq8_F
4uqi_A
4uqq_A
4uqs_A
4uqv_G
4uqv_J
4us5_C
4usr_A
4usu_A
4ut4_A
4utg_A
4uu2_A
4uui_A
4uun_B
4uux_A
4uvq_A
4uw0_A
4uw9_A
4uwm_B
4ux3_A
4uxa_R
4uxh_A
4uxh_B
4uxp_C
4uxz_D
4uy7_A
4uyq_A
4uyr_A
4uzv_A
4v07_A
4v08_A
4v0k_B
4v0v_A
4v0v_C
4v19_5
4v19_9
4v19_Q
4v19_R
4v1a_x
4v1y_E
4v27_A
4v2i_A
4v32_B

4v35_A
4v3o_D
4w1v_A
4w4o_C
4w5t_A
4w66_A
4w6q_C
4w7j_C
4w7n_B
4w82_A
4w87_A
4w8c_B
4w8f_B
4w8j_A
4w8l_B
4w8x_A
4wa0_A
4wad_A
4wae_A
4wai_A
4wat_A
4wb2_C
4wbd_A
4wbt_A
4wc2_A
4wc6_A
4wce_J
4wce_O
4wcf_C
4wcj_A
4wdt_A
4weo_D
4wer_A
4wf6_A
4wfa_C
4wfa_N
4wfa_W
4wfb_E
4wfg_G
4wfh_G
4wg2_A
4wgi_A
4wgw_C
4whb_A
4whi_A
4whm_A
4whr_D
4whx_A
4wig_A
4wio_A
4wiw_C
4wjm_A
4wm9_A
4wn3_A

4wn3_A
4wn9_D
4wnb_A
4wnh_A
4wnj_B
4wnx_A
4wo1_A
4wod_A
4wp8_B
4wpc_B
4wpt_A
4wqj_A
4wr3_A
4wrn_B
4wso_A
4wu2_A
4wu3_D
4wuv_A
4wvb_A
4wvz_D
4ww3_A
4ww7_A
4wwd_A
4wwu_C
4wwu_H
4wwu_J
4wwx_B
4wwx_B
4wx0_A
4wxe_A
4wxj_B
4wxv_A
4wxw_A
4wy0_I
4wyd_B
4wyl_A
4wyp_B
4wza_D
4wzf_B
4wzq_A
4x0o_E
4x0u_D
4x1t_A
4x20_B
4x2q_C
4x2v_D
4x2z_A
4x3k_B
4x3l_B
4x3n_A
4x3s_B
4x49_A
4x4r_A
4x4v_A

4x5d_A
4x62_T
4x63_A
4x64_T
4x66_T
4x7g_A
4x7r_B
4x8y_A
4x9d_D
4xa7_A
4xau_C
4xb1_B
4xb6_D
4xb6_G
4xb6_H
4xbm_B
4xbo_A
4xbz_C
4xbz_E
4xc3_H
4xc5_C
4xc7_B
4xcq_A
4xcv_A
4xcx_A
4xea_A
4xed_A
4xer_A
4xfa_A
4xga_A
4xgl_A
4xgp_B
4xgq_E
4xgu_B
4xgv_B
4xh7_A
4xhe_B
4xhg_A
4xhn_A
4xiv_B
4xj2_A
4xje_A
4xlo_B
4xlo_C
4xm2_D
4xmh_A
4xnh_A
4xnh_C
4xo6_A
4xoq_A
4xpl_A
4xps_A
4xqm_A
4xre_A

4xrf_A
4xrn_C
4xrp_D
4xsh_B
4xt0_A
4xt4_A
4xt5_A
4xun_A
4xup_C
4xut_C
4xva_G
4xvx_B
4xvz_C
4xw2_A
4xwp_A
4xx6_B
4xx9_A
4xxf_A
4xxf_A
4xxi_A
4xxi_B
4xxl_A
4xyb_B
4xym_A
4xym_B
4xz6_A
4xz9_C
4xzj_A
4y0e_H
4y0h_D
4y1q_M
4y2h_B
4y2w_B
4y3o_B
4y4j_A
4y4n_F
4y4v_B
4y5t_A
4y5u_B
4y67_A
4y6g_B
4y6l_A
4y6s_A
4y6z_N
4y6z_Z
4y70_Z
4y7o_B
4y7o_C
4y7s_A
4y7s_B
4y7x_G
4y7x_N
4y7y_G
4y8a_B

4y8g_G
4y8l_G
4y8p_Z
4y8s_I
4y8u_K
4y9l_B
4ya0_G
4ya2_G
4ya3_G
4yac_A
4yai_B
4yan_A
4yb5_C
4ybg_A
4ybr_B
4ybz_A
4ybz_D
4yc4_A
4yca_A
4yca_B
4ycu_B
4yda_A
4ydo_A
4ydo_A
4ydu_C
4yef_G
4yeh_B
4yer_A
4ygf_A
4ygf_G
4ygs_A
4yh2_D
4yhh_E
4yia_B
4yic_B
4yjh_A
4ykk_B
4ylr_A
4ymg_B
4ymi_B
4ymp_A
4ymu_D
4ymz_A
4yn0_A
4ynt_A
4yo8_A
4yo9_A
4yok_A
4yol_A
4yor_A
4yot_A
4yp5_B
4yp9_B
4ypl_F

4ypq_A
4ypt_A
4yrb_D
4yrc_A
4yri_A
4yse_C
4ysh_A
4ysn_C
4ysx_G
4ysy_C
4ysy_E
4yt5_B
4ytn_G
4yu9_C
4yut_B
4yw7_P
4ywa_D
4ywm_B
4ywm_G
4ywo_A
4ywr_A
4yxb_A
4yxo_A
4yxp_B
4yy3_N
4yzo_A
4yzo_C
4z0h_O
4z1k_A
4z1o_B
4z24_A
4z25_F
4z26_B
4z33_A
4z3u_B
4z4i_A
4z7f_B
4z7j_A
4z7m_B
4z89_B
4z8i_A
4z8w_A
4z94_G
4z94_G
4z9d_A
4z9d_C
4z9i_B
4z9k_A
4z9n_B
4z9v_A
4za6_B
4za8_A
4zad_A
4zan_A

4zbg_A
4zbt_D
4zef_A
4zel_A
4zew_B
4zfk_B
4zg5_C
4zgd_E
4zgj_M
4zgl_I
4zgp_A
4zgs_D
4zht_A
4zi5_A
4zia_B
4zil_A
4zir_A
4zir_B
4ziy_A
4zj0_B
4zjy_A
4zki_B
4zlh_B
4zlu_B
4zm3_A
4zm4_F
4zm5_B
4zm6_A
4zm9_B
4zmc_E
4zme_A
4zmi_A
4zmr_A
4zn3_B
4znd_A
4znf_A
4zp0_A
4zpi_C
4zqb_B
4zqf_A
4zr8_A
4zrb_G
4zrq_A
4zsi_A
4ztx_A
4zu3_E
4zu7_B
4zu7_C
4zvb_A
4zxs_A
4zxs_C
4zxs_C
4zxs_D
4zya_A

4zya_B
4zz7_A
4zz7_C
4zzj_A
5a05_D
5a13_G
5a29_A
5a2w_A
5a30_A
5a3m_D
5a4d_D
5a4e_C
5a5e_A
5a66_B
5a6b_B
5a76_E
5a7y_B
5a8v_A
5a95_A
5a96_A
5a9k_A
5a9t_A
5a9y_A
5aaq_A
5aaz_A
5ab7_D
5ab7_E
5aba_B
5ac4_B
5ad1_A
5adz_A
5ae3_C
5aed_A
5af0_C
5afu_I
5aga_A
5aga_A
5agd_B
5ags_A
5ah1_A
5ah5_A
5ah5_C
5ahs_C
5ahw_C
5aik_A
5aix_A
5aj3_c
5aj3_p
5aj3_R
5akp_A
5amj_C
5amk_B
5amk_D
5an1_C

5anz_A
5ao1_C
5aoi_B
5apw_B
5aqk_B
5aqu_B
5aqu_B
5ar2_B
5aup_I
5aur_G
5ave_A
5ax1_A
5ax7_B
5ayr_A
5ayr_B
5ayy_F
5az0_A
5aza_A
5azh_A
5b0h_A
5b0h_B
5b0o_A
5b0o_B
5b0o_C
5b0q_B
5b1y_B
5b3i_A
5b47_A
5b51_A
5b53_A
5b5t_D
5b6a_A
5b6d_B
5b6i_A
5b7c_A
5b7g_A
5b7z_B
5b82_A
5b85_A
5bj3_B
5bke_C
5bn2_A
5bnc_A
5bnt_D
5bo3_A
5bo6_A
5bou_N
5bp2_A
5bp7_B
5bp9_A
5bpx_A
5bqf_A
5bqm_C
5bqq_B

5bqt_D
5bsf_B
5bsh_C
5bst_A
5bt9_C
5btx_A
5bu2_C
5bul_A
5buq_B
5buv_A
5bv2_P
5bva_A
5bw4_B
5bwe_A
5bwe_B
5bwj_A
5bwj_A
5bxi_D
5bxr_B
5bxt_B
5bxy_B
5by0_A
5by3_A
5byh_D
5byu_B
5c03_B
5c03_B
5c0u_A
5c14_A
5c1o_C
5c1t_A
5c2i_A
5c2y_A
5c38_A
5c3j_E
5c3m_B
5c3o_A
5c55_A
5c5u_B
5c6f_L
5c6t_A
5c7e_D
5c88_A
5c8b_B
5c8d_E
5c8l_A
5cae_A
5cbk_A
5cc6_A
5ccx_A
5ccz_A
5cd2_A
5cd4_I
5cd6_B

5cd6_C
5cez_D
5cfa_A
5cfq_B
5cge_E
5cgg_J
5cgi_Z
5cgz_B
5ci7_A
5cj3_B
5cjf_A
5ck4_B
5ck7_A
5cm6_A
5cn0_A
5cnt_G
5cot_A
5cov_A
5cpo_B
5cq1_B
5cqk_A
5csd_D
5csl_B
5css_A
5csx_A
5cts_A
5cu1_A
5cu1_A
5cuj_A
5cuy_D
5cvd_A
5cvu_D
5cx6_B
5cx7_E
5cx7_H
5cx7_K
5cxk_F
5cxm_D
5cxx_B
5cyp_A
5cyr_A
5cz2_E
5cz2_J
5cz2_K
5cz8_K
5cz8_K
5cz9_L
5czc_B
5czd_B
5czo_D
5czy_A
5d0y_A
5d1o_A
5d1o_B

5d27_A
5d2e_A
5d2n_C
5d3x_A
5d41_A
5d6d_B
5d6o_B
5d6t_A
5d79_B
5d7z_A
5d88_A
5d8h_C
5d8x_E
5d9g_A
5d9p_B
5d9u_B
5da5_J
5da8_I
5da9_A
5day_B
5dbj_E
5dbx_A
5dcx_A
5dcy_A
5dds_A
5ded_A
5deq_B
5dgk_A
5dgk_A
5dgr_A
5dii_D
5dis_A
5dis_C
5dj1_B
5dj4_A
5djq_H
5djs_C
5dkj_V
5dku_B
5dle_D
5dll_A
5dm0_B
5dm3_C
5dm7_H
5dmh_A
5dmp_A
5dnd_D
5dnf_D
5dnf_G
5dnk_A
5dok_A
5dok_B
5dot_A
5dp2_A

5dpl_A
5dt9_A
5dte_B
5duq_A
5duv_D
5dv2_A
5dvi_B
5dwk_C
5dwn_A
5dx5_A
5dx9_A
5dxv_A
5dy1_A
5dy9_E
5dyx_A
5dze_A
5dzq_O
5e02_A
5e16_A
5e1q_A
5e1q_B
5e1r_E
5e2q_A
5e40_A
5e44_A
5e4w_D
5e68_A
5e6f_A
5e6f_A
5e72_A
5e79_I
5e7c_k
5e7g_A
5e7t_L
5e8j_B
5e98_A
5e9a_D
5e9h_A
5e9s_A
5e9s_B
5e9u_G
5ea2_C
5ea2_G
5eaf_B
5eb2_A
5ec2_C
5eck_E
5eck_F
5ecm_E
5ed8_A
5edl_A
5edu_B
5ee3_B
5eef_B

5eev_D
5eex_G
5eey_J
5eey_P
5eez_H
5ef0_F
5ef1_O
5ef8_B
5efv_A
5egj_A
5egp_A
5ehi_C
5eik_A
5ein_C
5eio_A
5eiy_A
5eiy_B
5eiy_B
5ej0_A
5ej6_B
5ej6_D
5eja_D
5ek8_A
5ekc_E
5eku_B
5el2_A
5eld_A
5elm_B
5em2_A
5emi_A
5emw_D
5enj_A
5eoh_A
5eom_C
5eow_A
5epo_A
5epv_A
5eq6_A
5eq8_A
5eq9_B
5eqb_A
5eqn_A
5er3_A
5er7_A
5erg_B
5erl_B
5erl_D
5erl_D
5es4_D
5esj_A
5esk_A
5ess_A
5esv_G
5esx_A

5euo_G
5eur_A
5evy_X
5ex1_C
5ex8_A
5exr_F
5ez2_B
5ez3_C
5ez7_A
5ezb_A
5ezu_A
5ezv_C
5f0p_B
5f11_A
5f1x_A
5f2k_A
5f2t_A
5f2v_S
5f4w_C
5f5n_A
5f5x_A
5f6b_A
5f74_A
5f7h_B
5f7j_E
5f7p_A
5f84_A
5f8e_A
5fa1_A
5fac_D
5fag_A
5fak_A
5fak_E
5fal_A
5faw_B
5fb3_C
5fc9_A
5fcy_A
5ff5_B
5fg3_A
5fg6_A
5fg9_G
5fg9_V
5fgf_L
5fgi_N
5fgn_A
5fhh_A
5fhi_A
5fj9_I
5fja_B
5fjn_A
5fjq_B
5fk0_B
5fk1_A

5flh_A
5flh_A
5flx_D
5fm8_B
5fmc_B
5fn0_A
5fnk_A
5fno_B
5fo5_A
5fo5_A
5foe_B
5fph_D
5fq0_A
5fq0_C
5fq6_M
5fq8_A
5fql_A
5fr1_B
5fr2_B
5frd_A
5frf_A
5frt_C
5fru_B
5frw_B
5fsc_A
5fsx_A
5ft0_A
5ftb_A
5ftf_A
5ftt_D
5ftt_G
5ftu_C
5ftu_D
5ftw_A
5fu2_B
5fub_A
5fue_D
5fv0_B
5fvj_A
5fwn_A
5fwt_A
5fyg_A
5fyo_A
5fzo_B
5g0a_B
5g0j_A
5g0v_B
5g0y_B
5g12_B
5g17_B
5g1f_B
5g2t_A
5g3u_A
5g49_B

5g4i_A
5g4l_B
5g50_A
5g54_A
5g5s_A
5g6s_B
5gaf_i
5gag_R
5gag_Y
5gg6_B
5ggx_D
5gif_A
5gii_A
5gio_M
5giv_A
5giv_C
5gja_A
5gjo_A
5gju_A
5gkq_B
5gl5_A
5glz_D
5gm2_B
5gmd_A
5gmf_A
5gmf_A
5gmk_F
5gnf_A
5gnr_A
5gny_D
5go5_A
5goo_A
5gox_A
5gp9_A
5gpo_B
5gpy_A
5gqo_A
5grb_B
5gre_A
5grk_B
5gs9_C
5gsm_B
5gss_B
5gt1_A
5gt7_C
5gt9_B
5gtd_A
5gti_v
5gtl_B
5gud_E
5gug_A
5gux_C
5gv2_C
5gvc_A

5gvd_B
5gvi_A
5gvw_C
5gvx_A
5gvy_B
5gwd_B
5gwe_A
5gwe_B
5gwt_C
5gx1_A
5gx3_A
5gx6_B
5gxe_A
5gy6_A
5gyd_C
5gyr_J
5gzk_B
5gzs_A
5h0o_A
5h1w_A
5h20_A
5h29_A
5h2d_A
5h2f_y
5h2j_A
5h40_B
5h4g_A
5h59_A
5h5f_A
5h5o_A
5h5x_A
5h5x_G
5h67_A
5h6h_A
5h6l_A
5h75_D
5h7k_A
5h86_A
5h9i_A
5h9o_A
5ha5_A
5hae_A
5hbs_A
5hc2_C
5hc8_A
5hc9_A
5hcc_A
5hcf_B
5hcn_A
5hdi_A
5hdj_B
5hdq_A
5hes_A
5hev_A

5hev_B
5hev_C
5hfi_A
5hfj_C
5hfj_E
5hfs_A
5hg0_B
5hgq_D
5hgz_A
5hh9_A
5hha_B
5hi6_A
5hi8_B
5hjd_K
5hjm_A
5hjq_A
5hkj_A
5hkk_G
5hkv_B
5hkv_K
5hkv_Z
5hl7_O
5hli_A
5hm7_A
5hma_A
5hmn_B
5hnm_B
5hnu_A
5ho1_A
5hob_F
5hp8_C
5hqw_A
5hqx_A
5hs3_C
5hsa_F
5hsg_A
5hsi_B
5hsj_A
5ht7_A
5huq_B
5huv_A
5hux_A
5hv1_A
5hve_A
5hw4_A
5hwe_A
5hwn_B
5hwo_A
5hws_C
5hxh_B
5hy5_A
5hyb_A
5hzd_A
5hzi_A

5i0j_A
5i0k_A
5i0m_B
5i1w_A
5i26_C
5i29_A
5i4e_A
5i56_A
5i5b_A
5i5g_A
5i6c_A
5i7f_G
5i7v_B
5i89_A
5i8q_B
5i8x_C
5i93_A
5i9k_A
5iaa_A
5ib0_B
5ib5_D
5ibq_A
5icu_A
5idm_A
5idu_C
5idy_B
5ie3_B
5ie9_C
5ien_A
5if9_A
5ifk_A
5ifl_I
5ifl_K
5ifm_C
5ig2_B
5igj_A
5ihk_A
5iiq_A
5iit_C
5ij6_A
5ijg_B
5ijj_A
5ijw_B
5iki_B
5ikj_A
5iky_A
5ilo_B
5ini_F
5inj_A
5ion_A
5iov_B
5iov_C
5ip9_J
5ipp_A

5irm_C
5irr_B
5is2_A
5isp_X
5isx_A
5it1_C
5it9_b
5it9_N
5it9_X
5itv_C
5itv_D
5itz_B
5iun_D
5iun_E
5iw8_B
5iwa_S
5iwc_B
5iwl_A
5iwq_A
5iwx_A
5ixo_A
5iy5_B
5iz3_A
5iz4_A
5izd_A
5izk_A
5izl_A
5j0a_B
5j1j_A
5j1m_A
5j1s_B
5j33_G
5j3t_B
5j4e_B
5j4n_A
5j4y_A
5j5k_A
5j5l_A
5j5r_A
5j5t_A
5j60_D
5j62_B
5j67_C
5j6f_A
5j6p_B
5j6s_A
5j7a_A
5j7i_C
5j7k_B
5j7k_B
5j7m_B
5j7r_B
5j8t_A
5j9c_A

5j9w_E
5ja0_F
5jap_A
5jbd_B
5jbi_B
5jbx_B
5jc8_D
5jcz_C
5jdj_A
5jdx_A
5je1_A
5je8_B
5jes_B
5jfm_B
5jfo_C
5jh8_A
5jhs_Z
5jhy_A
5ji5_A
5jia_D
5jil_A
5jip_A
5jiz_A
5jj5_A
5jk4_A
5jla_D
5jmd_A
5jmq_A
5jn5_A
5jn9_C
5jnb_A
5jnb_A
5jne_A
5jnl_B
5jo2_A
5jo9_A
5jov_A
5joz_A
5jqt_A
5jrk_A
5jrz_A
5jsf_A
5jtj_A
5jug_A
5juy_D
5jv4_A
5jvg_3
5jvg_M
5jvm_B
5jw1_A
5jxm_A
5jxp_A
5jy1_B
5jy9_B

5jys_A
5jzv_A
5jzx_A
5k04_B
5k0n_B
5k16_B
5k1j_B
5k1r_A
5k1r_B
5k1v_A
5k21_A
5k21_B
5k27_A
5k2m_I
5k3w_A
5k5b_A
5k5t_A
5k5w_B
5k5z_D
5k75_D
5k7g_D
5k7h_A
5k7j_B
5k7u_A
5k82_B
5k8o_D
5k8z_C
5k99_C
5k9g_A
5kaf_h
5kaf_Y
5kag_D
5kag_I
5kai_K
5kay_A
5kay_B
5kbk_A
5kbw_B
5kc4_A
5kca_A
5kci_A
5kd2_A
5kd5_A
5kdj_A
5kds_A
5kdv_A
5ke1_B
5ker_B
5ket_A
5kh0_A
5kh4_A
5kha_A
5kha_B
5kiv_A

5kiw_A
5kiw_B
5kj5_D
5kj7_D
5kja_A
5kjd_B
5kl9_A
5klb_B
5klk_D
5klm_A
5klm_D
5klv_E
5kmd_D
5kmq_B
5kn5_C
5knu_A
5koy_A
5kp7_B
5kpf_B
5kq8_A
5kqu_C
5kqw_A
5kr6_B
5ks1_A
5ks8_F
5ksa_A
5kt0_A
5ktk_A
5kvc_A
5kvp_A
5kwk_B
5kx5_F
5ky2_A
5ky5_A
5ky7_A
5kyn_A
5kyo_E
5kyo_F
5kyx_B
5kzn_A
5l02_B
5l0z_A
5l12_C
5l1t_A
5l26_A
5l2r_A
5l3d_A
5l3w_A
5l3z_A
5l42_B
5l54_G
5l5a_L
5l5i_G
5l5p_b

5l5r_N
5l5u_A
5l5w_M
5l5w_M
5l62_Z
5l64_J
5l67_Y
5l6h_C
5l6k_A
5l7u_B
5l89_d
5l8a_A
5l8b_D
5l8q_C
5l95_B
5l9o_B
5l9w_b
5la9_A
5laa_A
5laz_A
5lc5_P
5lc5_R
5lcb_A
5lcd_D
5lcp_A
5lcx_A
5ld9_A
5ldq_C
5ldq_D
5ldw_P
5ldw_R
5ldx_P
5le5_K
5lex_J
5lf0_L
5lf2_B
5lf4_K
5lfu_A
5lfz_A
5lhd_C
5lhs_A
5lhs_A
5lhu_A
5lie_B
5liv_A
5lj3_V
5lj5_C
5ljt_A
5ljw_A
5lk9_B
5lkd_A
5lkf_A
5lkk_A
5ll9_A

5lla_A
5llb_B
5llt_A
5llw_B
5llz_A
5lm9_A
5lms_W
5ln4_C
5ln5_A
5lne_B
5lnk_J
5lnk_M
5lnk_X
5loe_A
5log_B
5lok_E
5lor_A
5los_A
5lpg_A
5lq4_B
5lrg_C
5lrr_B
5ls0_B
5ls0_B
5lsf_A
5lsf_B
5lsq_A
5lst_A
5lt9_B
5lth_A
5ltm_A
5ltw_L
5luf_B
5luf_d
5luf_E
5lug_B
5lv0_A
5lvo_A
5lvp_B
5lvs_A
5lvv_A
5lw3_A
5lwe_A
5lwk_B
5lxg_A
5ly0_A
5lyv_B
5lzg_A
5m0i_C
5m0p_A
5m1e_A
5m1p_B
5m21_H
5m2f_X

5m2s_A
5m41_A
5m45_F
5m4k_A
5m4o_F
5m54_B
5m6d_A
5m78_A
5m7f_A
5m7h_A
5m7o_A
5m86_J
5m8b_A
5m99_A
5m9n_B
5mau_B
5mbx_A
5mcl_A
5med_A
5mek_A
5mf1_A
5mfd_F
5mfe_B
5mg5_Q
5mg5_T
5mgu_A
5mgy_H
5mgz_A
5mi4_A
5mio_C
5mj7_A
5mkc_E
5mkf_B
5mki_C
5mki_E
5mkw_A
5mlv_D
5mlv_F
5mm1_A
5mm7_K
5mmi_2
5mmi_4
5mmi_6
5mmi_7
5mmi_E
5mmi_H
5mmi_P
5mmi_R
5mmi_T
5mmi_U
5mmi_X
5mmi_Y
5mmj_x
5mmj_x

5mmx_A
5mnn_A
5mns_E
5mob_A
5mor_A
5mox_A
5mpt_A
5mq5_B
5mqp_G
5mqt_A
5mr6_T
5mrl_A
5ms9_A
5msl_A
5msr_B
5mss_A
5msz_A
5mt3_L
5mtd_B
5mtw_A
5mua_B
5mul_A
5mv8_A
5mva_A
5mvy_H
5mw9_A
5mwk_A
5mx0_B
5mx1_A
5mx2_m
5mx6_D
5mz2_I
5mza_B
5mzx_D
5n05_A
5n07_A
5n0c_A
5n0e_A
5n0s_B
5n16_D
5n1e_A
5n1o_A
5n1p_D
5n1s_A
5n1t_W
5n2i_A
5n2j_B
5n2n_A
5n5d_A
5n5s_C
5n5y_R
5n60_Q
5n62_B
5n6n_C

5n6u_A
5n6y_A
5n77_D
5n7y_A
5n80_A
5n9u_A
5na4_A
5na7_A
5nb8_B
5nbd_B
5nbp_B
5nc3_A
5nc8_A
5ncc_D
5ncj_A
5ncx_A
5nd7_C
5ndc_B
5ne2_B
5neu_1
5nf3_A
5nfj_C
5nfm_A
5nfn_C
5nfq_A
5ng3_A
5ng6_C
5ng6_E
5ng7_B
5ngl_B
5ni6_A
5nii_B
5nja_A
5njf_C
5nkk_D
5nl9_B
5nli_B
5nm8_A
5nmi_Q
5nmp_B
5nmp_I
5nmw_A
5nno_A
5no2_N
5no2_N
5no2_T
5no3_P
5no3_T
5no9_B
5nqa_A
5nrd_A
5nrg_C
5nrh_B
5nsf_B

5nsw_C
5nt4_B
5ntt_A
5nuf_B
5nul_A
5nut_A
5nv1_A
5nvo_A
5nx5_A
5nxa_H
5nxb_B
5nxm_A
5nxr_A
5nxr_A
5nyn_A
5nz2_A
5nzl_A
5nzz_D
5o0x_A
5o1e_B
5o1o_A
5o2z_B
5o3n_A
5o3w_B
5o4j_A
5o4n_A
5o5c_D
5o5j_F
5o5z_B
5o60_F
5o60_g
5o60_Y
5o6y_B
5o7x_A
5o7x_D
5o7x_J
5o7y_A
5o80_A
5o8e_A
5o8p_B
5o8z_B
5o96_D
5o9b_A
5o9b_A
5o9x_A
5oa4_A
5oat_F
5oay_A
5oba_L
5oba_R
5obu_A
5oby_A
5oc1_A
5oc2_B

5oc3_A
5oc7_D
5oce_A
5ocs_C
5od1_A
5odc_D
5odh_J
5odi_F
5odi_L
5odj_A
5odo_B
5odr_H
5odu_E
5oea_B
5oed_A
5oed_B
5oet_B
5oet_B
5of3_C
5ofx_E
5oh1_A
5ohe_F
5ohf_G
5oie_A
5oj7_A
5oji_A
5ok8_B
5oke_C
5okz_g
5okz_O
5ol7_A
5olc_F
5olq_C
5olt_A
5on0_A
5on5_B
5onj_A
5onm_A
5oo4_A
5ooh_A
5ool_I
5ool_T
5oom_g
5oom_v
5oom_W
5opf_A
5opl_A
5opz_A
5oqt_A
5oqt_C
5ot1_A
5ot3_A
5owc_B
5ows_B

5oxf_A
5oxf_C
5oyj_C
5pgm_E
5pgu_E
5pgw_A
5q28_A
5q2k_A
5q33_A
5q35_A
5q37_A
5q38_A
5q3b_A
5q3s_A
5q41_A
5q4c_A
5q5c_A
5q5e_A
5q5m_A
5q5q_A
5q5z_A
5q6f_A
5q6i_A
5q6v_A
5q75_A
5q77_A
5q84_A
5q8d_A
5q9k_A
5q9m_A
5qqq_B
5qqx_B
5qrc_B
5qt3_A
5r99_A
5r9b_A
5rag_B
5raq_B
5rav_A
5rax_B
5rx0_A
5rx0_A
5std_C
5sv0_A
5svb_B
5svc_A
5svc_C
5svk_B
5svv_D
5swc_B
5swg_B
5sy1_B
5sy4_B
5syd_B

5syt_A
5sz3_A
5t0h_C
5t0j_A
5t0j_F
5t0w_A
5t1a_A
5t25_A
5t2v_A
5t38_A
5t3v_B
5t41_A
5t4o_B
5t4q_A
5t5m_B
5t5m_C
5t5q_B
5t5q_D
5t69_A
5t6c_A
5t7e_D
5t8u_B
5t8w_A
5t96_B
5t9x_A
5ta6_A
5ta8_A
5tar_A
5tbx_A
5tcs_B
5tcx_A
5tdf_B
5te8_B
5tea_B
5tem_A
5tes_B
5tew_A
5tex_A
5tg6_A
5tgc_D
5tgf_C
5tgq_A
5thj_A
5thk_B
5thp_L
5thq_A
5thq_C
5tht_A
5thz_B
5ti1_A
5ti1_H
5ti8_A
5tip_C
5tir_A

5tis_V
5tis_v
5tk5_A
5tkf_A
5tkh_A
5tl7_D
5tlb_A
5tls_A
5tmb_A
5tme_A
5tnv_A
5tnx_A
5tou_C
5tpv_B
5tq2_A
5trl_A
5ts2_C
5ts3_A
5ts3_B
5tsd_B
5tt8_F
5ttk_C
5tu5_A
5tue_A
5tvg_G
5tvn_A
5tvo_A
5tvq_A
5tvu_A
5tvx_B
5tw7_F
5twj_B
5tx6_A
5txk_A
5txk_A
5txr_A
5txu_A
5txv_G
5txv_J
5ty8_A
5tzb_E
5tzj_C
5u02_A
5u0m_A
5u1a_L
5u1e_A
5u1p_A
5u27_A
5u4q_A
5u4s_B
5u51_C
5u58_A
5u5g_A
5u5n_B

5u6p_A
5u6z_A
5u7w_A
5u7x_F
5u9e_A
5u9l_A
5u9p_B
5u9z_B
5u9z_B
5uag_I
5uam_A
5uam_A
5uao_A
5uav_D
5ub6_B
5uba_A
5ucd_A
5ucm_B
5ucv_A
5ucw_B
5ud5_B
5udu_A
5ufd_A
5ufe_B
5ufl_A
5ufn_A
5ufv_A
5ugr_A
5uhs_B
5uid_A
5uin_A
5uio_C
5uis_C
5uj5_A
5ujc_A
5ujm_E
5ujp_A
5uju_A
5ujv_A
5uli_A
5ulj_A
5ume_E
5umf_C
5umw_F
5un7_A
5uof_B
5uph_B
5uqi_A
5uqk_A
5uqp_B
5uqr_A
5uqr_B
5uqt_A
5uqz_A

5urr_H
5urs_C
5us1_C
5uu3_B
5uv4_A
5uve_B
5uve_H
5uvg_A
5uwc_G
5uwx_D
5uwz_A
5ux7_B
5ux9_A
5uxe_B
5uyy_C
5uz4_Z
5uzk_A
5uzs_D
5uzu_A
5uzx_A
5v0f_A
5v0s_B
5v2c_o
5v2p_A
5v2w_B
5v3u_A
5v4b_B
5v4t_B
5v57_A
5v69_A
5v6j_B
5v7m_A
5v7p_A
5v7y_A
5v8s_B
5v8t_B
5v96_D
5va8_D
5vao_D
5vbl_B
5vcb_D
5vcb_f
5vcm_B
5vcv_A
5vdf_A
5vdf_E
5vdq_B
5vdr_A
5vdw_A
5ve4_A
5veg_B
5ver_B
5veu_H
5vf0_B

5vg1_A
5vg1_A
5vg1_B
5vg2_C
5vgr_A
5vgz_c
5vhd_D
5via_A
5vie_C
5vj0_D
5vj1_L
5vju_A
5vk4_B
5vki_B
5vko_B
5vkw_A
5vkw_A
5vkx_A
5vm8_B
5vmq_C
5vmt_H
5vn2_C
5vn2_D
5vn4_B
5vn5_C
5vn6_A
5vng_A
5vol_C
5vop_B
5vp3_A
5vp3_A
5vpl_A
5vpn_E
5vqa_A
5vqb_C
5vqh_A
5vrh_A
5vsv_B
5vt1_A
5vug_A
5vww_A
5vx0_C
5vyf_F
5vyq_B
5vyt_B
5vyt_C
5vz0_C
5vzt_B
5w0o_A
5w0p_A
5w10_A
5w15_D
5w1m_Q
5w21_A

5w23_C
5w23_C
5w3v_A
5w45_A
5w4a_D
5w4y_A
5w57_B
5w58_A
5w5r_U
5w6h_A
5w6y_A
5w71_A
5w7k_A
5w7l_A
5w7p_A
5w8a_A
5w8u_C
5w8x_A
5wa0_D
5wai_B
5wam_B
5wat_A
5wau_I
5way_A
5wb2_B
5wba_A
5wbg_C
5wbg_E
5wch_A
5wci_A
5wcj_A
5wcn_M
5wda_D
5we0_A
5we2_B
5we8_B
5wee_C
5weo_C
5wew_A
5wfg_E
5whg_A
5whm_A
5whv_J
5whx_B
5wid_A
5wjd_A
5wjs_B
5wk0_A
5wk4_F
5wk7_A
5wm3_A
5wml_A
5wnr_I
5wnv_T

5wo6_A
5wob_C
5woo_A
5wpk_B
5wq0_D
5wqh_E
5wqo_A
5ws5_k
5ws8_D
5wt3_A
5wtq_A
5wtz_A
5wu2_A
5wud_A
5wuq_C
5wve_F
5wve_N
5wvp_A
5wvu_A
5wvx_A
5wws_A
5wwv_A
5wwx_A
5wxb_A
5wxu_B
5wz1_C
5wzv_A
5x03_B
5x0b_A
5x13_A
5x1f_S
5x1h_Q
5x1t_A
5x20_C
5x2a_B
5x2e_A
5x2m_L
5x2n_B
5x2n_C
5x2r_C
5x2r_K
5x2s_J
5x2u_C
5x37_A
5x39_A
5x3t_H
5x40_B
5x42_A
5x4a_A
5x4z_X
5x51_J
5x57_A
5x62_B
5x66_D

5x6a_B
5x6c_B
5x71_A
5x7e_A
5x7f_A
5x9r_A
5x9u_B
5x9v_D
5xaf_E
5xau_A
5xbd_A
5xbt_A
5xbv_A
5xco_A
5xd6_B
5xd8_B
5xde_C
5xdh_A
5xdq_P
5xe5_A
5xf9_C
5xfa_A
5xfm_A
5xg3_B
5xg4_U
5xgf_A
5xgu_A
5xj6_A
5xk9_C
5xkd_D
5xki_B
5xko_A
5xkr_B
5xkr_D
5xkx_A
5xls_A
5xlx_A
5xlx_D
5xmi_C
5xmo_A
5xmz_A
5xn8_A
5xnc_B
5xnf_A
5xnj_A
5xnl_z
5xnl_Z
5xnm_t
5xnr_A
5xoi_A
5xoy_A
5xp5_A
5xqg_H
5xqj_F

5xr2_C
5xr3_A
5xs8_B
5xs8_D
5xss_A
5xt2_C
5xtc_n
5xtd_W
5xte_G
5xtg_B
5xtk_A
5xtr_D
5xts_A
5xtu_A
5xu1_B
5xuh_C
5xuk_A
5xv0_F
5xvh_A
5xvs_B
5xwj_B
5xwq_A
5xxm_A
5xxo_B
5xxs_B
5xxt_H
5xxv_M
5xxx_N
5xyn_D
5y06_A
5y0n_B
5y2f_A
5y2v_B
5y31_D
5y41_A
5y4c_A
5y4f_B
5y4h_A
5y5e_A
5y5q_B
5y5s_T
5y5s_V
5y66_A
5y79_A
5y88_A
5y88_N
5y8l_A
5y8v_A
5y8x_A
5y8x_A
5y9d_B
5y9m_A
5y9r_A
5y9x_A

5ya1_A
5ya6_B
5ybl_B
5ybz_C
5yci_B
5ycr_C
5yd0_B
5yf0_A
5yfv_B
5yg5_B
5ygk_A
5ygq_A
5yh1_A
5yh8_A
5yho_A
5yhr_A
5yhv_D
5yij_A
5yix_B
5yiz_h
5yj7_C
5yja_C
5yjd_A
5yjl_B
5yk0_A
5ylb_A
5yn8_A
5yo9_B
5yoe_A
5yot_A
5yox_A
5ypw_E
5ypw_F
5yq7_R
5yqb_A
5yql_A
5yqw_A
5yrt_G
5ys0_B
5ys9_A
5ysc_A
5ysf_A
5ysm_A
5ysn_B
5ysq_B
5ysr_B
5ysr_D
5yt7_D
5ytk_A
5ytk_E
5yud_A
5yvm_A
5yw0_A
5yx4_A

5yy2_A
5yym_A
5z01_A
5z06_B
5z0d_B
5z0d_B
5z0q_C
5z16_B
5z1b_A
5z1b_B
5z1q_C
5z1z_A
5z21_B
5z25_A
5z2f_A
5z34_A
5z3f_A
5z3q_H
5z43_A
5z43_B
5z48_B
5z49_A
5z6e_A
5z79_F
5z8k_B
5z8t_C
5z8y_A
5z9j_C
5z9s_A
5zb3_B
5zba_A
5zbf_A
5zbj_A
5zcm_A
5zcr_B
5zcr_B
5zct_B
5zct_C
5zct_E
5zct_F
5zed_B
5zer_A
5zi5_A
5zie_B
5ziq_A
5ziq_B
5zj9_D
5zjg_D
5zji_H
5zjk_A
5zlp_D
5zm5_A
5zmm_A
5zmp_A

5zmu_D
5zmy_F
5zn7_D
5znq_B
5znt_A
5zov_A
5zov_A
5zow_B
5zpg_A
5zpk_B
5zpm_A
5zps_A
5zq0_A
5zqr_A
5zqs_A
5zqv_C
5zri_A
5zrl_B
5zrt_B
5zs6_A
5zu2_C
5zum_B
5zvg_A
5zvx_A
5zw4_A
5zw7_A
5zwa_B
5zwb_B
5zxm_B
5zy6_B
5zyr_B
5zzu_A
5zzu_C
5zzw_A
6a17_A
6a1w_A
6a23_A
6a2p_B
6a3g_B
6a3i_C
6a3j_C
6a3l_A
6a4k_C
6a4r_A
6a4x_A
6a6e_D
6a6m_A
6a71_A
6a72_B
6a7a_A
6a7c_A
6a7i_A
6a7t_B
6a83_A

6a87_A
6a8c_B
6a8i_A
6a8s_A
6a9a_A
6aal_A
6ac9_A
6ach_A
6ach_C
6acx_A
6adi_A
6adq_H
6adq_P
6aef_A
6agb_K
6agt_A
6ah3_K
6ah5_C
6ahr_K
6ai0_B
6ai0_B
6aii_A
6ain_B
6ait_B
6ait_C
6ajn_A
6ajv_A
6akv_A
6akw_A
6akz_A
6al9_A
6ala_C
6alj_C
6alp_A
6alr_A
6am0_A
6ang_A
6ao7_A
6aoj_A
6aoj_A
6apg_A
6apl_C
6apl_F
6aps_B
6aq4_A
6aq4_B
6aqh_B
6aqr_E
6aqz_A
6arv_A
6asl_A
6asr_B
6asy_B
6at3_A

6at7_A
6at7_B
6atg_B
6awn_A
6ax6_B
6ay4_A
6azq_G
6b00_A
6b05_A
6b0j_B
6b1z_A
6b2m_A
6b2w_A
6b39_A
6b4e_A
6b4e_D
6b4i_F
6b4j_F
6b53_B
6b5e_E
6b6u_A
6b80_B
6b82_A
6b8s_B
6b8w_B
6b92_A
6b9o_B
6b9r_C
6b9s_G
6b9t_D
6b9u_A
6ba1_P
6bax_C
6bax_D
6bb2_E
6bb2_G
6bbm_W
6bbr_A
6bbw_A
6bc3_A
6bcc_A
6bcq_D
6bd4_A
6bdd_A
6be1_E
6be6_D
6bea_A
6bff_B
6bfg_A
6bfz_A
6bgd_A
6bgv_A
6bhp_C
6bhr_G

6bht_C
6bia_B
6bk8_O
6bka_A
6bky_A
6blb_A
6bm6_A
6bmc_B
6bme_A
6bn1_B
6bnf_A
6bnn_A
6bq2_A
6bq2_B
6bqj_B
6bqv_D
6br7_B
6brg_C
6brh_B
6brl_A
6bs5_A
6bsu_B
6bt9_B
6btl_A
6btm_E
6bxb_B
6bys_A
6bzt_C
6c08_F
6c0d_A
6c0d_A
6c0f_D
6c0f_u
6c0m_B
6c10_A
6c1w_B
6c2z_A
6c32_A
6c3p_C
6c46_D
6c4m_C
6c4s_B
6c5b_A
6c5c_A
6c5w_A
6c62_B
6c6k_B
6c6r_A
6c71_C
6c7p_B
6c8q_A
6c8r_A
6c94_A
6c95_A

6c9e_B
6c9j_A
6ca4_C
6cap_F
6caq_T
6cb1_j
6cb7_A
6cb8_A
6cbt_B
6cc7_A
6cci_A
6ccy_A
6cdb_A
6cdr_A
6cdz_A
6ceq_C
6cer_A
6ces_A
6cfo_A
6cgi_A
6cgk_A
6cgn_A
6che_A
6ci7_B
6ci9_D
6ci9_P
6cia_A
6ciz_C
6cja_D
6cjt_A
6cjy_A
6cki_A
6ckl_C
6cko_C
6cmj_A
6cng_A
6cnl_K
6cpa_A
6cpd_A
6crn_B
6csf_M
6csl_A
6csm_D
6cso_A
6cth_A
6cv8_A
6cvf_B
6cvz_A
6cwz_D
6cx6_B
6cy8_A
6cy9_A
6cyp_D
6cyt_A

6czp_E
6czp_G
6czz_C
6d04_A
6d0g_A
6d0g_A
6d0o_D
6d0p_C
6d0v_B
6d0y_C
6d14_B
6d24_B
6d2v_A
6d2y_A
6d31_A
6d3a_A
6d3a_C
6d3u_B
6d3w_B
6d42_B
6d4k_A
6d4q_A
6d50_A
6d50_B
6d50_B
6d57_A
6d57_B
6d5b_E
6d5x_B
6d6n_C
6d6w_C
6d6y_A
6d72_C
6d7j_A
6d8d_A
6d8d_C
6d97_D
6d9y_B
6da0_A
6da6_A
6dam_A
6dcb_A
6dcd_A
6ddh_A
6ddr_C
6ddt_A
6det_A
6dex_A
6dex_B
6dft_A
6dft_F
6dg8_E
6dgi_A
6dhw_A

6dio_C
6djm_D
6djt_A
6dk9_D
6dk9_I
6dka_F
6dka_G
6dkd_A
6dkh_D
6dll_B
6dlu_P
6dlw_B
6dlw_V
6dmf_B
6dpr_A
6dq8_A
6dqy_Q
6drd_L
6drh_C
6drs_A
6dru_A
6ds2_H
6dtq_C
6du7_D
6du7_F
6duk_B
6dvh_B
6dvv_B
6dwo_C
6dwv_B
6dx3_D
6dx3_D
6dxl_B
6dxp_A
6dxs_B
6dy1_B
6dyc_A
6dym_A
6dzg_C
6dzs_A
6e0l_A
6e0o_A
6e0y_A
6e0z_A
6e0z_C
6e2o_A
6e2u_A
6e2y_A
6e36_B
6e3a_A
6e3l_I
6e40_B
6e48_F
6e70_B

6e88_L
6e88_N
6e8i_A
6e9f_B
6eax_A
6eb3_D
6ebk_E
6ebq_A
6ebv_C
6ec3_C
6eci_C
6eci_O
6eck_B
6ecv_A
6ed8_A
6edd_B
6edw_B
6edz_D
6ee2_C
6eeh_A
6eem_B
6ef3_L
6efx_A
6eg5_E
6egf_B
6egp_A
6egp_A
6ehf_A
6ei9_B
6eiq_A
6ejj_A
6ejx_A
6ejx_D
6eki_B
6el2_B
6elc_A
6elk_A
6elv_A
6elv_A
6em0_A
6emn_A
6emv_A
6enn_B
6enz_A
6eo0_B
6eo5_A
6eoa_A
6eop_A
6ep5_B
6ep5_F
6ep6_B
6eqi_C
6eqo_A
6esb_2

6esd_B
6esq_D
6esq_F
6et7_A
6et9_D
6eu4_A
6eu9_D
6euh_C
6euo_A
6euo_C
6euv_C
6eve_C
6ewq_A
6ewq_C
6ex0_B
6ex6_A
6exn_M
6eyc_7
6eyg_A
6eyp_C
6eyu_B
6eza_A
6f03_A
6f0b_A
6f0k_E
6f1a_A
6f1t_C
6f28_A
6f2f_C
6f2w_B
6f3a_G
6f3b_B
6f3h_B
6f3i_A
6f3p_C
6f45_B
6f4d_A
6f4k_B
6f4p_A
6f4p_A
6f5d_E
6f5z_B
6f67_B
6f6j_B
6f6j_C
6f70_A
6f73_A
6f74_B
6f77_F
6f7b_A
6f7t_L
6f7v_B
6f8y_A
6f8z_A

6f91_C
6f92_A
6fam_A
6fat_A
6fat_B
6fc2_A
6fc3_B
6fcg_C
6fd9_A
6fdf_A
6fea_C
6ff1_A
6ff4_t
6ff7_B
6ffv_A
6fgx_A
6fh1_A
6fhb_A
6fhg_A
6fhh_B
6fhi_A
6fho_A
6fhs_C
6fhs_J
6fht_B
6fii_F
6fij_B
6fjg_A
6fjg_B
6fju_A
6fjw_A
6fku_A
6flg_B
6fnn_D
6fnq_A
6fnu_A
6fny_A
6fny_E
6fok_A
6fol_A
6fol_G
6fon_C
6fos_I
6fp5_B
6fp6_V
6fpf_A
6fpp_B
6fpy_A
6fqb_E
6fqb_G
6frm_A
6fsl_A
6ftt_A
6fu1_B

6fu4_A
6fu7_B
6fuu_A
6fux_A
6fuy_A
6fv4_B
6fv5_B
6fvj_A
6fwh_E
6fyb_B
6fyq_A
6fze_A
6fzi_C
6fzv_A
6g0n_A
6g12_A
6g18_f
6g18_y
6g19_A
6g1b_J
6g1g_A
6g1h_A
6g22_A
6g2g_A
6g2n_A
6g33_A
6g3f_A
6g3y_A
6g40_A
6g55_A
6g5q_B
6g5z_B
6g63_N
6g6t_A
6g72_R
6g74_B
6g7f_I
6g7p_B
6g80_M
6g8m_Y
6g8u_A
6g90_U
6g96_A
6g99_B
6ga9_A
6gar_A
6gas_B
6gau_A
6gbx_B
6gck_A
6gcl_B
6gcp_B
6ge8_A
6gek_B

6geq_A
6gex_B
6gey_C
6gfe_H
6gfo_A
6gfr_B
6gg1_A
6gg1_A
6gg2_A
6ggf_B
6ggo_A
6gh1_G
6gh2_A
6ghb_D
6ghl_A
6ghs_A
6gi2_B
6gi5_B
6gio_C
6giq_O
6giw_D
6gku_A
6gky_A
6gl0_B
6gl0_B
6gmh_A
6gmo_A
6gn6_D
6gn6_F
6gnc_A
6gne_B
6gng_B
6go1_B
6goc_A
6gop_L
6gos_1
6gp3_A
6gpa_A
6gqd_A
6grh_C
6gs8_F
6gtm_C
6gur_B
6gvw_B
6gw6_D
6gwf_A
6gwu_A
6gwu_B
6gxu_B
6gxz_A
6gyx_B
6gzh_A
6gzo_B
6gzt_A

6gzu_A
6h08_B
6h0f_I
6h0g_E
6h17_A
6h17_A
6h1e_A
6h1t_B
6h2t_A
6h2v_A
6h39_X
6h3d_A
6h3o_F
6h3o_H
6h42_A
6h4c_B
6h4c_H
6h4l_A
6h56_A
6h57_A
6h5t_B
6h65_C
6h7d_A
6h7e_A
6h7e_B
6h7f_A
6h7f_C
6h8n_A
6h9b_B
6h9f_C
6h9h_B
6h9v_A
6has_A
6hau_B
6hbv_B
6hbw_C
6hcz_B
6hd0_A
6hdb_B
6hde_B
6hdp_A
6hdt_A
6hea_H
6heg_A
6hf3_A
6hf4_A
6hf7_B
6hft_A
6hft_A
6hg8_B
6hgb_A
6hhb_B
6hhm_A
6hik_A

6hip_B
6hit_D
6hiu_A
6hj3_C
6hjd_A
6hk5_B
6hk7_A
6hlm_C
6hls_J
6hmj_A
6hnd_B
6hni_A
6hnq_B
6hp2_A
6hpd_A
6hph_A
6hq3_B
6hqd_B
6hqm_A
6hqn_A
6hr5_A
6hrc_C
6hrk_B
6hsd_A
6htd_Z
6htg_B
6htj_A
6htl_A
6htr_N
6htr_Y
6hu0_C
6hu1_B
6hu2_C
6hu9_L
6hu9_p
6huc_N
6huc_V
6hul_A
6hun_A
6huu_K
6huu_L
6hux_A
6hv7_N
6hv7_W
6hvg_A
6hvl_B
6hvs_Y
6hvu_V
6hvw_L
6hvx_V
6hw2_B
6hw2_B
6hw3_K
6hwd_W

6hwh_A
6hxp_A
6hxq_A
6hy1_C
6hy3_A
6hyc_B
6hyj_A
6hyy_B
6hze_A
6hzn_A
6i02_B
6i1j_A
6i1r_B
6i25_A
6i28_A
6i2x_B
6i2z_A
6i2z_B
6i34_B
6i39_A
6i3v_B
6i3v_F
6i5f_B
6i5x_C
6i79_B
6i7g_B
6i7l_A
6i7s_B
6i8e_B
6i8f_A
6i8f_B
6i98_A
6i9k_A
6ia6_A
6iao_B
6iaq_D
6iau_B
6ib8_B
6ibb_C
6ibi_A
6ic2_A
6ici_A
6icn_A
6icz_A
6icz_Z
6id1_U
6idy_A
6ie0_C
6ie9_A
6ien_B
6ifc_A
6ifd_A
6ifi_B
6ift_A

6ify_A
6ig2_D
6igj_A
6igz_M
6ihd_B
6ihi_B
6iih_A
6iiy_A
6ij9_C
6ij9_D
6ijb_B
6ijm_A
6ik5_B
6ila_A
6ilm_A
6iln_A
6imc_C
6imc_D
6ime_B
6imv_A
6in7_B
6inh_A
6ioh_A
6iop_A
6iov_A
6iox_A
6iq1_B
6iqq_C
6iqq_D
6iqw_A
6iri_A
6irw_A
6isp_A
6ist_C
6itx_D
6iu5_H
6iuf_A
6iuq_B
6iv2_D
6iv9_A
6ivw_B
6iwo_A
6iwr_F
6ix7_B
6ixj_D
6ixj_I
6ixj_L
6ixt_C
6iy6_A
6iy8_D
6iym_B
6iyx_A
6izh_C
6j07_B

6j0p_A
6j0t_B
6j38_A
6j3f_B
6j3g_A
6j3h_A
6j44_A
6j53_B
6j55_A
6j5w_A
6j5x_A
6j61_A
6j66_B
6j6f_A
6j6h_A
6j6j_A
6j6j_D
6j6q_F
6j71_A
6j76_B
6j7c_A
6j7l_A
6j7u_C
6j7x_B
6j83_A
6j9t_F
6j9u_A
6j9v_A
6jai_A
6jau_A
6jb4_A
6jbj_B
6jd7_A
6jdd_A
6jde_B
6jdl_A
6jdo_A
6jeb_A
6jf4_B
6jf8_C
6jf9_B
6jfk_A
6jfm_B
6jhb_B
6jhu_A
6jif_A
6jix_A
6jiz_C
6jj7_A
6jkh_A
6jki_B
6jko_B
6jkp_D
6jks_B

6jks_D
6jku_A
6jl5_B
6jl9_A
6jl9_A
6jlj_z
6jlk_j
6jll_J
6jll_V
6jll_z
6jln_F
6jlo_v
6jlo_z
6jls_A
6jmq_B
6jmt_D
6jn7_A
6jo0_A
6jow_A
6jpb_F
6jpl_D
6jq0_C
6jq0_E
6jq8_A
6jqe_B
6jqh_B
6jqx_A
6jrq_A
6jrq_C
6jrq_D
6js9_A
6jsb_B
6jsc_B
6jsj_A
6jt1_B
6jt5_A
6jt6_A
6jtd_A
6jtt_A
6jtz_A
6ju7_B
6juh_A
6jv1_A
6jw8_A
6jxk_F
6jxn_D
6jy4_L
6jz5_A
6jz5_B
6jzy_A
6k02_B
6k0b_G
6k0k_A
6k0v_A

6k0v_A
6k15_H
6k1f_B
6k1g_D
6k1h_C
6k1r_A
6k28_A
6k2l_A
6k2m_E
6k37_A
6k3c_B
6k4c_A
6k4j_A
6k4r_B
6k61_x
6k73_B
6k7n_A
6k7y_H
6k7y_P
6k80_A
6k86_B
6k8d_A
6k8n_A
6k8p_A
6k8u_A
6k8w_A
6k96_B
6k9j_A
6kab_A
6kab_C
6kbe_G
6kby_A
6kcv_B
6kd5_A
6kdp_A
6kez_F
6kf4_G
6kf4_P
6kfn_A
6kfq_A
6kfs_A
6kfu_A
6kfw_A
6kg2_A
6kgy_C
6khj_D
6khx_B
6khx_G
6ki1_B
6kia_A
6kih_C
6kih_F
6kiy_A
6kji_A

6kko_A
6kko_C
6klc_A
6kls_D
6klv_F
6kn8_B
6kn8_D
6kn8_O
6kni_C
6kpm_A
6kqx_A
6krt_B
6ks0_A
6ksr_A
6kth_A
6ktk_C
6ktx_A
6ku6_B
6kuf_A
6kwa_A
6kwf_A
6kwx_A
6kwy_c
6kxk_D
6ky4_A
6kyf_A
6kyg_A
6kz8_A
6l1b_A
6l1l_B
6l1q_B
6l1u_O
6l2c_A
6l2c_C
6l2d_A
6l2f_B
6l2m_A
6l2z_B
6l3u_B
6l3u_C
6l3u_F
6l3w_A
6l42_A
6l47_A
6l4c_B
6l55_A
6l55_J
6l5m_E
6l69_B
6l6d_A
6l6w_A
6l7o_A
6l7o_B
6l7o_F

6l7o_P
6l7p_A
6l7w_A
6l8a_D
6l8h_D
6l8k_A
6l8u_C
6lai_B
6lb7_A
6lb9_A
6lcp_A
6leb_A
6lf6_A
6lfo_R
6lgj_C
6lgy_A
6lh9_A
6li0_A
6li1_A
6li2_A
6li6_A
6li9_B
6lid_C
6lj3_A
6lj3_B
6ljj_A
6lke_A
6lkj_A
6lkn_M
6lkr_B
6ll9_A
6llx_B
6lm0_C
6ln2_A
6loj_B
6lp1_C
6lrb_D
6lrg_B
6lth_L
6lum_E
6lvb_A
6lx2_A
6lx9_A
6lxy_B
6lyh_B
6lz1_A
6lz7_A
6lzg_A
6lzj_A
6m01_A
6m0a_B
6m0j_A
6m0r_B
6m0r_H

6m0r_O
6m17_A
6m18_D
6m20_D
6m35_H
6m3x_G
6m3x_U
6m48_A
6m4f_G
6m89_A
6m8n_A
6m8r_F
6m8t_A
6m8v_A
6m97_A
6m9b_A
6m9m_A
6m9r_A
6mal_A
6mb6_A
6mb9_C
6mbg_A
6mbj_A
6mbn_B
6mcp_C
6mde_A
6mdv_B
6me8_A
6me9_A
6meb_B
6mfd_A
6mfe_A
6mfl_A
6mfl_A
6mfv_C
6mgc_A
6mgj_F
6mj2_B
6mkk_A
6mkw_A
6mlb_D
6mlw_A
6mn5_D
6mni_A
6mo5_A
6moi_B
6moi_B
6mp5_B
6mp9_A
6mpb_B
6mpf_D
6mps_A
6ms2_A
6ms4_B

6mso_B
6mso_D
6mu0_A
6muk_A
6mur_D
6muu_A
6mvf_C
6mvs_A
6mw5_A
6mx5_A
6mxd_D
6mzb_A
6n04_B
6n0k_A
6n0v_B
6n1c_A
6n1f_D
6n1x_A
6n20_B
6n20_D
6n23_A
6n23_C
6n2b_B
6n2c_A
6n2m_B
6n2o_C
6n35_M
6n3d_A
6n3d_A
6n3r_C
6n54_B
6n63_A
6n6q_A
6n6q_B
6n7r_B
6n8b_A
6n8b_A
6n8e_A
6n91_B
6n94_A
6n96_D
6n9q_P
6nal_A
6nas_N
6naz_A
6nbe_N
6nbn_A
6nbq_K
6nci_B
6ncr_B
6ncs_B
6ncx_C
6ncy_A
6ndx_D

6neg_A
6nes_A
6nf4_A
6nff_A
6ng3_A
6nh5_D
6nh7_B
6nhg_E
6nhx_A
6nil_D
6nin_U
6njc_A
6njd_A
6njd_B
6njw_A
6nki_A
6nkm_F
6nkq_B
6nl5_C
6nlk_L
6nlp_B
6nlq_B
6nma_A
6nmi_B
6nmn_B
6no7_A
6nok_A
6nor_A
6nor_D
6noz_A
6np3_A
6npd_A
6npo_A
6npy_A
6nr0_A
6nro_A
6nrz_A
6ns3_A
6ns4_A
6nt2_C
6nt7_A
6nt8_A
6ntb_C
6nu8_A
6nu9_A
6nud_J
6nul_A
6nvy_A
6nw7_B
6nw9_A
6ny6_B
6ny6_D
6ny8_A
6nz0_Z

6nz0_Z
6nz4_A
6nzu_A
6o0g_A
6o2y_B
6o38_B
6o3v_B
6o49_A
6o4c_C
6o4k_B
6o4k_C
6o4k_F
6o58_A
6o5e_A
6o5y_D
6o6l_B
6o6o_A
6o77_D
6o7e_D
6o9g_D
6o9m_4
6ob0_A
6ob2_B
6ob5_D
6obd_A
6obg_C
6oby_B
6oc6_B
6odd_A
6oe2_A
6oec_L
6oew_B
6oex_A
6of3_B
6ofs_A
6ofu_B
6ogn_A
6oh2_A
6oh7_A
6oh9_A
6ohb_A
6ohh_B
6ohk_A
6ohp_A
6ohp_C
6oi2_A
6oid_B
6oio_A
6oja_B
6oja_F
6ojt_A
6ok0_D
6ok1_B
6ok2_D

6okk_T
6okv_D
6okz_B
6oln_C
6om5_A
6oms_A
6on3_F
6on4_A
6on5_A
6onq_A
6onx_A
6oo8_A
6op8_A
6oqa_D
6oqm_A
6or3_A
6org_B
6ori_A
6otf_B
6otj_B
6ott_B
6otu_A
6oty_B
6ous_A
6ouv_A
6ov6_A
6ow0_A
6ow4_F
6owj_A
6own_B
6owo_M
6ox4_B
6ox5_A
6oxd_A
6oyw_C
6oz7_A
6oz7_C
6oza_A
6ozr_B
6ozv_A
6p1z_E
6p1z_H
6p2a_C
6p2i_A
6p2t_A
6p3i_A
6p42_A
6p43_C
6p4v_A
6p4w_A
6p57_A
6p59_B
6p5r_A
6p5s_A

6p6j_A
6p7o_A
6p81_A
6p8f_C
6p8l_G
6p8o_A
6p8p_A
6p8u_A
6p8u_A
6p8v_C
6p9u_F
6pa2_A
6pa3_D
6pai_C
6pak_B
6par_B
6pbc_A
6pbm_A
6pbm_B
6pc1_A
6pcc_A
6pda_A
6pdc_A
6pe3_A
6pej_B
6pfa_C
6pfy_W
6pgl_A
6pgn_A
6ph2_C
6ph4_A
6pi5_D
6pi9_A
6pii_A
6pk3_B
6pk6_A
6pki_A
6pl0_B
6pml_B
6pmp_C
6pmu_B
6pnj_T
6po4_A
6po4_C
6pol_F
6pop_A
6pp8_F
6ppj_A
6pq9_A
6pqb_C
6pqn_A
6pqz_B
6pr4_A
6prm_D

6pro_A
6prx_B
6prz_A
6ps2_A
6ps3_A
6psd_E
6pso_A
6pt8_A
6pt9_B
6ptn_2
6ptn_3
6ptn_5
6pty_A
6puu_D
6pvg_A
6pvz_B
6pw3_A
6pw7_B
6pw8_A
6pwj_A
6pwq_B
6pxa_I
6pxa_J
6pxk_A
6pxk_K
6pxs_A
6py0_C
6pyz_A
6pza_C
6q05_C
6q21_A
6q2k_A
6q34_B
6q3x_A
6q56_D
6q67_A
6q72_D
6q7j_B
6q8n_A
6q9c_A
6qb8_Q
6qbt_A
6qbw_A
6qbw_A
6qci_B
6qcl_B
6qct_A
6qd7_E
6qe6_A
6qel_I
6qga_E
6qgb_A
6qgb_E
6qhd_B

6qhe_A
6qhn_D
6qip_B
6qix_A
6qj6_A
6qja_D
6qjc_D
6qk7_C
6qmm_B
6qn6_B
6qp2_B
6qp4_A
6qp4_A
6qpa_H
6qpa_P
6qpa_Q
6qpk_B
6qps_A
6qqi_A
6qqm_A
6qrj_A
6qro_B
6qro_D
6qsi_A
6qsk_E
6qta_A
6qu0_B
6qu2_A
6qug_C
6qul_b
6qul_b
6qul_f
6quy_G
6quz_D
6qv5_A
6qvj_S
6qvv_A
6qwj_R
6qxn_A
6qyi_B
6qz2_E
6qz4_A
6r02_C
6r0s_A
6r0s_B
6r0w_E
6r12_B
6r16_H
6r1a_A
6r1b_C
6r1g_B
6r1l_A
6r1x_C
6r26_A

6r3y_A
6r41_A
6r48_B
6r4l_A
6r4n_E
6r4p_B
6r52_A
6r5r_A
6r5u_A
6r62_A
6r6h_E
6r6m_B
6r6n_A
6r7p_A
6r81_B
6r88_D
6r8u_D
6ra9_B
6raf_A
6raj_B
6rak_A
6rb7_A
6rdk_M
6rdo_M
6rdx_M
6rec_M
6rez_C
6rf0_C
6rfa_A
6rfl_B
6rfl_I
6rfq_J
6rfq_X
6rfr_4
6rfr_j
6rfr_M
6rfr_Z
6rft_A
6rgs_B
6rgz_D
6rh8_B
6rhv_H
6ric_J
6riw_A
6rjm_B
6rjv_B
6rk9_B
6rkb_B
6rkh_A
6rkj_A
6rl5_D
6rl5_G
6rmd_D
6rmg_B

6rmp_A
6rmr_A
6rmr_A
6roh_A
6rqa_A
6rqf_D
6rqf_F
6rqf_J
6rr3_A
6rr9_A
6rr9_A
6rre_D
6rre_F
6rsw_B
6rum_A
6rur_B
6rur_H
6rve_A
6rvm_D
6rw0_A
6rx0_B
6rxb_C
6ry0_A
6ryf_A
6rzb_B
6s0g_B
6s0k_f
6s0r_B
6s0t_A
6s0v_B
6s1g_A
6s20_C
6s21_A
6s2e_A
6s2p_N
6s3t_B
6s4b_A
6s4d_A
6s58_C
6s5b_D
6s5g_D
6s5n_D
6s5n_E
6s61_O
6s6b_K
6s6v_C
6s6v_D
6s6y_D
6s6y_J
6s6z_B
6s7o_E
6s7r_N
6s7t_A
6s83_G

6s84_E
6s8f_H
6s8w_D
6s9o_F
6sac_A
6sag_A
6sau_A
6sc4_D
6scz_A
6sdu_A
6sdv_A
6sg8_B
6sgf_A
6sh3_D
6shj_C
6shj_D
6si6_A
6siu_C
6sj2_B
6sjd_A
6sl0_A
6sl1_A
6sl3_A
6sll_B
6smk_D
6smt_B
6smw_D
6smz_C
6sna_A
6sna_B
6sne_L
6sne_P
6snn_A
6so2_A
6sp2_E
6spi_C
6spk_A
6spr_A
6sps_A
6spv_A
6sq8_B
6sqg_C
6sqg_E
6sqi_A
6sqx_B
6srb_B
6srd_A
6srt_A
6ssd_A
6ssp_B
6ssy_A
6stu_A
6sty_B
6su3_X

6su6_A
6suk_A
6sv1_d
6sv1_M
6sv5_A
6svo_A
6sw9_R
6swc_F
6swc_W
6swl_A
6sy7_H
6syu_A
6sz4_A
6sz9_C
6szw_C
6t0e_B
6t0f_D
6t15_H
6t2b_B
6t2t_A
6t3v_A
6t4c_C
6t4v_C
6t5h_A
6t5w_A
6t69_A
6t70_A
6t7k_A
6t8l_A
6t8m_B
6t9n_D
6t9o_B
6t9o_D
6tas_H
6tbb_G
6tbx_C
6tc6_A
6tcb_B
6td3_H
6td7_A
6tdg_A
6tdv_E
6tdv_O
6tdv_P
6tdv_R
6teh_C
6tej_A
6tek_A
6tfk_A
6tfq_A
6tg9_C
6tgk_C
6th0_A
6tht_A

6tir_A
6tj6_B
6tj7_A
6tjv_P
6tk0_X
6tl0_B
6tl4_A
6tl5_A
6tl7_A
6tld_C
6tmf_Q
6tmw_B
6tmx_E
6tmx_M
6tnh_A
6tnh_B
6tnh_B
6tnm_A
6tnn_I
6tnn_p
6toe_B
6tou_G
6tp9_G
6tpq_q
6tpv_B
6tq5_A
6tqf_B
6tqm_B
6tr8_A
6trk_B
6trq_C
6tsh_D
6tsk_D
6tt1_A
6tt1_B
6ttl_B
6ttn_A
6tuk_B
6tv9_H
6twi_E
6twi_E
6twm_D
6twm_D
6twm_E
6tx8_A
6ty4_B
6tzk_A
6tzu_B
6tzw_A
6tzz_A
6u08_A
6u0p_C
6u19_B
6u1h_D

6u1q_A
6u1v_A
6u1v_B
6u1z_B
6u2a_A
6u2f_A
6u33_A
6u3b_B
6u3e_A
6u3g_A
6u3u_A
6u45_A
6u5i_A
6u5t_A
6u5v_B
6u6c_E
6u73_A
6u78_A
6u7b_A
6u7c_A
6u7g_A
6u83_A
6u87_A
6u8y_J
6u96_D
6u97_A
6u9d_G
6u9d_R
6u9d_T
6u9j_B
6u9k_B
6u9n_A
6uak_A
6uau_A
6uaz_B
6ubn_B
6ubn_B
6ubt_E
6ucm_C
6ucm_C
6ucy_B
6ud4_F
6ued_A
6ued_A
6ueg_B
6ueh_A
6uek_A
6ufo_A
6uh2_B
6uh7_A
6uhh_B
6uhx_B
6ui4_A
6ui5_B

6uj5_A
6ukl_E
6uks_B
6ulu_B
6umr_A
6umt_A
6un3_A
6un8_B
6uog_D
6upc_A
6upd_B
6uq9_B
6uqf_B
6uqk_A
6uqk_B
6uqm_A
6uqq_C
6ur8_B
6usm_B
6uss_B
6uss_B
6ust_C
6ut5_D
6ut6_A
6ut6_B
6ut6_D
6uts_A
6uuw_D
6uv0_A
6uv6_C
6uvb_A
6uwd_A
6uwy_B
6ux4_F
6uyh_B
6uyk_C
6v04_A
6v1r_A
6v26_D
6v2h_E
6v2h_E
6v2t_B
6v2v_A
6v30_B
6v35_C
6v35_D
6v35_F
6v3f_A
6v3t_D
6v3t_G
6v43_A
6v4k_H
6v4n_W
6v4s_E

6v55_A
6v63_A
6v6f_A
6v6h_A
6v77_B
6v7z_B
6v81_A
6v8p_A
6v8q_A
6v9s_A
6v9w_A
6v9w_B
6vby_A
6vcd_B
6vcf_A
6vd9_C
6vda_A
6vdc_A
6vdy_A
6vfy_F
6vg0_B
6vgc_D
6vgt_A
6vh5_D
6vhf_A
6vi3_E
6vig_D
6vii_B
6vim_C
6vim_H
6vji_A
6vlt_D
6vly_A
6vm0_A
6vm2_B
6vm5_A
6vmv_A
6vmw_B
6vny_A
6vo5_A
6vog_E
6voj_A
6voq_A
6vp8_A
6vpb_B
6vrg_A
6vri_D
6vsd_A
6vsp_B
6vtj_A
6vtv_A
6vtv_B
6vu8_A
6vu9_A

6vuy_A
6vv8_B
6vvd_B
6vvo_A
6vvo_B
6vvo_E
6vvq_C
6vw8_C
6vww_A
6vx6_E
6vx7_C
6vyo_B
6vz0_A
6vz6_A
6vz8_O
6w04_A
6w0s_A
6w0s_B
6w1h_A
6w1i_B
6w1k_A
6w1k_C
6w25_A
6w2l_A
6w2q_A
6w2x_A
6w32_B
6w3v_B
6w4e_B
6w4x_C
6w5v_D
6w6d_A
6w6y_A
6w6z_A
6w80_A
6wa8_A
6wb6_B
6wcd_A
6wct_D
6weg_A
6wek_B
6wf7_A
6wfk_C
6wgm_A
6whd_B
6wj2_G
6wj6_X
6wjj_J
6wjp_A
6wjv_A
6wlc_A
6wm2_0
6wm2_R
6wm4_A

6wmv_C
6wn0_B
6wn6_A
6wn6_C
6wn6_C
6wn9_A
6wng_A
6wo2_A
6wp5_C
6wpu_A
6wq3_A
6wql_A
6wqp_B
6wrf_C
6wrz_A
6wsg_E
6wt7_A
6wuk_A
6wv8_A
6wx6_B
6wxp_A
6wy9_A
6wyi_A
6wyy_D
6x0q_L
6x1g_C
6x3x_A
6x40_A
6x5r_A
6x5u_D
6x7l_A
6x8z_A
6x91_B
6x91_H
6x99_A
6x9a_B
6xby_A
6xig_B
6xji_C
6xk2_A
6xl1_A
6xmt_A
6xn0_B
6xpv_B
6xqn_B
6xr5_D
6xrk_A
6xs3_A
6xtv_B
6xty_2
6xty_4
6xty_5
6xty_6
6xty_6

6xu2_A
6xv4_A
6xvg_B
6xw7_B
6xwq_B
6xxj_A
6xyd_A
6xz8_B
6y04_B
6y2z_A
6y3z_P
6y41_B
6y41_C
6y43_A
6y4e_A
6y5a_A
6y5y_I
6y79_2
6y79_5
6y8j_A
6y9t_A
6ya6_A
6ya7_B
6yb8_B
6ybq_D
6ybw_L
6yc1_C
6yca_A
6ydc_D
6yes_A
6ygc_A
6ygd_A
6ygf_A
6ygg_A
6ygl_A
6yhv_B
6yj1_A
6yj4_a
6yj4_b
6yj4_D
6yj4_R
6ylq_A
6ymy_b
6ynv_e
6yoa_B
6yp7_W
6yp7_x
6yt3_B
6yu6_B
6yua_A
6yub_A
6yub_B
6yug_B
6yus_A

6yva_C
6yw2_A
6yw3_A
6yw7_A
6ywk_C
6ywp_A
6yxa_A
6yxs_A
6yyi_B
6yzm_A
6yzs_A
6z0v_S
6z0v_S
6z1f_1
6z1f_3
6z2k_F
6z2p_A
6z3b_A
6z3r_A
6z4w_A
6z4x_E
6z5a_A
6z69_B
6z80_F
6z96_A
6z96_B
6z9i_B
6z9k_B
6za7_A
6za9_N
6zaz_A
6zaz_B
6zb6_D
6zcw_A
6zdc_A
6ze9_A
6zgz_E
6zhk_A
6zi9_L
6zje_A
6zk9_2
6zke_4
6zkf_W
6zkh_s
6zkk_6
6zkk_x
6zkl_i
6zkl_Y
6zkm_4
6zkn_6
6zkp_o
6zkr_z
6zks_b
6zkv_N

6zkv_s
6zld_B
6zll_A
6zlw_c
6zlz_B
6znl_C
6znn_J
6zns_A
6zns_A
6zoj_T
6zok_L
6zol_d
6zol_F
6zpm_B
6zqm_b
6zqm_E
6ztq_O
6ztq_P
6zu8_A
6zvh_a
6zvh_a
6zvh_G
6zvh_O
6zwm_B
6zwo_F
6zxg_U
6zy3_H
6zy9_G
6zyd_A
6zyk_A
6zzp_A
6zzs_C
6zzs_D
6zzs_F
7a17_A
7a1g_B
7a1g_S
7a23_B
7a23_H
7a23_J
7a23_P
7a23_T
7a24_i
7a4l_A
7a5q_B
7a5x_A
7a62_B
7a62_C
7a6u_B
7a76_B
7aat_A
7aba_A
7acd_B
7adr_E

7agi_A
7agm_A
7ahd_D
7ahh_A
7an4_A
7anc_A
7anw_D
7aoa_A
7awo_B
7ax6_A
7b08_A
7b16_D
7b1g_A
7bor_C
7bp1_A
7bpc_A
7bpc_C
7bqo_A
7bqu_A
7brn_A
7bs7_A
7bss_C
7bsw_A
7bt2_A
7btc_B
7bv5_A
7bv5_D
7bva_A
7bvd_B
7bvf_A
7bvj_A
7bvy_A
7bwh_A
7bwl_A
7bwr_A
7by6_B
7byw_A
7byx_D
7c1s_A
7c1y_A
7c23_B
7c2b_C
7c3f_S
7c3i_A
7c4h_A
7c79_A
7c7a_K
7c7d_B
7c9r_H
7c9z_D
7ca3_B
7cbb_A
7cbi_A
7cbk_D

7cbs_A
7cfn_R
7cgn_E
7cgp_C
7cgq_C
7cgy_A
7cib_A
7cjd_C
7cka_A
7cm1_B
7cmq_B
7cn3_C
7cn6_B
7cn6_B
7cog_D
7cq5_C
7cqw_A
7ct4_C
7cu1_A
7cv0_A
7cvn_B
7cy5_A
7cz5_R
7cza_B
7d62_A
7d7q_B
7d8u_A
7dh6_B
7dh6_B
7jh4_C
7jhi_B
7jid_B
7jji_A
7job_A
7js3_C
7juq_C
7jur_C
7juv_C
7jux_A
7jwf_A
7k0r_B
7k26_A
7k26_C
7k26_J
7k4f_A
7kb7_A
7kcp_A
7kh2_D
7khs_D
7kjr_A
7ksl_B
7ksm_C
7kz7_A
7l2a_A

7l3o_D
7l3q_B
8gss_C
8rnt_A
8xim_A
8xim_B
9rnt_A
