# Supplementary material for: 3DLigandSite: Structure-based prediction of protein-ligand binding sites
Source: Nucleic Acids Res. Author manuscript; Available in PMC 2022 Jul 6. (PMC9252821; doi:10.1093/nar/gkac250)
Supplement: Supplementary Material [file EMS144313-supplement-Supplementary_Material.pdf]

Supplementary Material

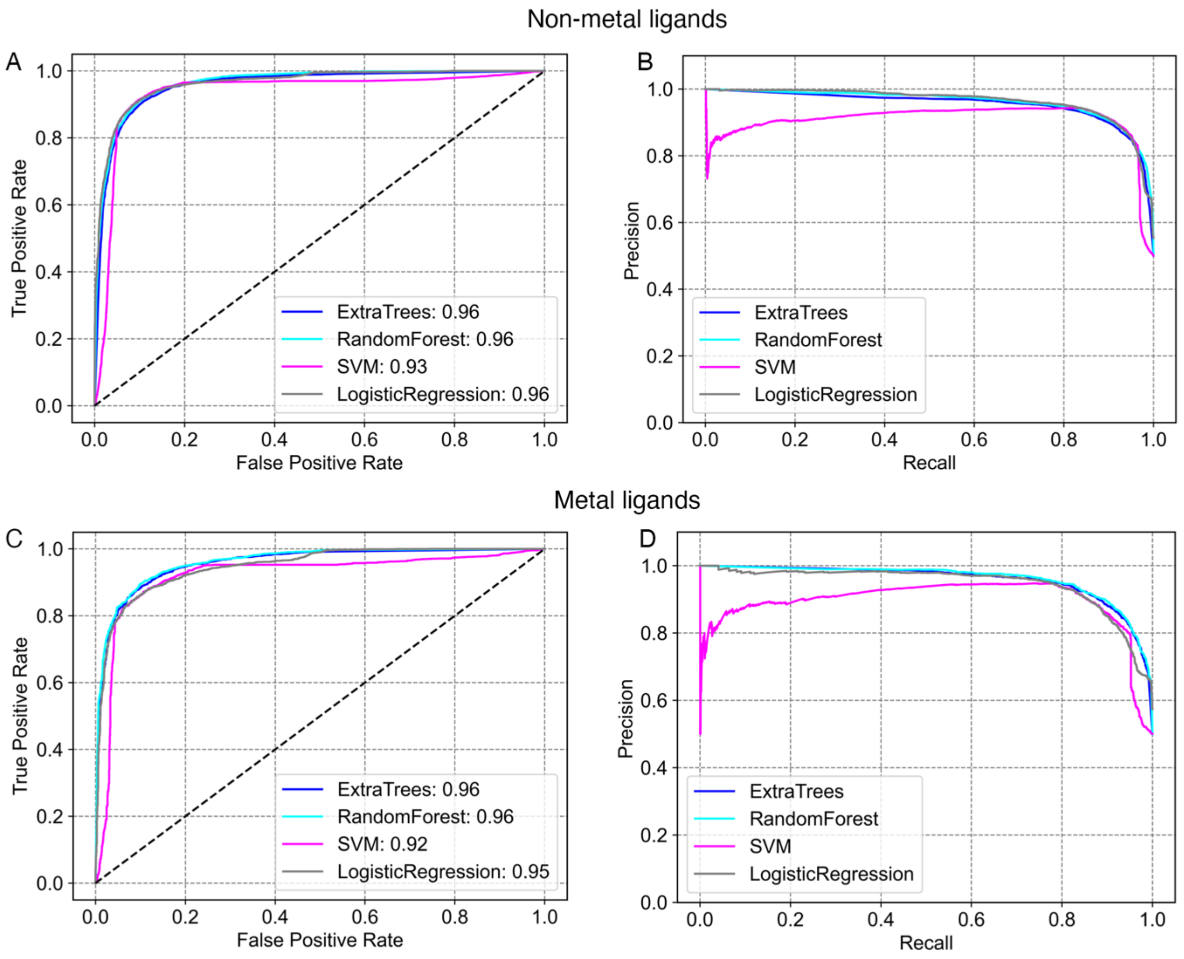

Supplementary Figure 1. Benchmarking of 3DLigandSite on the cross-validation training-testing data. Receiver operator characteristic (ROC) curve and Precision-Recall curve shown for the prediction of binding site of non-metal (A and B) and metal (C and D) binding sites.

Supplementary Table 1. Features used in machine learning

Feature	Value range
js divergence score (conservation)	0-1
Amino acid properties	
Hydrophobicity	0-1
Polar uncharged	0/1 (1 if polar uncharged, 0 otherwise)
Isoelectric point	0-1
Aromatic	0/1 (1 if aromatic, 0 otherwise)
Van der Waals volume	0-1
Positive	0/1 (1 if positive, 0 otherwise)
Negative	0/1 (1 if negative, 0 otherwise)
Amino acid	Each amino acid 1 if present, if not 0. I.e Is tyrosine? 1, Is alanine? 0
3DLigandSite features	
Min ligand distance	0-1 (Value/10, any value greater than 1 is scored as 1)
Max ligand distance	0-1 (Value/10, any value greater than 1 is scored as 1)
Average ligand distance	0-1 (Value/10, any value greater than 1 is scored as 1)
Ligand Contacts	0-1 (Percentage of ligands that the residue is within 0.8/0.4Å + VDW of/100)

Supplementary Table 3. Testing, training, and validation dataset sizes. The training/test set was used for five-fold cross validation using an 80:20 split, with 80% of the data used for training and testing on the remaining 20%.

	**Number of binding sites**	**Number of binding residues**	**Number of non-binding residues**
**Metal binding sites**			
Train/test	1600	1976	1976
Validation	2889	16166	825376
**Non-metal binding sites**			
Train/test	1573	6950	6950
Validation	3527	59203	1044947

Supplementary Table 4. Benchmarking machine learning performance. The performance of four classifiers on datasets are summarised here. ET = Extra-Trees, RF = Random Forest, SVM = Support Vector Machine, LogR = LogisticRegression. Results for **A)** Non-metal ligands and **B)** Metal ligands.

A. Non-metal ligands

	Test			Validation Seq-Search			Validation Struc-Search		
Model	**Precision**	**Recall**	**AUC***	**Precision**	**Recall**	**AUC**	**Precision**	**Recall**	**AUC**
ET	0.9	0.9	0.96	0.85	0.92	0.97	0.83	0.89	0.92
RF	0.9	0.9	0.96	0.86	0.92	0.98	0.84	0.89	0.93
SVM	0.91	0.91	0.93	0.9	0.93	0.94	0.87	0.89	0.9
LogR	0.91	0.91	0.95	0.91	0.92	0.99	0.88	0.89	0.95

*Average from 5-Fold CV

B. Metal ligands

	Test			Validation			Validation Struc-Search		
Model	**Precision**	**Recall**	**AUC***	**Precision**	**Recall**	**AUC**	**Precision**	**Recall**	**AUC**
ET	0.89	0.89	0.96	0.71	0.89	0.96	0.71	0.78	0.84
RF	0.9	0.9	0.96	0.71	0.89	0.96	0.72	0.78	0.85
SVM	0.88	0.88	0.92	0.79	0.89	0.91	0.73	0.78	0.7
LogR	0.88	0.88	0.95	0.79	0.89	0.98	0.73	0.78	0.9

*Average from 5-Fold CV

Supplementary Table 5. CASP Assessment. The performance of the sequence-based and structure-based 3dligandsite tool on the CASP dataset.

**Sequence-based**	**MCC**	**Precision**	**Recall**	**Targets**
HHSearch Prob 75%	0.73	0.65	0.85	70
**Structure Based**				
TMScore 0.5	0.65	0.62	0.71	70
TMScore 0.6	0.72	0.67	0.8	70
TMScore 0.7	0.71	0.68	0.77	70
TMScore 0.8	0.69	0.65	0.77	70
